# Dynamic Network Plasticity and Sample Efficiency in Biological Neural Cultures: A Comparative Study with Deep Reinforcement Learning

**DOI:** 10.34133/cbsystems.0336

**Published:** 2025-08-04

**Authors:** Moein Khajehnejad, Forough Habibollahi, Alon Loeffler, Aswin Paul, Adeel Razi, Brett J. Kagan

**Affiliations:** ^1^ Cortical Labs, Melbourne, Australia.; ^2^ Turner Institute for Brain and Mental Health, Monash University, Clayton, Australia.; ^3^ IITB-Monash Research Academy, Mumbai, India.; ^4^ Wellcome Centre for Human Neuroimaging, University College London, London, UK.; ^5^ Department of Biochemistry and Pharmacology, University of Melbourne, Parkville, VIC 3010, Australia.

## Abstract

In this study, we investigate the complex network dynamics of in vitro neural systems using DishBrain, which integrates live neural cultures with high-density multi-electrode arrays in real-time, closed-loop game environments. By embedding spiking activity into lower-dimensional spaces, we distinguish between spontaneous activity (Rest) and Gameplay conditions, revealing underlying patterns crucial for real-time monitoring and manipulation. Our analysis highlights dynamic changes in connectivity during Gameplay, underscoring the highly sample efficient plasticity of these networks in response to stimuli. To explore whether this was meaningful in a broader context, we compared the learning efficiency of these biological systems with state-of-the-art deep reinforcement learning (RL) algorithms (Deep Q Network, Advantage Actor-Critic, and Proximal Policy Optimization) in a simplified Pong simulation. Through this, we introduce a meaningful comparison between biological neural systems and deep RL. We find that when samples are limited to a real-world time course, even these very simple biological cultures outperformed deep RL algorithms across various game performance characteristics, implying a higher sample efficiency.

## Introduction

Both biological and silicon systems demonstrate the ability to learn and achieve goals, but similarities, differences, and how insights from one may help explain or extend the other remain enigmatic. Biological intelligence is typically treated as the status quo when considering the concept of intelligence. This is natural, as biological intelligence is innate to all humans who may read and understand these sentences. Biological learning can be achieved with minimal power consumption and relatively few trial presentations of novel data [1,2]. The ubiquitous and internalized idea of biological learning and intelligence means that even definitions of what traits a machine learning (ML)-driven artificial intelligence (AI) should demonstrate are heavily informed by traits observed in biological intelligence [3,4]. While the goal of seeing such biological traits in AI systems has not been met, substantial advances have been made across the field of AI in recent years, enabling it to far surpass human capabilities in some tasks such as large-scale pattern recognition. Reinforcement learning (RL), as one example, has become increasingly popular by offering a way of programming agents through reward and punishment cues without having to specify how the task is to be accomplished. However, to deliver on this promise, formidable computational obstacles must be overcome. RL implies learning the best policy to maximize an expected cumulative long-term reward over many steps in order to achieve goals [5]. A deep RL approach integrates artificial neural networks with an RL framework that helps the system to achieve its goals [6]. It maps states and actions to the rewards they bring, combining function approximation and target optimization. Reinforcement algorithms that incorporate deep neural networks have been developed to beat human experts in multiple game settings including poker [7], multiplayer contests [8], complex board games including go and chess [9–11], and numerous Atari video games [12].

Although the complexity of, and drivers behind, biological learning and RL may differ, comparisons between these types of systems can yield valuable insights [13]. Yet, comparisons between biological and machine intelligence have been notoriously difficult, as the scale of connections in even simple biological organisms far exceeds that found in artificial neural networks or comparable ML algorithms [14,15]. Moreover, distinct barriers arise when exploring these learning systems, including but not limited to distinct and sometimes conflicting nomenclature, leading to semantic confusion [16,17]. Examples of real challenges specific to RL include complexities in the selection of hyper-parameters and reward structure, sample inefficiency [18,19], reproducibility issues [20], and catastrophic forgetfulness [21,22]. Furthermore, allowing RL algorithms to train quickly requires considerable levels of computing power [23] with notable associated environmental impacts [24]. Finally, RL algorithms are typically trained for narrow tasks in static environments; where training and performance phases are separate [13,22]. Holistically, these traits suggest that although deep RL algorithms are highly functional, their learning mechanisms almost certainly differ fundamentally from biological learning [13,19,25]. It is noted that RL as a mechanism has been found to elicit rapid and adaptable learning in animals [26,27]. Yet, it seems unlikely that similar underlying statistical mechanisms that support RL, such as back-propagation and gradient descent, have biological parallels in the brain [25,28]. Ultimately, these mechanisms are likely too inefficient to be accepted as plausible models of human learning given the high sample efficiency biological systems demonstrate [18,29,30].

Biological learning systems typically do not display such limitations, although there is substantial uncertainty as to why this is the case. Understanding how neural activity is linked to information processing, intelligence, and eventually behavior is a core goal of neuroscience research. A myriad of approaches have been adopted in vivo exploring the relationship between how structure and function in neural circuits are linked to various cognitive processes and have yielded significant insights and controversies [31–33]. Due to the inherent complexity of in vivo neural systems, it is infeasible to control for all required variables [34], while maintaining appropriate monitoring of the system’s activity and not disrupting the underlying fundamental physiological processes of interest.

In this manner, it may be possible to try and solve one problem with another, using ML approaches to better understand neural dynamics, and taking insights from biological neural dynamics to improve ML methods. To do this, 2 aspects must be addressed. Foremost, as the eventual outcome of neural activity is a combinatorial result of populations of cells working together across a network, being able to study simpler networks that still show evidence of dynamic information processing can be used to provide a considerable advantage to improve our understanding of these systems [35–37]. If simple biological systems show qualitatively different changes in their network dynamics during learning, it may be possible to draw insight from these changes to better understand how biological systems are able to learn so rapidly and power-efficiently. Secondly, it will be necessary to demonstrate that these simple biological systems are capable of showing a competitive advantage in at least one area where RL algorithms struggle. Many RL and AI advocates propose that scaling is all silicon-based systems need to achieve biological intelligence-type properties [38]. If biological learning systems far simpler than those found in small animals show both qualitative differences in how their networks behave during learning, and that this learning can compete with RL models, it would support the idea that quantitative scale alone is not sufficient to yield biological level performance. Rather, qualitative changes to the algorithms, or even the underlying substrate on which the algorithm is run, may be necessary.

To address the first aspect, the information processing and learning potential of in vitro biological neural networks (BNNs) can be explored [39]. Substantial progress has been made developing systems that allow input of structured information to, while recording output from, in vitro neural networks through the use of multi-electrode arrays (MEAs) [40]. Early work established that in vitro neural systems were highly adaptable to electrical stimulation [41–43]. Follow up work demonstrated that closed-loop stimulation, even when not tightly coupled with real-time performance, could create measurable changes in network plasticity and even be integrated with robotics [44–46]. Findings that neural systems may be able to engage in more complex tasks, such as blind source separation [47], spurred the development of more advanced real-time closed-loop systems that successfully demonstrate basic learning in structured information environments in a rapid time course [48,49]. However, despite these advances, the understanding of how simple neural systems reorganize activity in response to incoming structured electrical stimulation remains unclear. Here, we aimed to use data gathered via the previously established DishBrain system to test the adaptive properties of BNNs in a simplified Pong game [49]. Within this framework, in vitro neural networks or cortical cells are combined with in silico computing via high-density MEAs (HD-MEAs). Through real-time closed-loop structured stimulation and recording, self-organized adaptive electrophysiological dynamics are observed [50]. The dynamics that drive these network changes have been well established previously with related patterns being seen to have an effect in clinical studies [51]. Previous work exploring in vitro neural dynamic changes have analyzed functional connectivity and found that simple neural systems are highly adaptable to various types of input [48,52,53]. However, the specific network dynamics integral to the neural learning process, particularly the unit–population relationship, have yet to be fully explored. Neurons in a structured information environment will interact to process information and generate responses. Learning involves the modification of synaptic interactions, which affects the signal transmission within a neural network. Investigating patterns of dynamic interactions between neurons provides insight into the mechanisms underlying learning. Temporal patterns and the strength of these interactions represent the network’s ability to encode, store, and retrieve information. By investigating the dynamic organization of neural networks, we can discover the mechanisms driving synaptic modifications, leading to a deeper comprehension of the cellular and network-level processes intrinsic to learning. Such insights have crucial implications across disciplines, from neuroscience to AI, potentially guiding the development of advanced learning algorithms and treatments for neurological disorders. As such, we aim to study the spiking activity recorded from each channel of the HD-MEA to explore the dynamics of neural network structure and functional connectivity between the recorded units as the game progresses, and compare them with recorded Rest state spontaneous activities. Understanding the complex dynamics of neural networks is important to discover neural mechanisms of how learning occurs.

This exploration becomes even more compelling when we consider the parallels and contrasts between biological learning and ML-based AI systems. Such comparison encapsulates the second aspect of interest we raised—whether observed performance in simple BNNs is notable compared to that of RL at the same task, especially considering sample efficiency. Therefore, our second aim of this work is to investigate whether these elementary learning systems achieve performance levels that can compete with state-of-the-art deep RL algorithms. Additionally, by varying the input information density presented during training of the RL algorithms, we can determine the impact of information sparsity and ensure suitable comparisons to the biological system. To answer these questions, we implement here a synthetic biological intelligence (SBI) system called DishBrain [40] and compare its performance with state-of-the-art RL algorithms. This is the first comparison between SBI and RL systems to our knowledge. We aim to investigate whether simple biological systems can demonstrate characteristics compared to established RL methods to justify further research in this area, either where SBI systems are standalone learning devices, or inform further algorithm development in the ML space. We anticipate that SBI systems will exhibit greater sample efficiency than RL models, as suggested by prior research. However, this entails constraining training to a real-time approximate sample count for RL algorithms. By taking a system-based approach, we aim to compare the data gathered from DishBrain against time-matched learning from deep RL algorithms—Deep Q Network (DQN), Advantage Actor-Critic (A2C), and Proximal Policy Optimization (PPO). Despite the inherent differences between silicon and biological systems—such as power consumption and network size—this approach makes it possible to explore learning performance and sample efficiency in these different systems. Should compelling differences be found, it would further support our efforts to understand key differences in the information processing dynamics unique to each system.

## Materials and Methods

### DishBrain system

DishBrain is a novel system shown to display simple biological intelligence by harnessing inherent adaptive properties of neurons. In DishBrain, in vitro neural networks are integrated with in silico computing via HD-MEAs. These cultured neural networks showcase biologically based adaptive intelligence within a simulated gameplay environment in real time through closed-loop stimulation and recordings [49]. Specifically, BNNs have exhibited self-organized adaptive electrophysiological activity consistent with an innate ability to learn and showcase an intelligent response to limited—although biologically plausible [54]—structured external information. In this study, data were generated from cortical cells obtained from both embryonic rodent and human induced pluripotent stem cell (hiPSC) sources, and we have utilized both groups in our analyses. These cell types were utilized and compared to assess reproducibility of learning effects across species and preparations.

The initial validation of the DishBrain system was previously presented in Ref. [49]. Briefly, cortical cells were either differentiated from hiPSCs using a modified dual SMAD inhibition (DSI) protocol or surgically extracted from E15 mouse embryos. By setting up cultures from multiple cell sources, this helped ensure that results would generalize across different species and preparations. Ethical approvals for animal work were obtained (E/1876/2019/M: Alfred Research Alliance Animal Ethics Committee B) with all cell culture work according to relevant ethical guidelines. Cell line characterization and approvals are reported in Ref. [49].

**Fig. 1.**
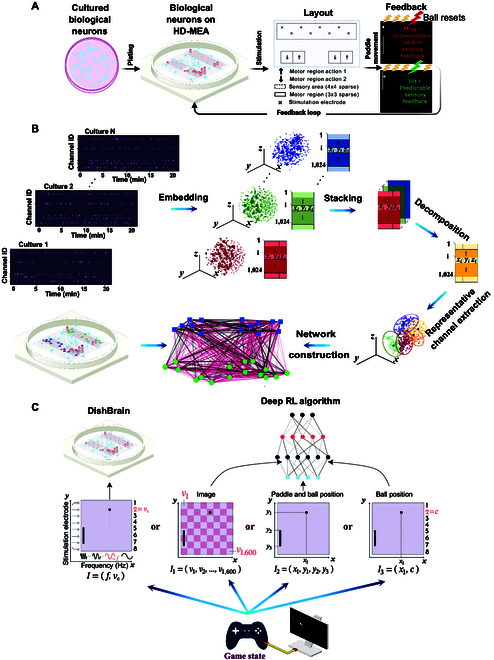
(A) Schematic illustration of the DishBrain feedback loop, the simulated game environment, and electrode configurations. (B) A schematic illustration of the overall network construction framework. The spiking time series data are first transformed into a 3D space using t-SNE embedding. These lower-dimensional representations are then combined into a tensor, which is decomposed using Tucker decomposition. The K-medoids algorithm is then applied to identify consistent representative channels across all cultures. These channels become network nodes, and pairwise Pearson correlation values serve as edge weights. The network layout reflects the physical placement of channels on the MEA, with node colors distinguishing sensory (green) from motor (blue) regions. (C) Schematic comparing the information input routes in the DishBrain system (left) and the 3 implementations of the deep RL algorithms (right). In each design, the input information to the computing module (deep RL algorithms or DishBrain) is denoted by a vector *I*. Note that in the DishBrain system, while this figure depicts stylized waveforms for illustrative purposes, the actual stimulation consisted of discrete electrical pulses.

Approximately 10^6^ cells were plated and integrated onto an HD-MEA (Maxwell Biosystems, AG, Switzerland). Neural cells were either cultured from the cortices of E15.5 mouse embryos or differentiated from hiPSCs via a DSI protocol as previously described [49]. Cells were cultured until plating onto MEA. For primary mouse neurons, this occurred at day-in-vitro (DIV) 0; for DSI cultures, this occurred at between DIV 30 and DIV 33 depending on culture development. Cell cultures were maintained in BrainPhys Neuronal Medium (Stemcell Technologies Australia, Melbourne, Australia) supplemented with 1% penicillin–streptomycin during testing. The DishBrain system was developed as a low-latency, real-time system that interacts with the HD-MEA software to allow closed-loop stimulation and recording, which has previously been described in detail [49]. Using this method, activity from a neural culture can be read, along with providing structured stimulation to the same culture in real time. DishBrain was utilized to embody neural cultures in a virtual game world to simulate the classic arcade game “Pong”. Biphasic electrical stimulation was used to stimulate neurons consistent with previous attempts to elicit action potentials in comparable cultures [55]. Electrical stimulation was arranged to transmit a variety of task-related information between cells and the simulated virtual environment using appropriate coding schemes via routed electrodes on the MEA that were divided into discrete regions. Fig. 1A illustrates the input information, feedback loop setup, and electrode configurations in the DishBrain system.

Specifically, stimulation was applied using pulse trains delivered at varying frequencies (4 to 40 Hz) to encode the position along the *x*-axis (rate coding), and the identity of the stimulated electrode (from a topographically organized set of 8) was used to encode the *y*-axis position (place coding), forming a predefined 2-dimensional sensory input space (see Fig. 1C for the schematic). The voltage of stimulation was set to 75 mV.

Three types of input were provided: the sensory stimulation as explained above, or stimulation in response to activity designated as either “Predictable” or “Unpredictable” feedback (see Fig. 1A). Cultures received Unpredictable stimulation when they missed connecting the paddle with the “ball”, i.e., when a “miss” occurred. Using a feedback stimulus at a voltage of 150 mV and a frequency of 5 Hz, an unpredictable external stimulus could be added to the system. This unpredictable stimulation took place at random sites over the 8 predefined sensory electrodes at random timescales for a period of 4 s, followed by a configurable rest period of 4 s where stimulation paused, then the next rally began. Both the spatial location and the temporal pattern of stimulation during this window were randomized. Should no miss occur, the game would continue until either a miss occurred or the timer of 20 min expired, which would end the session. In contrast, cultures were exposed to Predictable stimulation when a “hit” was registered—that is, when the “paddle” connected successfully with the “ball”. This was delivered across all 8 stimulation electrodes simultaneously at 75 mV at 100 Hz over 100 ms and replaced other sensory information for 100 ms.

The movement of the paddle was controlled by the level of electrophysiological activity measured in a predefined “motor area” of the cultured network, which was collected in real time. Incoming samples were filtered with a second-order high-pass Bessel filter with 100 Hz cutoff. The absolute value was smoothed using a first-order low-pass Bessel filter with a 1-Hz cutoff and the spike threshold is proportional to this smoothed absolute value. A relative activity spike of 6 sigma (Σ) greater than background noise was then used to define an action potential. Detected action potentials from counterbalanced motor regions were then summed together, where higher activity in a given pair of regions would cause the virtual paddle to move in one direction, while activity in the other pair of regions would result in the inverse movement. Information about ball position relative to the paddle was adjusted in a closed-loop manner with a spike-to-stim latency of approximately 5 ms.

The gameplay performance of cell cultures subjected to the simplified Pong environment via the DishBrain system was assessed. In each episode of the game, the average number of rallies before the ball was missed for the first time was then compared with different deep RL baseline methods. Each recording session of the cultures during Gameplay was 20 min. During a Gameplay session, the average number of rallies (i.e., episodes) an average biological culture would perform was 69.04 ± 7.95 rallies or episodes. Therefore, to compare sample efficiency in a matched comparison, a total of 70 training episodes were provided to deep RL algorithms during training.

More details of this system are introduced in Supplementary Sections A.1, A.2, and A.3 as well as Fig. S1.

### Network construction

Recording neural spiking activities occurred across 1,024 HD-MEA channels during 285 Gameplay and 147 Rest sessions (see Fig. 1A for channels’ layout including sensory and motor areas). Due to the extended duration of recordings at a 20-kHz sampling frequency, the resulting time series for Gameplay sessions became notably lengthy. In the context of extracting information from dense and high-dimensional networks, recent emphasis has centered on acquiring network embeddings in lower dimensions. The primary goal of this approach is to obtain vector representations for individual nodes within the network, encapsulating valuable insights [56–58]. Therefore, in this study, we initially employed dimensionality reduction algorithms to enhance computational efficiency for subsequent data analysis and improve data interpretability. This approach also facilitated the revelation of latent data structures not immediately evident in the original high-dimensional space. We utilized t-distributed stochastic neighbor embedding (t-SNE) [59] to generate 2-dimensional representations for both Rest and Gameplay data.

Previous studies have extensively utilized simplified models of interconnected neural populations, employing mean-field approximations. These models effectively retain the dynamic properties of the original neural network while greatly accelerating simulation speeds by several orders of magnitude [60–63]. Furthermore, in complex neural networks, only a fraction of neurons fire at any given time, and many do not exhibit clear action potentials. Recent evidence highlights the emergence of specialized, selective, and abstract response properties in the cortex [64], underscoring the significance of sparse activity and connectivity patterns. These patterns conserve energy and optimize computational capacity [65], emphasizing the redundancy in evaluating individual neuronal firing patterns. The brain’s capacity to encode and process information depends on the concerted action of neural populations, often conveying redundant or highly correlated signals. Given these collective behaviors observed in neural networks, our objective was to advance the reduction of computational complexity when studying large neural populations while still preserving the dynamic properties of the network.

We developed a methodology to identify a subset of 30 recorded channels that likely monitored neural populations specifically tuned to the ongoing task. This subset facilitates the identification of key neurons that characterize the network’s behavior during Gameplay, allowing for a more efficient study of the macroscopic aspects of this smaller and interpretable network. To establish a consistent subset of channels across all neural cultures, we first applied Tucker decomposition to the tensor data derived from the 248 Gameplay sessions, which were represented in a lower-dimensional embedding space of size 3. The resulting 1,024 × 3 tensor built from all channels of all the Gameplay sessions served as a compact representation of the original data, capturing its underlying patterns and structures. Using this tensor, we identified representative channels by applying the K-medoid clustering algorithm, creating *K* = 30 clusters and extracting the corresponding “medoids” for each cluster. Selecting *K* > 30 did not significantly improve the clustering accuracy measured by the Davies–Bouldin index.

Subsequently, a network matrix was constructed using functional connectivity, defined as zero-lag Pearson correlations between the lower-dimensional embeddings of spiking activity across all electrode pairs, for each Gameplay or Rest session recording. Network graphs were constructed from this matrix, with nodes representing 30 channels selected via the described method, and the edges between them representing functional connectivity. Only edges with Pearson correlation absolute values above 0.7 were retained for visualisation.

Fig. 1B is a schematic illustration of the proposed in vitro network construction framework in this study.

### RL algorithms

In this work, we use 3 state-of-the-art deep RL algorithms: DQN [12], A2C [66], and PPO [67], established to have good performance in Atari games. Fig. 1C illustrates the comparison between input information in the DishBrain system and deep RL algorithms. Benefiting from deep learning advantages in automated feature extraction, specifically exploiting convolutional neural networks (CNNs) in their structures, these methods are robust tools in reinforcement tasks, particularly in games where the system’s input is an image. In this work, aiming to account for potential detriments to sample efficiency resulting from the increased dimensionality of the image input to the deep RL algorithms [68], we introduced 2 alternative input designs with significantly lower dimensionality of the input. This enabled us to use shallow architectures for these RL algorithms that are better suited to the simplicity of these input types. We compared all 3 different designs with the performance of biological cultures. We attempt to study whether the curse of dimensionality and increased size of the feature vectors when directly utilizing image inputs affect the comparison between biological cultures and RL algorithms in terms of their sample efficiency. All the algorithms follow a common strategy although they are different in structure. The 3 different input categories and RL algorithm designs are introduced below:

• Image Input: The current state is a tensor of the difference of pixel values from the 2 most recent frames (i.e., another 40 × 40 grayscale pixel image) (this modification led to a noticeable decline in the performance of all the methods because it failed to capture the sense of motion between frames). This current state is then input into the CNN to obtain the selected action. Next, based on the action taken, a reward is received, and a new state is formed. The ultimate goal is to find a policy that indicates the best action in each state to maximize the reward function.
• Paddle & Ball Position Input: Instead of the grayscale image, a 4-dimensional vector encoding the *x* and *y* coordinates of the ball (distance to the paddle/wall and distance to the floor in pixels) and the *y* coordinates of the paddle’s top and the bottom was obtained. All values are integers in the range $\left[4,40\right]$. The current state, which is the input to each algorithm, is then a tensor of the difference of values from the 2 most recent 4-dimensional location vectors. Importantly, in alignment with the simplicity of the input, we did not use any convolutional layers. This setup was explicitly designed as a shallow architecture without any hidden layers; therefore, no additional CNN layer is utilized in this case.
• Ball Position Input: A design as similar to the DishBrain system’s input structure as possible was also examined. For this case, the *y*-axis of the gameplay environment was divided into 8 equal segments each mimicking one of the sensory electrodes in the biological cultures, and place coding the information about the ball’s *y*-axis position as an integer in the $\left[1,8\right]$ interval. Then, the ball’s *x*-axis position is used as the second element of this input vector being an integer value in the range $\left[4,40\right]$ similar to the rate coded component of the stimulation applied to the biological cultures. Similar to above, this setup was explicitly designed as a shallow architecture without any hidden layers. No additional CNN layer is utilized in this design.

An overview of the implemented DQN, A2C, and PPO algorithms and the network architectures is presented in Supplementary Section A.5 (see Algorithms 1, 2, and 3 and Table S1).

All the deep RL implementations were run on a 2.3*-*GHz Quad-Core Intel Core i5. PyTorch 1.8.1 was used to build neural network blocks and an Open AI Gym environment to define our game environment represented by a 40 × 40 pixel grayscale image. In the training phase of all RL algorithms, every algorithm was run for 150 random seeds and a total number of 70 episodes for each seed. These seeds imply 150 different neural networks trained separately to identify the representative performance of the underlying algorithms, resembling 150 different recorded cultures. In this work, we report the average value of each metric among all seeds.

## Results

Neural electrophysiological data from 24 different cultures during 437 experimental sessions (262 “Gameplay”; 175 “Rest”) were recorded at 20-kHz sampling frequency. During Gameplay, the stimulation encoding the ball’s *x*-axis and *y*-axis location and the feedback signal were delivered. During Rest sessions, activity was recorded to move the paddle but no stimulation or feedback was delivered, with corresponding outcomes still recorded. Each Gameplay or Rest session was 20 and 10 min, respectively. During each session, spiking events from sensory and motor channels were extracted.

We aimed to answer the mechanistic question as to whether the observed performance of BNNs in the simulated game environment was accompanied by an equally distinct and rapid system-wide reorganization of neural activity while cells were embodied in a Gameplay environment, versus displaying spontaneous activity during Rest. To explore this question, we analyzed the spiking activity of each HD-MEA channel to assess neural network dynamics and functional connectivity. Understanding these complex dynamics is crucial for uncovering the neural mechanisms behind the efficient learning that occurs in BNNs. We characterized complex network dynamics in in vitro neural systems during 2 distinct activity states: spontaneous activity state with no stimulation (Rest) and engagement in the previously discussed game environment of Pong (Gameplay).

### Functional connectivity analysis

**Fig. 2.**
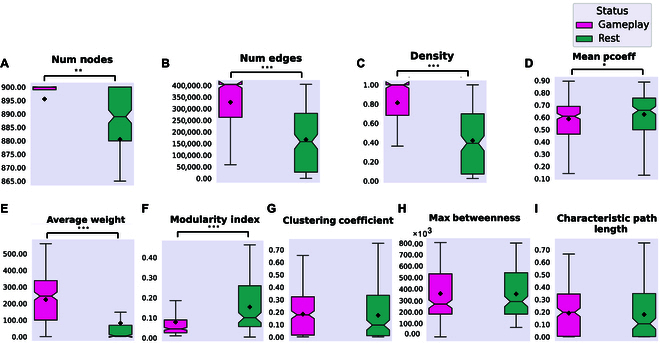
Significant network plasticity occurs in biological cultures when embodied in the game environment. (A to I) Network summary statistics of 1,024 recorded channels using the full duration of all Gameplay and Rest sessions. Using one-way *t* tests, we found significant differences in the number of nodes (*P* = 3.072e−3), number of edges (*P* = 8.396e−26), density (*P* = 1.009e−25), mean participation coefficient (pcoeff) (*P* = 3.400e−2), average weight (*P* = 8.910e−20), and modularity index (*P* = 4.129e−13) between Gameplay and Rest. No significant differences were found for clustering coefficient (*P* = 0.568), max betweenness (*P* = 0.890), or characteristic path length (*P* = 0.533).

A network matrix using functional connectivity—defined as pairwise zero-lag Pearson correlations—among all channels was constructed for the entire duration of all recordings. Fig. 2A to I represent changes in network functional connectivity when comparing the full duration of Gameplay and Rest recordings from all of the 1,024 channels recorded from the HD-MEA. Using one-way *t* tests, significant differences between Gameplay and Rest were found for the number of nodes, number of edges, density, mean participation coefficient (pcoeff), average weight, and modularity index. No significant differences were found for clustering coefficient, max betweenness, and characteristic path length.

As the next step, we performed a region-based analysis by grouping electrodes into predefined functional zones (Motor Up and Down and 8 Sensory segments) to examine large-scale sensorimotor connectivity patterns using Pearson correlations (see Supplementary Section A.4 and Figs. S2 to S4). While this approach revealed condition-dependent differences between Rest and Gameplay, the spatial averaging across all electrodes in each region limited our ability to resolve fine-grained, functionally specific dynamics. This limitation was mainly due to biological variability across cultures, including differences in where neurons adhere and which subsets become functionally active. As a result, averaging across all electrodes in a region diluted the contribution of active subpopulations, masking important task-related network reconfigurations.

**Fig. 3.**
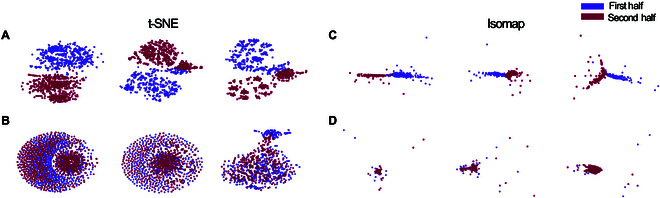
Low-dimensional representation of 3 samples of (A) Gameplay sessions and their following (B) Rest sessions using t-SNE as well as (C) Gameplay sessions and their following (D) Rest sessions using Isomap. The purple and maroon dots are the channel representations in the embedding space in the first and second halves of the recordings, respectively. Both dimensionality reduction algorithms were able to distinguish between the 2 halves of recording during Gameplay but not during Rest sessions.

Motivated by the limitations of region-based averaging, we aimed to investigate these finer-scale dynamics more directly. Leveraging the growing interest in uncovering structure within complex neural systems through low-dimensional representations [56–58], we applied nonlinear dimensionality reduction techniques—t-SNE [59] and Isomap [69]—to visualize changes in functional connectivity over time. For this, we first divided each recording session into early and late halves and computed embeddings of the corresponding connectivity matrices. Results presented in Fig. 3A and B and Fig. 3C and D showcase t-SNE and Isomap outcomes with color-coded distinctions for the initial and latter portions of 20-min Gameplay and 10-min Rest sessions across 3 representative samples. Discernible patterns emerge in Gameplay but not in Rest, signifying distinctive network dynamics during the learning process, predominantly observed in Gameplay, which was effectively captured in this lower dimensional space.

To take a step further, in light of previous findings that in complex neural networks only a subset of neurons becomes active at any given moment and many do not exhibit distinct action potentials [64], our objective was to enhance the reduction of computational complexity when studying these neural populations while maintaining the dynamic properties of the network. Utilizing the method introduced in Ref. [70], we identified a subset of key channels (30 channels) characterizing the network’s behavior during Gameplay, to more efficiently study this smaller and more interpretable network.

Next, by utilizing these low-dimensional representations, we recreated functional connectivity matrices from these 30 channels as nodes and edges represented by Pearson correlations as described previously.

Motivated by this emergent and collective behavior of neural networks, we aimed to advance the reduction of computational complexity, when studying large neural populations while simultaneously preserving the dynamical properties of the network. To do so, we developed a method to identify a subset of recorded channels that likely monitored the neural populations specially attuned to the ongoing task. Such a subset allows for specification of channels that characterize the network’s behavior during Gameplay to more efficiently study the (macroscopic) dynamics of this smaller and interpretable network of channels (see the “Network construction” section for full details). A network matrix using functional connectivity—defined as the zero-lag Pearson correlations—of each Gameplay or Rest session recording was constructed with 30 representative channels as the nodes and the edges between these nodes represented by the functional connectivity.

After constructing the connectivity networks, we aimed to examine their temporal evolution in both Gameplay and Rest. To achieve this, we divided each recording session into 2-min windows and evaluated the change in edge weights as the network evolved over those windows.

**Fig. 4.**
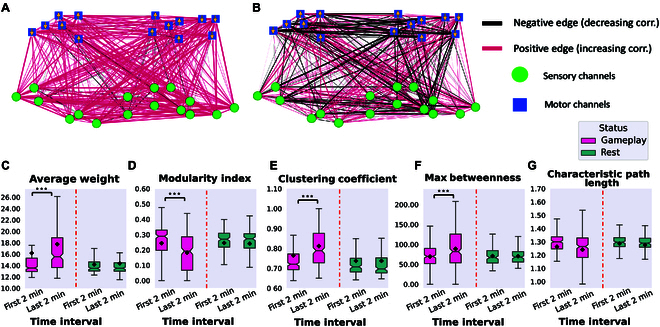
The average connectivity networks using 30 representative channels over all (A) Gameplay and (B) Rest sessions with edge weights representing changes in functional connectivity between channel pairs when comparing the last 2 min to the first 2 min of recordings. Edge colors signify the direction of these connectivity changes, with red indicating increases and black indicating decreases. Motor and sensory region channels are represented by blue squares and green circles, respectively. Arrows on motor region nodes show the paddle’s movement direction as per their position in the predefined layout in Fig. 1. (C to G) Network summary statistics between the first and last 2 min of Gameplay and Rest recordings using the 30 representative channels in the lower-dimensional space. All of these metrics except the characteristic path length showed statistically significant differences using one-way ANOVA during Gameplay (*P* = 2.265e−3, *P* = 8.478e−8, *P* = 1.891e−6, *P* = 1.005e−4, and *P* = 0.071, respectively), but not in the Rest condition of the cultures (*P* = 0.864, *P* = 0.670, *P* = 0.738, *P* = 0.281, and *P* = 0.899, respectively). ***P < 0.001.

Fig. 4A and B show differences in the correlation between each pair of nodes when comparing the last and first 2 min of each recording. This figure shows the average networks over all Gameplay or Rest sessions with red/black colors indicating increased/decreased correlations, respectively. The edge weights are proportional to the absolute value of these differences in functional connectivity.

We found that biological cultures, while embedded in the game environment, had a higher number of edges with increased correlation between channels. This change was not apparent during Rest state spontaneous activity. This indicates significant network plasticity in these cultures that can be a necessary underlying mechanism for the learning that happens in this closed-loop system [49]. Moreover, we evaluated the network characteristics from all generated networks and compared them between the first and last 2 min of recordings in both Rest and Gameplay groups. Fig. 4C to G show these results. All of these metrics except characteristic path length showed statistically significant differences during Gameplay, but not in Rest. In particular, the average weight of the networks only shows a significant increase in the Gameplay sessions and the modularity index significantly decreases only during Gameplay. A higher modularity index indicates the presence of many connections within a community but only few with other communities, while a lower index means higher outward connections between different communities.

### Comparison in performance between DishBrain and 3 RL algorithms with various information densities

Next, we compared the game performance of human cortical cells (HCCs; 174 sessions) and mouse cortical cells (MCCs; 110 sessions) with 3 RL baseline methods. To determine how learning arises both in cultures and in baseline methods, 3 key gameplay characteristics were examined. These include mean hit-to-miss ratio (average hits-per-rally), number of times the paddle failed to intercept the ball on the initial serve (aces), and number of long rallies or episodes (≥3 consecutive hits).

**Fig. 5.**
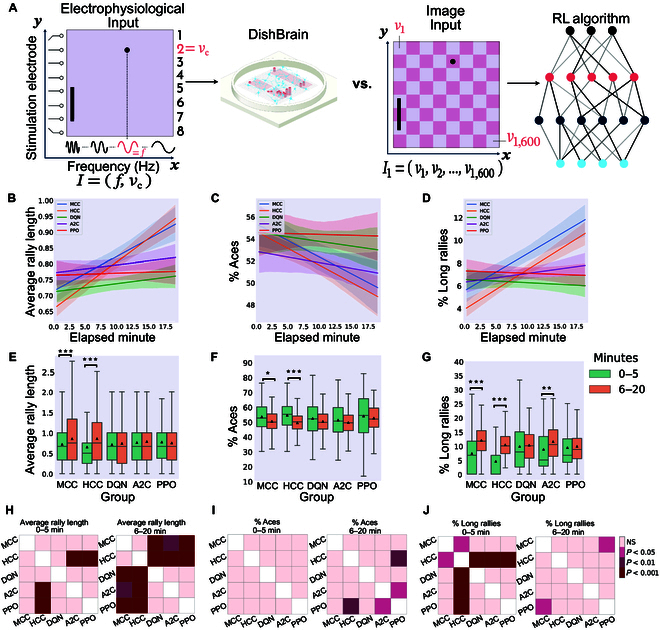
Image Input to the deep RL algorithms. (A) Schematic highlighting figure comparisons are between biological DishBrain system and a pixel-based information input to the RL algorithms. Average number of (B) hits-per-rally, (C) % of aces, and (D) % of long rallies over 20 min real-time equivalent of training DQN, A2C, PPO, MCC, and HCC cultures. A regressor line on the mean values with a 95% confidence interval highlights the learning trends. Comparing the performance among all groups, the highest level of average hits-per-rally is achieved by the neural MCC and HCC cultures while PPO is outperformed by all the opponents. The average % of aces is lowest for the neural cultures compared to all deep RL baseline methods. The average % of long rallies reaches its highest levels for MCC and HCC. (E) Average performance of groups over time. Only biological cultures have significant within-group improvement and increase in their performance at the second time interval (one-way ANOVA test, *P* = 5.854e−6 and *P* = 7.936e−17 for MCC and HCC, respectively; *P* = 0.231, *P* = 0.318, and *P* = 0.400 for DQN, A2C, and PPO respectively). (F) Average % of aces within groups and over time. Only MCC and HCC (one-way ANOVA test, *P* = 0.014 and *P* = 2.907e−8, respectively) differed significantly over time. No significant change was detected within the DQN, A2C, or PPO groups (one-way ANOVA test, *P* = 0.080, *P* = 0.195, and *P* = 0.308, respectively). (G) Average % of long-rallies (≥3) performed in a session. All groups showed an increase in the average number of long rallies where this within-group increase was significant only for MCC, HCC, and A2C (one-way ANOVA test, *P* = 1.172e−7 and *P* = 1.525e−24 for MCC and HCC, respectively and *P* = 0.605, *P* = 0.002, and *P* = 0.684 for DQN, A2C, and PPO, respectively). *P < 0.05, **P < 0.01, and ***P < 0.001. (H) Pairwise Tukey’s post-hoc test shows that HCC and MCC groups significantly outperform PPO, A2C, and DQN in the last 15-min interval. (I) Using pairwise Tukey’s post-hoc test, the HCC group significantly outperforms the PPO in the last 15-min interval with a lower average of % Aces. A2C also outperforms PPO in this time interval. (J) Pairwise comparison using Tukey’s test only shows a significant difference in the percentage of long rallies between HCC and the rest of the groups in the first 5 min. However, this is later altered in the direction of all groups having an increased % of long rallies with MCC outperforming PPO in the last 15 min of the game. Box plots show interquartile range, with bars demonstrating 1.5× interquartile range; the line marks the median and the black triangle marks the mean. Error bands = 1 SE.

**Fig. 6.**
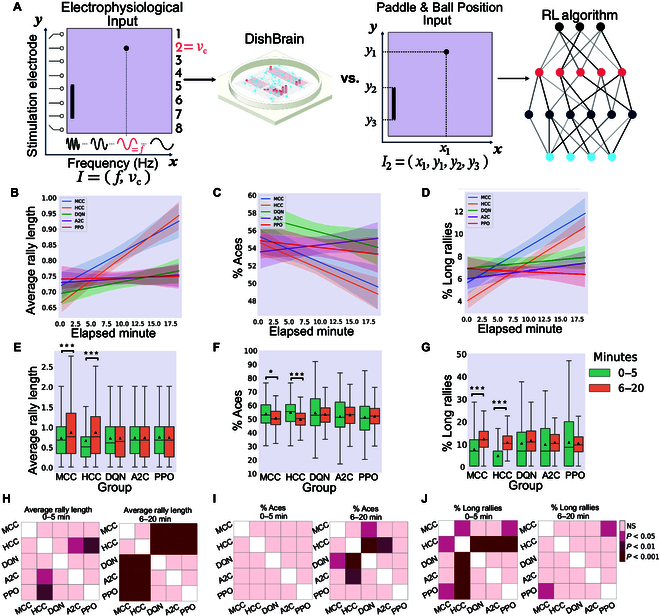
Paddle & Ball Position Input to the deep RL algorithms. (A) Schematic highlighting figure comparisons are between biological DishBrain system and Paddle & Ball Position Input to RL algorithms. Average number of (B) hits-per-rally, (C) % of aces, and (D) % of long rallies over 20 min real-time equivalent of training DQN, A2C, PPO, MCC, and HCC cultures. A regressor line on the mean values with a 95% confidence interval highlights the learning trends. The highest level of average hits-per-rally is achieved by the MCC and HCC cultures. The average % of aces is lowest for the neural cultures compared to all deep RL baseline methods. The average % of long rallies reaches its highest levels for MCC and HCC. (E) Average rally length over time only showed a significant increase in the biological cultures between the 2 time intervals (one-way ANOVA test, *P* = 0.913, *P* = 0.958, and *P* = 0.610 for DQN, A2C, and PPO, respectively). (F) Average % of aces within groups and over time only showed a significant difference in the MCC and HCC groups. No significant change was detected within the DQN, A2C, or PPO groups (one-way ANOVA test, *P* = 0.463, *P* = 0.338, and *P* = 0.544, respectively). (G) Average % of long-rallies (≥3) performed in a session increased in the second time interval in all groups. This within-group difference was only significant for the MCC and HCC groups (one-way ANOVA test, *P* = 1.172e−7, *P* = 1.525e−24, *P* = 0.233, *P* = 0.320, and *P* = 0.650 for MCC, HCC, DQN, A2C, and PPO, respectively). *P < 0.05 and ***P < 0.001. (H) Pairwise Tukey’s post-hoc test shows that the HCC group is significantly outperformed by A2C and PPO in the first 5 min in terms of the hit counts. Biological cultures, however, do significantly better compared to all deep RL opponents in the 15-min interval. (I) Using pairwise Tukey’s post-hoc test, the HCC group significantly outperforms the DQN and A2C groups in the last 15-min interval with a lower average of % Aces. DQN is also outperformed by the MCC group in this time interval. (J) Pairwise comparison using Tukey’s test shows a significant difference in the percentage of long rallies between HCC and the rest of the groups in the first 5 min all outperforming the HCC. However, this is later altered in the last 15 min with only MCC outperforming PPO significantly having an increased % of long rallies. Box plots show interquartile range, with bars demonstrating 1.5× interquartile range; the line marks the median and the black triangle marks the mean. Error bands = 1 SE.

**Fig. 7.**
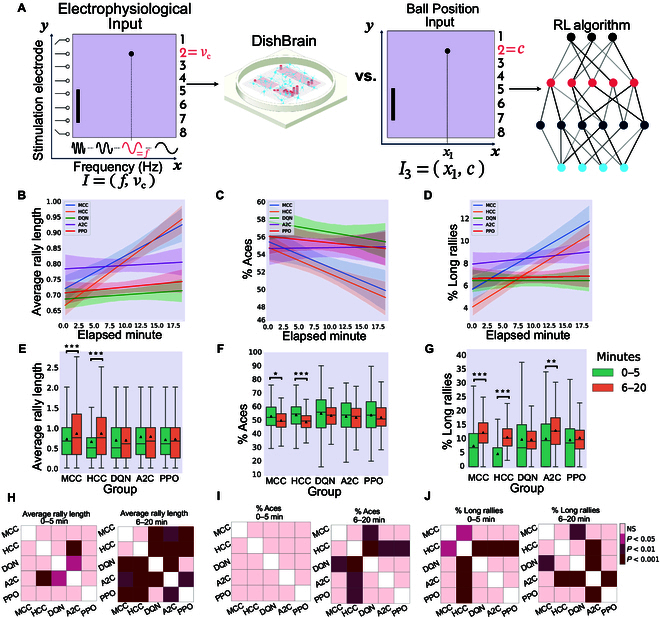
Ball Position Input to the deep RL algorithms. (A) Schematic highlighting figure comparisons are between biological DishBrain system and Ball Position Input to RL algorithms. Average number of (B) hits-per-rally, (C) % of aces, and (D) % of long rallies over 20 min real-time equivalent of training DQN, A2C, PPO, MCC, and HCC cultures. A regressor line on the mean values with a 95% confidence interval highlights the learning trends. The highest level of average hits-per-rally is achieved by the neural MCC and HCC cultures. The average % of aces is lowest for the neural cultures compared to all deep RL baseline methods. The average % of long rallies reaches its highest levels for MCC and HCC. Comparing to the same findings for the HCC and MCC groups, (E) average rally length over time only showed a significant increase in the biological cultures between the 2 time intervals (one-way ANOVA test, *P* = 0.995, *P* = 0.812, and *P* = 0.547 for DQN, A2C, and PPO, respectively). (F) Average % of aces within groups and over time only showed a significant difference in the MCC and HCC groups. No significant change was detected within the DQN, A2C, or PPO groups (one-way ANOVA test, *P* = 0.241, *P* = 0.581, and *P* = 0.216, respectively). (G) Average % of long rallies (≥3) performed in a session increased in the second time interval in all groups except DQN. This within-group difference was only significant for MCC, HCC, and A2C groups with *P* = 0.002 for the A2C group. *P < 0.05, **P < 0.01, and ***P < 0.001. (H) Pairwise Tukey’s post-hoc test shows that biological cultures significantly outperform all deep RL groups in the last 15 min in terms of the hit counts or rally length. (I) Using pairwise Tukey’s post-hoc test, the HCC group significantly outperforms all the deep RL groups in the last 15-min interval while MCC also outperforms DQN with a lower average of % Aces. (J) Pairwise comparison using Tukey’s test shows a significant out-performance of all groups over HCC in the percentage of long rallies in the first 5 min. In the second time interval, MCC shows a significantly higher % of long rallies compared to DQN with HCC now being outperformed only by A2C. Box plots show interquartile range, with bars demonstrating 1.5× interquartile range; the line marks the median and the black triangle marks the mean. Error bands = 1 SE.

For comparison, every 70-episode run of each RL algorithm was mapped to approximately 20 real-time minutes by normalizing the actual total length of each run in minutes and then multiplying by 20. This approximates the number of rallies biological cultures would experience in a 20-min session. Details of the implemented RL algorithms and information about the selected hyper-parameters are included in Supplementary Section A.5. Figs. 5, 6, and 7 represent the main findings for comparisons between biological cultures and the Image Input, Paddle & Ball Position Input, and Ball Position Input designs of the RL methods. The intent behind different input designs was to determine whether varying the amount of information input into the algorithm altered the sample efficiency and learning characteristics of these systems. In particular, the Paddle & Ball Position Input and Ball Position Input methods were intended to be more accurate comparisons to the information density presented to the DishBrain system. Tables S3 and S4 present all multivariate statistical tests performed in relation to the following results. All post-hoc follow-up tests are presented in Table S2.

### Comparison in performance between DishBrain and 3 RL algorithms with various information densities

In all 3 designs, biological cultures (i.e., HCC and MCC) outperformed all RL baseline algorithms (see Figs. 5B, 6B, and 7B) in terms of the highest level of average hits-per-rally achieved. The cultures demonstrated faster learning rates over time. Figs. 5C, 6C, and 7C compare the % of aces among the biological cultures and the RL groups given the 3 different designs. HCC and MCC achieved the lowest percentage of aces compared to the deep RL algorithms in Fig. 5C and the other RL baseline designs in Figs. 6C and 7C. The increasing trend in % of long rallies is observed in all groups and among all designs except the DQN and PPO groups in the Image Input design and PPO in the Paddle & Ball Position Input design, as illustrated in Figs. 5D, 6D, and 7D. Average % of long rallies was highest for MCC and HCC compared to RL baselines.

Key activity metrics in the first 5 min versus the last 15 min in each session were compared to identify any significant improvement occurring in the learning process within each group.

Panel E in Figs. 5, 6, and 7 compares average rally length between the 2 defined time intervals within groups. The results indicate that the within-group increasing trend in rally length was significant only in the biological groups.

Panel F in Figs. 5, 6, and 7 represents the change in average percentage of aces over time. A significant decrease in number of aces (where the ball was missed immediately in an episode with no accurate hits) indicates improved game performance. Only MCC and HCC had a significant decrease in average ace percentage as opposed to the rest of RL-based algorithms with different input designs.

Panel G in Figs. 5, 6, and 7 shows that the percentage of long rallies in the first 5 min versus the last 15 min only significantly increased for biological cultures and A2C with the Image Input and Ball Position Input designs.

Inter-group comparison was carried out for both time intervals (0 to 5 min and 6 to 20 min) and all 3 metrics using Tukey’s post-hoc test as represented in panels H, I, and J in Figs. 5, 6, and 7 for rally length (i.e., hit counts), % of aces, and % of long rallies, respectively.

Note that, in the Image Input design, the input information density is starkly different between the 2 groups. While RL agents received pixel data with a density of 40 × 40 pixels, biological cultures only receive input from 8 stimulation points with a given integer rate code of 4 to 40 Hz, highlighting important efficiency differences in informational input between these learning systems. The possibility of higher input information dimensionality having adverse effects on overall sample efficiency by increasing the samples required for the model convergence of RL algorithms is further nullified by evaluating 2 alternative input structures (Paddle & Ball Position Input and Ball Position Input designs).

### Examining impact of paddle movement speed on learning rates

**Fig. 8.**
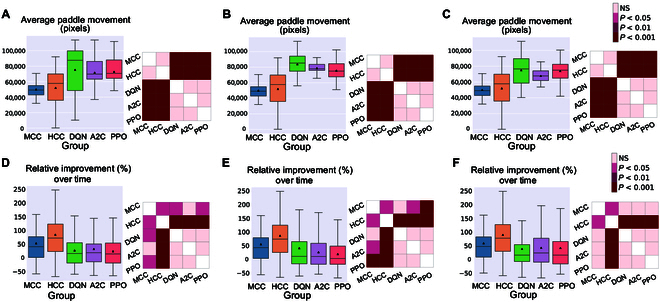
Paddle movement and relative improvement. The average paddle movement in pixels in all the different groups for the (A) Image Input, (B) Paddle & Ball Position Input, and (C) Ball Position Input to the deep RL algorithms. Tukey’s post-hoc test was conducted showing that DQN, PPO, and A2C had a significantly higher average paddle movement compared to HCC and MCC in all scenarios. Relative improvement (%) in the average hit counts between the first 5 min and the last 15 min of all sessions in each separate group for the (D) Image Input, (E) Paddle & Ball Position Input, and (F) Ball Position Input to the deep RL algorithms. The biological groups show higher improvements with HCC outperforming all. (D) Using Games Howell post-hoc test, the inter-group differences were significant with HCC outperforming all other groups, as well as MCC significantly outperforming PPO. (E) HCC showed a significantly higher relative improvement compared to all the other groups while MCC also outperformed A2C and PPO in terms of relative improvement over time. (F) Finally, HCC could still perform significantly better than all the deep RL groups with the Ball Position Input design to the deep RL algorithms with MCC outperforming PPO and DQN in this design.

To account for potential effects of paddle movement speed and whether it plays an important role in determining the success rate of paddle control, we derived the average paddle movement (in pixels) for all groups. Fig. 8A to C represent these results for the Image Input, Paddle & Ball Position Input, and Ball Position Input designs, respectively. Using Tukey’s post-hoc tests, a consistently significant difference between pairs of DQN, PPO, or A2C with MCC or HCC was found in terms of average paddle movement, with RL algorithms having the higher average. This occurs when all the RL algorithms with different input designs have significantly higher average paddle movement compared to both groups of biological cultures. As per previous findings [49], increased paddle movement speed in RL algorithms does not translate to improved game performance, likely suggesting a more stochastic paddle control. Fig. S5 compares frequency tables for distributions of mean summed hits per minute among groups for the Image Input, Paddle & Ball Position Input, and Ball Position Input designs, respectively. These tables were not significantly different (two-sample *t* test).

Fig. 8D to F compare relative improvement in performance between biological cultures and RL algorithms for Image Input, Paddle & Ball Position Input, and Ball Position Input, respectively. This measure identifies the relative increase in average accurate hit counts in the final 15 min of the game compared to the first 5 min. The HCC group showed the highest improvement in time. Post-hoc tests showed significant differences between HCC and all the RL methods across all of the 3 different input designs. The MCC group also outperformed PPO in both Image Input and Paddle & Ball Position Input designs as well as DQN and A2C in the Image Input and Paddle & Ball Position Input designs, respectively.

**Fig. 9.**
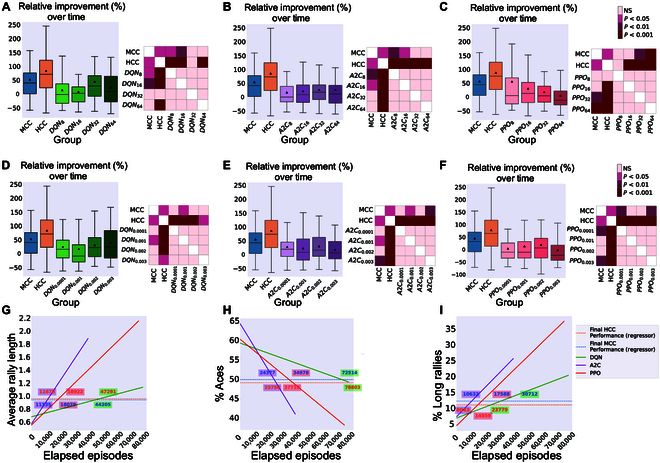
Batch size and learning rate effects for the RL algorithms with Image Input. Relative improvement (%) in the average hit counts between the first 5 min and the last 15 min of all sessions as well as the post-hoc tests in each separate group for batch sizes of 8, 16, 32, and 64 in the (A) DQN, (B) A2C, and (C) PPO groups compared to biological cultures. Games Howell post-hoc tests show the inter-group differences that were not significant between any pair of different batch sizes for any of the DQN, A2C, or PPO groups. Relative improvement (%) in the average hit counts between the first 5 min and the last 15 min of all sessions for learning rate (critic learning rate for PPO and A2C algorithms) values of 0.0001, 0.001, 0.002, and 0.003 in the (D) DQN, (E) A2C, and (F) PPO groups compared to biological cultures. Games Howell post-hoc tests show the inter-group differences that were not significant between any pair of different batch sizes for any of the DQN, A2C, or PPO groups while the HCC group significantly outperforms all RL algorithms in this measure. (G to I) Extended training episodes for the deep RL algorithms. Illustrating the performance of a single DQN, A2C, and PPO with the Image Input over an extended number of training episodes, measured in terms of (G) average rally length, (H) % of aces, and (I) % of long rallies. The final performance levels of the HCC and MCC groups are shown with dashed horizontal lines, and the episode numbers at which these RL algorithms surpass these levels are annotated on the plots.

Details of the implemented RL algorithms and hyper-parameters can be found in the data repository provided in the Data Availability section. In summary, a comprehensive grid search was conducted within the parameter space of learning rate, replay buffer size, and the training batch size aiming to identify the optimal parameter configuration and it was found that similar results were obtained across a variety of hyper-parameters, strongly supporting the initial conclusions of this work. Here, we include the example of how increasing the batch size affects the overall performance of the RL algorithms, while keeping the rest of the parameters set to their initial values in the search space. (We have thoroughly documented the grid search process, including the full hyper-parameter search space, selected values, and performance comparisons with alternative configurations. For further details and exploration of selected hyper-parameters, see Supplementary Sections A.5 and A.6, Table S1, and Figs. S6 to S11.) In general, we observed some quantitative changes in outcome metrics when varying the batch size for these algorithms, but these adjustments did not alter the ultimate conclusions of our work. Focusing on the quality of learning in each group and the comparison of sample efficiency, both of these were unaffected or in some cases worsened by increasing the batch size. Specifically, when examining the changes in average accurate hit counts during the first 5 min versus the last 15 min of training and the overall relative improvement, the increased batch size did not appear to significantly impact the resulting sample efficiency in any of the algorithms as seen in Fig. 9A to C.

Finally, we investigated the effects of changing the learning rate for the DQN algorithm and the critic learning rate for the A2C and PPO algorithms, which are both parameters that control the step size for updating the value function during the training phase of these RL algorithms. Similar to the above, when examining the overall relative improvement, the increased learning rate (or critic learning rate) did not appear to significantly impact the resulting sample efficiency in any of the algorithms as seen in Fig. 9D to F. Additionally, the relative improvement of the HCC group significantly outperformed all variations of the 3 RL algorithms across different settings of this parameter.

Finally, Fig. 9G to I illustrate the average rally length, % of aces, and % of long rallies for the RL algorithms with the Image Input, using the same hyper-parameter sets as in Fig. 5, when trained for a significantly extended number of game episodes. These results reveal that all 3 algorithms successfully learned and showed improved performance over the extended training period. Training the implemented deep RL algorithms for tens of thousands of game episodes with the same set of hyper-parameters as in Results demonstrates a continuous increase in their performance, eventually surpassing the performance levels of the biological cultures (indicated by dashed horizontal lines). However, as expected, 70 game episodes—the same number used to train the biological cultures—were not sufficient for any of the algorithms. As part of this reason may be explained by the sample inefficiency of back-propagation, we also implemented a biologically inspired algorithm with an active inference agent that uses counterfactual learning, and reported the comparison results in Supplementary Section A.7 and Fig. S12. Improved learning rates observed in the biological inspired learning protocol supports the potential of active inference agents to provide valuable insights into optimized learning strategies, thereby enhancing our understanding of these dynamics. However, these active inference algorithms are still highly dependent on the chosen hyper-parameters and require relatively higher power consumption compared to biological systems.

## Discussion

Understanding how biological systems process information and even demonstrate learning in comparison to silicon-based computational algorithms is a substantial challenge. One issue has been that biological systems are usually highly complex and are typically inherently integrated within living organisms that have important interacting features. Therefore, comparing even simple biological intelligences that are part of living organisms to machine learning algorithms is difficult. Moreover, the interplay between individual neural activity and population-level activity adds further complexity to determining the mechanisms of learning within biological cultures. Here, we built upon previous work that demonstrated observing learning in an in vitro BNN is possible [49]. Despite this proof of concept, the network dynamics responsible for gameplay performance have only explored population-level activity [50], not differences in network connectivity. By exploring how functional connectivity changes occur within simple BNNs in relatively simple structured information landscapes, it was possible to observe if dynamic network changes would occur over time. In this study, we analyzed the activity of neural populations by embedding them in lower-dimensional space and identifying a specific subset of the population that can encapsulate the full network dynamics. This approach was inspired from mean-field theory, a technique used in statistical physics that simplifies network models to reduce computational complexity [71,72]. Building on prior findings, we acknowledge that redundancy within large populations of neurons contributes to network robustness, and only a subset of neurons is often necessary to capture the comprehensive dynamics of the network. This principle underpins our analysis of a large population of in vitro cortical neurons within a closed-loop game environment using the DishBrain system, where we investigate network dynamics during learning and compare them to Rest conditions. This method enabled us to observe underlying network dynamics that facilitate information processing and learning.

By introducing a novel framework and demonstrated its efficacy in extracting low-dimensional representations and identifying influential recorded units, it was possible to differentiate between Rest and Gameplay conditions based on functional connectivity metrics. This efficiency in capturing variations in network properties advances our understanding of how large neural populations function and adapt in complex environments, revealing potential mechanisms underlying learning. These findings build upon earlier work that has established the value of reducing temporal and spatial dimensions of data to better understand prevailing trends [59,69]. The result provides preliminary evidence but compelling support for the notion that reorganization of subunits within the neural system into assemblies that are able to more reliably engage in computation [73,74]. In particular, as the DishBrain setup displayed discrete input and output regions as part of the closed-loop feedback, observing the specificity of these connections over time is a compelling extension over previous work investigating functional plasticity in neural cultures [48,75]. Although limitations in study design (specifically, the use of opaque chips which limited visualizing physical connections) prevented a robust assessment of the specific learning processes within the cultures used in this study beyond that previously reported [49,50], these findings endorse this approach for future exploration of these dynamics with altered study designs and more advanced tools [76]. Additionally, future work would benefit from exploring explicit control over structural and functional connectivity of BNNs as has been demonstrated previously [75,77]. Such an approach coupled with a closed-loop real-time environment would allow more explicit tests to explore the structure–function relationship between functional connectivity and information processing. Future work with controlled neural structures could have the potential to understand not only how biological intelligence arises, but also how one may implement more advanced biological learning protocols that may surpass current performance [39].

To extend our work, given the previous parallels drawn between connectivity maps in biological neural systems and those in artificial neural networks (ANNs) [14], we also compared whether the observed behavior of these BNNs could compete with RL-based algorithm sample efficiency in learning a task. Here, we have provided a small step forward by comparing BNNs with deep RL models on a similar task. The selection of HCCs and MCCs was driven by their distinct developmental and plasticity characteristics, allowing us to explore whether the observed learning dynamics are conserved across species and neuronal origins. Similarly, the 3 deep RL algorithms—DQN, A2C, and PPO—were chosen based on their established success in solving RL tasks, with DQN representing a value-based model and A2C/PPO leveraging policy-gradient approaches optimized for sample efficiency. We demonstrated that the DishBrain system outperforms these state-of-the-art deep RL algorithms in a game of Pong in sample efficiency when limited to a real-world time course. By implementing this methodology, we aimed to compare biological and AI systems in terms of performance and sample efficiency. The advantages and disadvantages of biological versus machine intelligence are often discussed, yet technical limitations have prevented meaningful comparisons in terms of performance. By limiting RL models to a real-world time course, we were able to compare key traits between BNNs and RL models with a focus on sample efficiency. HCCs or MCCs along with 3 deep RL algorithms (DQN, A2C, and PPO) were compared in sessions with an average episode number of 70 games played. The aim of this work was to determine whether meaningful performance differences would arise between learning systems (as contained systems) that may merit further exploration of BNNs as information processing machines. Our approach allowed examination of the overall performance of each group with respect to various gameplay characteristics and, for the RL methods, in response to varying dimensionality of the information input.

Across all types of information input, BNN outperformed all RL baselines in terms of average hit-per-rally (Fig. 5A), % of aces (Fig. 5B), and % of long rallies achieved (Fig. 5C). Moreover, the increase in average rally length, decrease in number of aces, and increase in number of long rallies were significant only within the HCC and MCC groups and the A2C algorithm with the Image Input and Ball Position Input designs in terms of the increase in the percentage of long rallies, when comparing the first 5 and the last 15 min during Gameplay (see Fig. 5D to F). Additionally, we found that the HCC group had the highest relative improvement in average number of hits between the first 5 min and last 15 min of the game as depicted in Fig. 8B, D, and F.

Collectively, these results showed that the game performance of deep RL algorithms in terms of relative learning improvement in time and average hits-per-rally is outperformed by biological cultures when number of allowable samples are fixed. This supports the conclusion that RL algorithms showed significantly lower sample efficiency compared to BNN, having lower improvements in learning over an episode-matched training duration provided for all groups. This matches theoretical expectations previously outlined where it was proposed that biological learning is inherently more sample efficient [13,25]. Given how rapidly synaptic plasticity or behavior changes have occurred for both in vitro and in vivo models, this finding is consistent with such observations [26,27,44,45,50]. Furthermore, although difficult to directly compare energy consumption, it should be noted that biological systems use magnitudes less powerful than traditional computing systems used for ML [78]. Moreover, comparison between various ML algorithms was also consistent with past research. A2C and PPO achieved better results compared to DQN, in line with previous studies proposing that algorithms optimizing a stochastic policy generally perform better than DQN [79,80], which is known to suffer more from low sample efficiency [81]. This can best be seen in the relative performance between different levels of information input. When a CNN was integrated into the RL models, some degree of learning (that did not reach statistical significance) was observed for these systems. Yet, BNN received only a fraction of the input information density compared to their RL opponents in this condition (8-pixel combination of rate coded and place coded stimulation compared to 40 × 40 pixels of the input image). A curse of dimensionality effect (where higher dimension input can require additional episodes to converge to a minima) that may be adversely impacting the RL agents under the Image Input condition was also considered as an explanation for the lower sample efficiency observed. To account for potential disadvantages occurring as a result of increased input dimensionality, we also examined 2 alternative designs for input structure to the RL algorithms (i.e., Paddle & Ball Position Input and Ball Position Input designs). In-depth comparison between BNN performance and these alternative RL algorithms did not provide any significantly different outcome in favor of the RL baselines’ sample efficiency (see Figs. 6 and 7). Indeed, poorer performance across these metrics for all RL algorithms was observed when input information became more sparse.

The fact that BNN could perform with such sparsely coded informational input and low sample sizes, conforms to coding mechanisms known to be used in biological intelligence [54,82,83]. While RL algorithms use back-propagation, it has been argued that this method is likely too inefficient to function within biological systems [18,25,28–30,84]. A more dynamic reconfiguration of network activity has been proposed to be necessary for the learning rates observed in biological cultures [18,29,30,85,86]. Theories of how this learning may occur include predictive coding, active inference, prospective configurations, and Hopfield networks, which have been used to describe how neural systems may reorganize activity for learning tasks [29,87–90]. While nuances among these different theories exist, the general notion supports the idea of a more biological consistent forward-based learning process compared to back-propagation. In support of this, our exploration of a biologically inspired algorithm with an active inference agent that uses counterfactual learning did find that improved learning rates were possible in silicon-based computing. While these results support the use of optimized learning strategies, the algorithms are highly dependent on hyper-parameter selection and require relatively higher power consumption compared to biological systems. Nonetheless, these results highlight the value of further exploring biologically inspired systems of learning and support the notion that SBI systems may offer a useful pathway to do this in the future.

Admittedly, a potential limitation of this work results from the fact that the space of hyper-parameters is too large for an exhaustive search in each algorithm. However, to explore a large number of hyper-parameters, we used values utilized in the original studies that introduced each algorithm. We tuned the hyper-parameters that were most sensitive by a grid search in a limited space of those parameters. As a result of their sensitivity to hyper-parameter selection, state-of-the-art deep RL algorithms remain challenging to apply. The use of model-based RL is proposed for achieving higher sample efficiencies. Model-free algorithms, however, often perform significantly better asymptotically than these algorithms [91]. Recently, different accelerated approaches have also been proposed for deep RL [81,91,92]. Nonetheless, many still lag behind the performance of the original algorithms or require modern computers and a combination of CPUs and GPUs prompting even higher computational costs [93]. As a future pathway, these modified algorithms may be utilized for further comparisons. Arguably, biological cultures operating with the DishBrain system do not require such fine-tuning of parameters or manipulation of the architecture.

It is important to note that this paper is not an isolated demonstration of relatively simple neural cultures engaging in non-trivial information processing. While previous studies employing open-loop, reservoir computing-style approaches have revealed the capacity of BNNs to perform complex non-linear transformations [47,48,75], these paradigms lack a direct, goal-oriented mechanism for shaping neural activity, as no feedback is provided. In such cases, improvements in classification performance typically reflect enhancements to the readout classifier, which may or may not coincide with underlying synaptic plasticity and a more stable pre-configured network response. In contrast, it is only under closed-loop conditions—such as those implemented in the present study—that one can meaningfully explore goal-directed adaptation and learning in BNNs. However, while a closed-loop paradigm is necessary to study learning, it is not sufficient on its own. Prior work has employed closed-loop protocols, but the tasks investigated were not easily comparable to those typically used in RL studies [44,45]. Despite this, the present study aligns with previous observations regarding the rapid reorganization of neural activity in BNNs in response to stimuli [39]. The observed changes in functional connectivity are also consistent with prior findings demonstrating that such metrics are readily detectable in in vitro neural cultures [48,52,53].

The key contribution of this work lies in its more rigorous methodology for generating representative mappings of functional connectivity across multiple distinct neural cultures. This approach is likely to be valuable for future studies, particularly as research continues to probe the relationship between structure and function in BNNs [94]. Furthermore, our study aligns with prior research suggesting that sparse, selective activation of neuronal populations plays a crucial role in learning and information processing [64,65]. By reducing network complexity while preserving essential dynamics, our approach provides a novel perspective on how in vitro networks encode and respond to structured inputs over time.

Beyond their advantages in sample efficiency and energy consumption, BNNs present intriguing possibilities for integration into engineering applications, particularly in adaptive control systems and real-time decision-making [39]. Their ability to self-organize, decode fuzzy inputs, and learn dynamically—yet robustly—with minimal input information suggests a potential for future bio-silico hybrid systems that leverage both biological intelligence and AI. However, noteable challenges remain, including maintaining long-term stability in cultured neural networks, improving the interfacing hardware to allow biological systems to interact better with silicon-based systems, and ensuring reproducibility across different biological preparations [94]. Addressing these challenges will be crucial for advancing BNN-based approaches in real-world applications, and future work should explore scalable methods for optimizing their use in engineering and computational systems [95]. While our findings provide compelling evidence of BNN learning capabilities, several limitations must be acknowledged. First, our study focused on a relatively short timescale of neural adaptation, and long-term learning dynamics in BNNs remain an open question. Future work should investigate whether these networks exhibit sustained plasticity over extended periods and whether their learning can generalize across different task conditions. Additionally, another important consideration is that our analysis was conducted in a highly controlled environment with structured input–output mappings, which may not fully capture the complexity of real-world learning. Future studies should explore more diverse task environments, adaptive feedback mechanisms, and richer sensory inputs to assess how BNNs scale to more complex problems.

Ultimately, the sample inefficiency of RL and high compute costs is well known, while biological systems are known to excel in both these areas. As such, while these results are novel and have not been empirically tested before, they should not be considered surprising. The key premise of this work was to empirically establish such a finding as a starting point to highlight the value of further exploring BNN based information processing systems along with more biologically inspired algorithms. Indeed, the findings presented here underscore the promise of biological intelligence systems for real-time learning, offering massive advantages in power efficiency and flexibility. A novel demonstration of how functional connectivity will rapidly change during learning tasks even for in vitro BNNs provides a foundation for future work to further explore. Moreover, evidence supporting that biological learning processes can meaningfully compete and even outperform RL algorithms in sample efficiency under real-world timescales should be considered further support for this future work to occur. Taken together, these results support that even rudimentary SBI systems with limited informational input are viable learning systems worth exploring. Coupled with the promise of considerable gains in power efficiency, flexibility of tasks, and upcoming improvements in associated technologies [76,96], these biologically based intelligence systems present a compelling pathway for realizing real-time, sample-efficient learning unachievable by current silicon-based approaches alone.

## Data Availability

All data generated for or used within this manuscript and all code for deep RL models or used for data analysis to generate the results in this manuscript have been deposited at Open Science Framework and are publicly available via https://osf.io/cnpzf/?view_only=a33b7083f78e4c55a20b6c021a695a4a. Any additional data supporting this study’s findings are available from the corresponding author upon reasonable request.

## Supplementary Materials

Supplementary 1 Supplementary Text Figs. S1 to S12 Tables S1 to S4 References [97–109]

## References

[1] Ramchandran K, Zeien E, Andreasen NC. Distributed neural efficiency: Intelligence and age modulate adaptive allocation of resources in the brain. Trends Neurosci Educ. 2019;15:48–61. 31176471 10.1016/j.tine.2019.02.006

[2] Kudithipudi D, Aguilar-Simon M, Babb J, Bazhenov M, Blackiston D, Bongard J, Brna AP, Raja SC, Cheney N, Clune J, et al. Biological underpinnings for lifelong learning machines. Nat Mach Intell. 2022;4(3):196–210.

[3] Lake BM, Ullman TD, Tenenbaum JB, Gershman SJ. Building machines that learn and think like people. Behav Brain Sci. 2017;40: Article e253. 27881212 10.1017/S0140525X16001837

[4] Hassabis D, Kumaran D, Summerfield C, Botvinick M. Neuroscience-inspired artificial intelligence. Neuron. 2017;95(2):245–258. 28728020 10.1016/j.neuron.2017.06.011

[5] Sutton RS, Barto AG. Reinforcement learning: An introduction. MIT Press; 2018.

[6] Hessel M, Modayil J, van Hasselt H, Schaul T, Ostrovski G, Dabney W, Horgan D, Piot B, Azar MG, Silver D. Rainbow: Combining improvements in deep reinforcement learning. arXiv. 2017. https://doi.org/10.48550/arXiv.1710.02298

[7] Moravčik M, Schmid M, Burch N, Lisy V, Morrill D, Bard N, Davis T, Waugh K, Johanson M, Bowling M. Deepstack: Expert-level artificial intelligence in heads-up no-limit poker. Science. 2017;356(6337):508–513. 28254783 10.1126/science.aam6960

[8] Jaderberg M, Czarnecki WM, Dunning I, Marris L, Lever G, Castaneda AG, Beattie C, Rabinowitz NC, Morcos AS, Ruderman A, et al. Human-level performance in first-person multiplayer games with population-based deep reinforcement learning. arXiv. 2018. 10.48550/arXiv.1807.01281 31147514

[9] Silver D, Hubert T, Schrittwieser J, Antonoglou I, Lai M, Guez A, Lanctot M, Sifre L, Kumaran D, Graepel T, et al. Mastering chess and shogi by self-play with a general reinforcement learning algorithm. arXiv. 2017. 10.48550/arXiv.1712.01815 30523106

[10] Silver D, Schrittwieser J, Simonyan K, Antonoglou I, Huang A, Guez A, Hubert T, Baker L, Lai M, Bolton A, et al. Mastering the game of go without human knowledge. Nature. 2017;550(7676):354–359. 29052630 10.1038/nature24270

[11] Silver D, Hubert T, Schrittwieser J, Antonoglou I, Lai M, Guez A, Lanctot M, Sifre L, Kumaran D, Graepel T, et al. A general reinforcement learning algorithm that masters chess, shogi, and go through self-play. Science. 2018;362(6419):1140–1144. 30523106 10.1126/science.aar6404

[12] Mnih V, Kavukcuoglu K, Silver D, Rusu AA, Veness J, Bellemare MG, Graves A, Riedmiller M, Fidjeland AK, Ostrovski G, et al. Human-level control through deep reinforcement learning. Nature. 2015;518(7540):529–533. 25719670 10.1038/nature14236

[13] Neftci EO, Averbeck BB. Reinforcement learning in artificial and biological systems. Nat Mach Intell. 2019;1(3):133–143.

[14] Richards BA, Lillicrap TP, Beaudoin P, Bengio Y, Bogacz R, Christensen A, Clopath C, Costa RP, de Berker A, Ganguli S, et al. A deep learning framework for neuroscience. Nat Neurosci. 2019;22(11):1761–1770. 31659335 10.1038/s41593-019-0520-2 PMC7115933

[15] Hasson U, Nastase SA, Goldstein A. Direct fit to nature: An evolutionary perspective on biological and artificial neural networks. Neuron. 2020;105(3):416–434. 32027833 10.1016/j.neuron.2019.12.002 PMC7096172

[16] Kagan BJ, Razi A, Bhat A, Kitchen AC, Tran NT, Habibollahi F, Khajehnejad M, Parker BJ, Rollo B, Friston KJ. Scientific communication and the semantics of sentience. Neuron. 2023;111(5):606–607. 36863320 10.1016/j.neuron.2023.02.008

[17] Kagan BJ, Mahlis M, Bhat A, Bongard J, Cole VM, Corlett P, Gyngell C, Hartung T, Jupp B, Levin M, et al. Toward a nomenclature consensus for diverse intelligent systems: Call for collaboration. Innovations. 2024;5(5): Article 100658. 10.1016/j.xinn.2024.100658 PMC11278797 39071220

[18] Tsividis PA, Pouncy T, Xu JL, Tenenbaum JB, Gershman SJ. Human learning in atari. AAAI Spring Symposium Series, Science of Intelligence: Computational Principles of Natural and Artificial Intelligence, Technical Report SS-17-07. Association for the Advancement of Artificial Intelligence; 2017.

[19] Marcus G. Deep learning: A critical appraisal. arXiv. 2018. https://doi.org/10.48550/arXiv.1801.00631

[20] Gibney E. This AI researcher is trying to ward off a reproducibility crisis. Nature. 2020;577(7788):14–14. 10.1038/d41586-019-03895-5 31871325

[21] Kirkpatrick J, Pascanu R, Rabinowitz N, Veness J, Desjardins G, Rusu AA, Milan K, Quan J, Ramalho T, Grabska-Barwinska A, et al. Overcoming catastrophic forgetting in neural networks. Proc Natl Acad Sci USA. 2017;114(13):3521–3526. 28292907 10.1073/pnas.1611835114 PMC5380101

[22] Fan L, Glynn PW. The fragility of optimized bandit algorithms. 2022. https://doi.org/10.1287/opre.2023.0283

[23] Mousavi SS, Schukat M, Howley E. Deep reinforcement learning: An overview. In: Bi Y, Kapoor S, Bhatia R, editors. *Proceedings of SAI Intelligent Systems Conference (IntelliSys) 2016*. Cham: Springer International Publishing; 2018. p. 426–440.

[24] Freitag C, Berners-Lee M, Widdicks K, Knowles B, Blair GS, Friday A. The real climate and transformative impact of ICT: A critique of estimates, trends, and regulations. Patterns. 2021;2(9): Article 100340. 34553177 10.1016/j.patter.2021.100340 PMC8441580

[25] Whittington JCR, Bogacz R. Theories of error back-propagation in the brain. Trends Cogn Sci. 2019;23(3):235–250. 30704969 10.1016/j.tics.2018.12.005 PMC6382460

[26] Hamid AA, Pettibone JR, Mabrouk OS, Hetrick VL, Schmidt R, Vander Weele CM, Kennedy RT, BJ, JD. Mesolimbic dopamine signals the value of work. Nat Neurosci. 2016;19(1):117–126. 26595651 10.1038/nn.4173 PMC4696912

[27] Costa VD, Dal Monte O, Lucas DR, Murray EA, Averbeck BB. Amygdala and ventral striatum make distinct contributions to reinforcement learning. Neuron. 2016;92(2):505–517. 27720488 10.1016/j.neuron.2016.09.025 PMC5074688

[28] Friston KJ, Daunizeau J, Kiebel SJ. Reinforcement learning or active inference? PLOS ONE. 2009;4(7): Article e6421. 19641614 10.1371/journal.pone.0006421 PMC2713351

[29] Song Y, Millidge B, Salvatori T, Lukasiewicz T, Xu Z, Bogacz R. Inferring neural activity before plasticity: A foundation for learning beyond backpropagation. Nat Neurosci. 2022;27(2):348–358. 10.1038/s41593-023-01514-1 PMC7615830 38172438

[30] Whittington JCR, Bogacz R. An approximation of the error backpropagation algorithm in a predictive coding network with local Hebbian synaptic plasticity. Neural Comput. 2017;29(5):1229–1262. 28333583 10.1162/NECO_a_00949 PMC5467749

[31] Pang JC, Aquino KM, Oldehinkel M, Robinson PA, Fulcher BD, Breakspear M, Fornito A. Geometric constraints on human brain function. Nature. 2023;618(7965):566–574. 37258669 10.1038/s41586-023-06098-1 PMC10266981

[32] Damoiseaux JS, Greicius MD. Greater than the sum of its parts: A review of studies combining structural connectivity and resting-state functional connectivity. Brain Struct Funct. 2009;213:525–533. 19565262 10.1007/s00429-009-0208-6

[33] Sadaghiani S, Poline J-B, Kleinschmidt A, D’Esposito M. Ongoing dynamics in large-scale functional connectivity predict perception. Proc Natl Acad Sci USA. 2015;112(27):8463–8468. 26106164 10.1073/pnas.1420687112 PMC4500238

[34] Kagan BJ, Alon Loeffler J, Boyd L, Savulescu J. Embodied neural systems can enable iterative investigations of morally relevant states. J Neurosci. 2024;44(15): Article e0431242024. 38599798 10.1523/JNEUROSCI.0431-24.2024 PMC11007307

[35] Saxena S, Cunningham JP. Towards the neural population doctrine. Curr Opin Neurobiol. 2019;55:103–111. 30877963 10.1016/j.conb.2019.02.002

[36] Mediano PAM, Rosas FE, Luppi AI, Jensen HJ, Seth AK, Barrett AB, Carhart-Harris RL, Bor D. Greater than the parts: A review of the information decomposition approach to causal emergence. Phil Trans R Soc A. 2022;380(2227): Article 20210246. 35599558 10.1098/rsta.2021.0246 PMC9125226

[37] Rasmussen RG, Schwartz A, Chase SM. Dynamic range adaptation in primary motor cortical populations. eLife. 2017;6: Article e21409. 28417848 10.7554/eLife.21409 PMC5395298

[38] Hagos DH, Battle R, Rawat DB. Recent advances in generative AI and large language models: Current status, challenges, and perspectives. IEEE Trans Artif Intell. 2024;5(12):5873–5893.

[39] Kagan BJ, Habibollahi F, Watmuff B, Azadi A, Doensen F, Loeffler A, Byun SH, Servais B, Desouza C, Abu-Bonsrah KD, Kerlero de Rosbo N. Harnessing Intelligence from Brain Cells In Vitro. Neuroscientist. 2025; 10.1177/10738584251321438 PMC12426333 40079153

[40] Kagan BJ, Gyngell C, Lysaght T, Cole VM, Sawai T, Savulescu J. The technology, opportunities and challenges of synthetic biological intelligence. Biotechnol Adv. 2023;68:108233. 37558186 10.1016/j.biotechadv.2023.108233 PMC7615149

[41] Jimbo Y, Robinson HPC, Kawana A. Strengthening of synchronized activity by tetanic stimulation in cortical cultures: Application of planar electrode arrays. IEEE Trans Biomed Eng. 1998;45(11):1297–1304. 9805828 10.1109/10.725326

[42] Shahaf G, Marom S. Learning in networks of cortical neurons. J Neurosci. 2001;21(22):8782–8788. 11698590 10.1523/JNEUROSCI.21-22-08782.2001 PMC6762268

[43] Pasquale V, Massobrio P, Bologna LL, Chiappalone M, Martinoia S. Self-organization and neuronal avalanches in networks of dissociated cortical neurons. Neuroscience. 2008;153(4):1354–1369. 18448256 10.1016/j.neuroscience.2008.03.050

[44] Bakkum DJ, Chao ZC, Potter SM. Spatio-temporal electrical stimuli shape behavior of an embodied cortical network in a goal-directed learning task. J Neural Eng. 2008;5(3):310–323. 18714127 10.1088/1741-2560/5/3/004 PMC2559979

[45] Tessadori J, Bisio M, Martinoia S, Chiappalone M. Modular neuronal assemblies embodied in a closed-loop environment: Toward future integration of brains and machines. Front Neural Circuits. 2012;6:99. 23248586 10.3389/fncir.2012.00099 PMC3520178

[46] Müller J, Bakkum DJ, Hierlemann A. Sub-millisecond closed-loop feedback stimulation between arbitrary sets of individual neurons. Front Neural Circuits. 2013;6:121. 23335887 10.3389/fncir.2012.00121 PMC3541546

[47] Isomura T, Kotani K, Jimbo Y. Cultured cortical neurons can perform blind source separation according to the free-energy principle. PLOS Comput Biol. 2015;11: Article e1004643. 26690814 10.1371/journal.pcbi.1004643 PMC4686348

[48] Cai H, Ao Z, Tian C, Zhuhao W, Liu H, Tchieu J, Mingxia G, Mackie K, Guo F. Brain organoid reservoir computing for artificial intelligence. Nat Electron. 2023;6(12):1032–1039.

[49] Kagan BJ, Kitchen AC, Tran NT, Habibollahi F, Khajehnejad M, Parker BJ, Bhat A, Rollo B, Razi A, Friston KJ. In vitro neurons learn and exhibit sentience when embodied in a simulated game-world. Neuron. 2022;110(23):3952–3969. 36228614 10.1016/j.neuron.2022.09.001 PMC9747182

[50] Habibollahi F, Kagan BJ, Burkitt AN, French C. Critical dynamics arise during structured information presentation within embodied in vitro neuronal networks. Nat Commun. 2023;14(1):5287. 37648737 10.1038/s41467-023-41020-3 PMC10469171

[51] Cavallo A, Köhler RM, Busch JL, Habets JGV, Merk T, Zvarova P, Vanhoecke J, Binns TS, Al-Fatly B, de Almeida Marcelino AL, et al. Differential modulation of movement speed with state-dependent deep brain stimulation in parkinson’s disease. bioRxiv. 2025. https://www.biorxiv.org/content/10.1101/2025.03.19.642627v1 10.1126/sciadv.adx6849 PMC12422180 40929271

[52] Parodi G, Zanini G, Chiappalone M, Martinoia S. Electrical and chemical modulation of homogeneous and heterogeneous human-iPSCs-derived neuronal networks on high density arrays. Front Mol Neurosci. 2024;17: Article 1304507. 38380114 10.3389/fnmol.2024.1304507 PMC10877635

[53] Poli D, Pastore VP, Massobrio P. Functional connectivity in in vitro neuronal assemblies. Front Neural Circuits. 2015;9:57. 26500505 10.3389/fncir.2015.00057 PMC4595785

[54] Harrell ER, Goldin MA, Bathellier B, Shulz DE. An elaborate sweep-stick code in rat barrel cortex. Sci Adv. 2020;6(38): Article eabb7189. 32938665 10.1126/sciadv.abb7189 PMC7494352

[55] Ruaro ME, Bonifazi P, Torre V. Toward the neurocomputer: Image processing and pattern recognition with neuronal cultures. IEEE Trans Biomed Eng. 2005;52(3):371–383. 15759567 10.1109/TBME.2004.842975

[56] Perozzi B, Al-Rfou R, Skiena S. Deepwalk: Online learning of social representations. In: *Proceedings of the 20th ACM SIGKDD International Conference on Knowledge Discovery and Data Mining*. 2014. p. 701–710.

[57] Tang J, Qu M, Wang M, Zhang M, Yan J, Mei Q. Line: Large-scale information network embedding. In: *Proceedings of the 24th International Conference on World Wide Web*. 2015. p. 1067–1077.

[58] Khajehnejad M. Simnet: Similarity-based network embeddings with mean commute time. PLOS ONE. 2019;14(8): Article e0221172. 31415635 10.1371/journal.pone.0221172 PMC6695167

[59] Van der Maaten L, Hinton G. Visualizing data using t-SNE. J Mach Learn Res. 2008;9(86):2579–2607.

[60] Renart A, Brunel N, Wang X-J. Mean-field theory of irregularly spiking neuronal populations and working memory in recurrent cortical networks. In: *Computational neuroscience: A comprehensive approach*. Boca Raton (FL): Chapman & Hall/CRC; 2004. p. 431–490.

[61] Baspinar E, Schülen L, Olmi S, Zakharova A. Coherence resonance in neuronal populations: Mean-field versus network model. Phys Rev E. 2021;103(3): Article 032308. 33862689 10.1103/PhysRevE.103.032308

[62] Bick C, Goodfellow M, Laing CR, Martens EA. Understanding the dynamics of biological and neural oscillator networks through exact mean-field reductions: A review. *The journal of mathematical*. Neuroscience. 2020;10(1):9. 10.1186/s13408-020-00086-9 PMC7253574 32462281

[63] La Camera G. The mean field approach for populations of spiking neurons. In: Computational modelling of the brain: Modelling approaches to cells, circuits and networks. Springer; 2021. p. 125–157. 10.1007/978-3-030-89439-9_6 PMC10317473 35471538

[64] Wolfe J, Houweling AR, Brecht M. Sparse and powerful cortical spikes. Curr Opin Neurobiol. 2010;20(3):306–312. 20400290 10.1016/j.conb.2010.03.006

[65] Olshausen BA, Field DJ. Sparse coding of sensory inputs. Curr Opin Neurobiol. 2004;14(4):481–487. 15321069 10.1016/j.conb.2004.07.007

[66] Arulkumaran K, Deisenroth MP, Brundage M, Bharath AA. Deep reinforcement learning: A brief survey. IEEE Signal Process Mag. 2017;34(6):26–38.

[67] Schulman J, Wolski F, Dhariwal P, Radford A, Klimov O. Proximal policy optimization algorithms. arXiv. 2017. 10.48550/arXiv.1707.06347

[68] Bellman R, Kalaba R. Dynamic programming and statistical communication theory. Proc Natl Acad Sci USA. 1957;43(8):749–751. 16590080 10.1073/pnas.43.8.749 PMC528532

[69] Tenenbaum JB, de Silva V, Langford JC. A global geometric framework for nonlinear dimensionality reduction. Science. 2000;290(5500):2319–2323. 11125149 10.1126/science.290.5500.2319

[70] Khajehnejad M, Habibollahi F, Loeffler A, Kagan B, Razi A. On complex network dynamics of an in-vitro neuronal system during rest and gameplay. In: *NeurIPS 2023 Generative AI and Biology (GenBio) Workshop*. New Orleans (LA): NeurIPS; 2023.

[71] Eugene Stanley H. Phase transitions and critical phenomena. Oxford: Clarendon Press; 1971. Vol. 7.

[72] Barrat A, Barthelemy M, Vespignani A. Dynamical processes on complex networks. Cambridge Univ. Press; 2008.

[73] Papadimitriou CH, Vempala SS, Mitropolsky D, Collins M, Maass W. Brain computation by assemblies of neurons. Proc Natl Acad Sci USA. 2020;117(25):14464–14472. 32518114 10.1073/pnas.2001893117 PMC7322080

[74] Luo L. Architectures of neuronal circuits. Science. 2021;373(6559): Article eabg7285. 34516844 10.1126/science.abg7285 PMC8916593

[75] Sumi T, Yamamoto H, Katori Y, Ito K, Moriya S, Konno T, Sato S, Hirano-Iwata A. Biological neurons act as generalization filters in reservoir computing. Proc Natl Acad Sci USA. 2023;120(25): Article e2217008120. 37307467 10.1073/pnas.2217008120 PMC10288593

[76] Kagan, B. J. The CL1 as a platform technology for leveraging biological neural system functions. Nat. Rev. Bioeng. (2025). 10.1038/s44222-025-00340-3

[77] Girardin S, Ihle SJ, Menghini A, Krubner M, Tognola L, Duru J, Fruh I, Müller M, Ruff T, Vörös J. Engineering circuits of human iPSC-derived neurons and rat primary glia. Front Neurosci. 2023;17:1103437. 37250404 10.3389/fnins.2023.1103437 PMC10213452

[78] Jouppi NP, Yoon DH, Kurian G, Li S, Patil N, Laudon J, Young C, Patterson D. A domain-specific supercomputer for training deep neural networks. Commun ACM. 2020;63(7):67–78.

[79] Siddique U, Weng P, Zimmer M. Learning fair policies in multi-objective (deep) reinforcement learning with average and discounted rewards. In: *International Conference on Machine Learning*. PMLR; 2020. p. 8905–8915.

[80] El Mazgualdi C, Masrour T, Barka N, El Hassani I. A learning-based decision tool towards smart energy optimization in the manufacturing process. Systems. 2022;10(5):180.

[81] Lee SY, Sungik C, Chung S-Y. Sample-efficient deep reinforcement learning via episodic backward update. Adv Neural Inf Proces Syst. 2019;32:2112–2121.

[82] Buchanan M. Organoids of intelligence [thesis]. Nature Publishing Group; 2018.

[83] Bastos AM, Vezoli J, Bosman CA, Schoffelen J-M, Oostenveld R, Dowdall JR, De Weerd P, Kennedy H, Fries P. Visual areas exert feedforward and feedback influences through distinct frequency channels. Neuron. 2015;85(2):390–401. 25556836 10.1016/j.neuron.2014.12.018

[84] Geoffrey Hinton. The forward-forward algorithm: Some preliminary investigations. arXiv. 2022. 10.48550/arXiv.2212.13345

[85] Felleman DJ, Van Essen DC. Distributed hierarchical processing in the primate cerebral cortex. Cereb Cortex. 1991;1(1):1–47. 1822724 10.1093/cercor/1.1.1-a

[86] Friston KJ, Salvatori T, Isomura T, Tschantz A, Kiefer A, Verbelen T, Koudahl M, Paul A, Parr T, Razi A, et al. Active inference and intentional behavior. Neural Comput. 2025;74(4):666–700. 10.1162/neco_a_01738 40030135

[87] Hopfield JJ. Neural networks and physical systems with emergent collective computational abilities. Proc Natl Acad Sci USA. 1982;79(8):2554–2558. 6953413 10.1073/pnas.79.8.2554 PMC346238

[88] Rao RPN, Ballard DH. Predictive coding in the visual cortex: A functional interpretation of some extra-classical receptive-field effects. Nat Neurosci. 1999;2(1):79–87. 10195184 10.1038/4580

[89] Friston K. A theory of cortical responses. Philos Trans R Soc B Biol Sci. 2005;360(1456):815–836. 10.1098/rstb.2005.1622 PMC1569488 15937014

[90] de Wit L, Machilsen B, Putzeys T. Predictive coding and the neural response to predictable stimuli. J Neurosci. 2010;30(26):8702–8703. 20592191 10.1523/JNEUROSCI.2248-10.2010 PMC6632880

[91] Chua K, Calandra R, McAllister R, Levine S. Deep reinforcement learning in a handful of trials using probabilistic dynamics models. In: *Advances in Neural Information Processing Systems 31 (NeurIPS 2018)*. Montreal (Canada): NeurIPS; 2018.

[92] Franke JKH, Köhler G, Biedenkapp A, Hutter F. Sample-efficient automated deep reinforcement learning. arXiv. 2020. 10.48550/arXiv.2009.01555

[93] Stooke A, Abbeel P. Accelerated methods for deep reinforcement learning. arXiv. 2018. 10.48550/arXiv.1803.02811

[94] Tanveer MS, Patel D, Schweiger HE, Abu-Bonsrah KD, Watmuff B, Azadi A, Pryshchep S, Narayanan K, Puleo C, Natarajan K, Mostajo-Radji MA, Kagan BJ, Wang G. Starting a synthetic biological intelligence lab from scratch. Patterns. 2025;6(5):101232. 40486968 10.1016/j.patter.2025.101232 PMC12142634

[95] Kagan BJ, Habibollahi F, Watmuff B, Azadi A, Doensen F, Loeffler A, Byun SH, Servais B, Desouza C, Abu-Bonsrah KD, et al. Harnessing intelligence from brain cells in vitro. Neuroscientist 10.1177/10738584251321438. 2025. 10.1177/10738584251321438 PMC12426333 40079153

[96] Smirnova L, Caffo BS, Gracias DH, Huang Q, Morales IE, Pantoja BT, Zack DJ, Berlinicke CA, Lomax Boyd J, Harris TD, et al. Organoid intelligence (OI): The new frontier in biocomputing and intelligence-in-a-dish. Front Sci. 2023;1:1017235.

[97] Keskar NS, Mudigere D, Nocedal J, Smelyanskiy M, Tang PTP. On large-batch training for deep learning: Generalization gap and sharp minima. arXiv. 2016. 10.48550/arXiv.1609.04836

[98] Isomura T, Friston K. Reverse-engineering neural networks to characterize their cost functions. Neural Comput. 2020;32(11):2085–2121. 32946704 10.1162/neco_a_01315

[99] Isomura T, Shimazaki H, Friston KJ. Canonical neural networks perform active inference. Commun Biol. 2022;5(1):55. 35031656 10.1038/s42003-021-02994-2 PMC8760273

[100] Friston K, Da Costa L, Hafner D, Hesp C, Parr T. Sophisticated inference. Neural Comput. 2021;33(3):713–763. 33626312 10.1162/neco_a_01351

[101] Kaplan R, Friston KJ. Planning and navigation as active inference. Biol Cybern. 2018;112(4):323–343. 29572721 10.1007/s00422-018-0753-2 PMC6060791

[102] Kuchling F, Friston K, Georgiev G, Levin M. Morphogenesis as Bayesian inference: A variational approach to pattern formation and control in complex biological systems. Phys Life Rev. 2020;33:88–108. 31320316 10.1016/j.plrev.2019.06.001

[103] Tschantz A, Seth AK, Buckley CL. Learning action-oriented models through active inference. PLOS Comput Biol. 2020;16(4):1–30. 10.1371/journal.pcbi.1007805 PMC7200021 32324758

[104] Parr T, Friston KJ. The discrete and continuous brain: From decisions to movement-and back again. Neural Comput. 2018;30(29894658):2319–2347. 29894658 10.1162/neco_a_01102 PMC6115199

[105] Isomura T. Active inference leads to Bayesian neurophysiology. Neurosci Res. 2022;175:38–45. 34968557 10.1016/j.neures.2021.12.003

[106] Lovejoy WS. A survey of algorithmic methods for partially observed Markov decision processes. Ann Oper Res. 1991;28(1):47–65.

[107] Shani G, Pineau J, Kaplow R. A survey of point-based POMDP solvers. Auton Agent Multi-Agent Syst. 2013;27(1):1–51.

[108] Kaelbling LP, Littman ML, Cassandra AR. Planning and acting in partially observable stochastic domains. Artif Intell. 1998;101(1):99–134.

[109] Paul A, Sajid N, Gopalkrishnan M, Razi A. Active inference for stochastic control. In: *Joint European Conference on Machine Learning and Knowledge Discovery in Databases*. Springer; 2021. p. 669–680.

