# Supplementary material for: Dynamic Network Plasticity and Sample Efficiency in Biological Neural Cultures: A Comparative Study with Deep Reinforcement Learning
Source: Cyborg Bionic Syst. 2025 Aug 4;6:0336. doi: 10.34133/cbsystems.0336 (PMC12320521; doi:10.34133/cbsystems.0336)
Supplement: Supplementary 1 — Supplementary Text Figs. S1 to S12 Tables S1 to S4 References [97–109] [file cbsystems.0336.f1.pdf]

A Supplementary Materials

A.1 Cell Culture

Neural cells were cultured either from the cortices of E15.5 mouse embryos or differentiated from human induced pluripotent stem cells via a dual SMAD inhibition (DSI) protocol as previously described [48]. Cells were cultured until plating onto MEA. For primary mouse neurons, this occurred at day-in-vitro (DIV) 0, for DSI cultures this occurred at between DIV 30 - 33 depending on culture development.

A.2 MEA Setup and Plating

MaxOne Multielectrode Arrays (MEA; Maxwell Biosystems, AG, Switzerland) was used and is a high-resolution electrophysiology platform featuring 26,000 platinum electrodes arranged over an 8 mm2. The MaxOne system is based on complementary meta-oxide-semiconductor (CMOS) technology and allows recording from up to 1024 channels. MEAs were coated with either polyethylenimine (PEI) in borate buffer for primary culture cells or Poly-D-Lysine for cells from an iPSC background before being coated with either 10 µg/ml mouse laminin or 10 µg/ml human 521 Laminin (Stemcell Technologies Australia, Melbourne, Australia) respectively to facilitate cell adhesion. Approximately 10^6 cells were plated on MEA after preparation as per [48]. Cells were allowed approximately one hour to adhere to the MEA surface before the well was flooded. The day after plating, cell culture media was changed for all culture types to BrainPhys™ Neuronal Medium (Stemcell Technologies Australia, Melbourne, Australia) supplemented with 1% penicillin-streptomycin. Cultures were maintained in a low O2 incubator kept at 5% CO2, 5% O2, 36°C and 80% relative humidity. Every two days, half the media from each well was removed and replaced with free media. Media changes always occurred after all recording sessions.

A.3 *DishBrain* platform and electrode configuration

The current *DishBrain* platform is configured as a low-latency, real-time MEA control system with on-line spike detection and recording software. The *DishBrain* platform provides on-line spike detection and recording configured as a low-latency, real-time MEA control. The *DishBrain* software runs at 20 kHz and allows recording at an incredibly fine timescale. There is the option of recording spikes in binary files, and regardless of recording, they are counted over a period of 10 milliseconds (200 samples), at which point the game environment is provided with how many spikes are detected in each electrode in each predefined motor region as described below. Based on which motor region the spikes occurred in, they are interpreted as motor activity, moving the ‘paddle’ up or down in the virtual space. As the ball moves around the play area at a fixed speed and bounces off the edge of the play area and the paddle, the pong game is also updated at every 10ms interval. Once the ball hits the edge of the play area behind the paddle, one rally of pong has come to an end at which point a ’miss’ would be recorded and an unpredictable stimulation would be delivered to the cells. Using a feedback stimulus at a voltage of 150 mV and a frequency of 5 Hz, unpredictable external stimulus could be added to the system. Random stimulation took place at random sites over the 8 predefined input electrodes at random timescales for a period of four seconds, followed by a configurable rest period of four seconds where stimulation paused, then the next rally began.

In contrast, a predictable stimulus feedback is provided when the ball contacts the paddle under the standard stimulus condition. Predictable stimulus feedback involves 75mV stimulation at 100Hz over 100ms occurring when the simulated ball struck the paddle and replaced other sensory information. All 8 stimulation electrodes simultaneously would receive predictable stimulation at this frequency and period. A ‘stimulation sequencer’ module tracks the location of the ball relative to the paddle during each rally and encodes it as stimulation to one of eight stimulation sites. Each time a sample is received from the MEA, the stimulation sequencer is updated 20,000 times a second, while the game itself runs at 100Hz. After the previous lot of MEA commands has completed, the *DishBrain* system constructs a

new sequence of MEA commands based on the information it has been configured to transmit based on both place codes and rate codes. The stimulations take the form of a short square bi-phasic pulse that is a positive voltage, then a negative voltage. This pulse sequence is read and applied to the electrode by a Digital to Analog Converter (or DAC) on the MEA. A real-time interactive version of the game visualizer is available at `https://spikestream.corticallabs.com/`. Alternatively, cells could be recorded at ‘rest’ in a gameplay environment where activity was recorded to move the paddle but no stimulation was delivered, with corresponding outcomes still recorded. Using this spontaneous activity alone as a baseline, the gameplay characteristics of a culture were determined. Low level code for interacting with Maxwell API was written in C to minimize processing latencies-so packet processing latency was typically $<50\ \mu$s. High-level code was written in Python, including configuration setups and general instructions for game settings. A 5 ms spike-to-stim latency was achieved, which was substantially due to MaxOne’s inbuilt hardware buffering. Figure S1 illustrates a schematic view of Software components and data flow in the *DishBrain* closed loop system.

A.4 Region-Based Functional Connectivity Analysis

To examine whether spatially organized functional pathways emerge during *Gameplay*, we conducted a complementary analysis based on anatomical grouping of electrodes. Specifically, we divided the sensory and motor regions into spatially defined subregions to evaluate structured connectivity between areas aligned with sensory input and motor output.

The sensory area of the dish contains hundreds of electrodes. For encoding the y-position of the ball, this region is evenly divided into 8 horizontal segments. From each of these 8 segments, one electrode is selected and designated as a stimulation electrode. At every time step, the segment corresponding to the ball’s current y-position is stimulated. The frequency of stimulation to this electrode is further modulated by the ball’s x-position, i.e., its distance from the paddle. Thus, the sensory encoding scheme does not implement a binary ”sensory up/sensory down” configuration, but rather a more spatially distributed and continuous representation along the y-axis. We have nonetheless implemented two analyses to explore how sensorimotor connectivity evolves:

- **10-region scheme (8 sensory + 2 motor)**: We grouped the recording electrodes into 10 spatial regions—8 segments covering the full y-axis of the sensory area (aligned with the 8 stimulation zones), and 2 motor regions (Up and Down). We then computed pairwise functional connectivity (Pearson correlation) between these groups.
- **4-region scheme (2 sensory + 2 motor)**: Next, to investigate a simplified setting, we selected only the subset of sensory electrodes that directly face the motor regions (i.e., share the same y-axis boundaries). We then split these into upper and lower halves, resulting in 2 ”aligned” sensory regions (facing motor-up and motor-down, respectively), along with the 2 motor regions.

Figure S2 represents the division of electrodes into the above-mentioned subregions.

For both grouping schemes, we aggregated spiking activity among all electrodes of that region, computed and visualized the functional connectivity matrices across time using spiking activity, comparing the early and late phases (first and last 2 minutes) of *Gameplay* and *Rest*. These analyses provide insight into how inter-region connectivity evolves during *Gameplay* as opposed to *Rest* sessions, and whether task engagement induces distinct functional coupling patterns.

Figures S3 and S4 represents the connectivity matrices for the first and last 2 minutes of *Gameplay* vs. *Rest* when dividing the electrode maps into 10 and 4 regions, respectively.

We note from the connectivity matrices from the spatially grouped regions that while the weights are significantly different between *Gameplay* and *Rest*, the weights within these groups are spatially relatively homogeneous for each sub-group. This uniformity stems from the fact that the spatial grouping method includes all electrodes within each subregion, regardless of their actual level of neural engagement. Given

the biological variability across cultures, particularly in terms of where neurons adhere and which neurons become functionally active, over a large enough sample this variability should be expected (and our data shows this) to dilute any spatially distinct functional specificity. This reinforces our original motivation for employing an unsupervised method to identify representative channels, which adapts to the unique activity profile of each culture, allowing us to capture meaningful temporal changes and specialization that are otherwise obscured.

Overall, these findings can reveal that during *Gameplay*, the average inter-region correlations between these functional groups decrease over time. This suggests a trend toward functional specialization, where distinct subregions become less globally synchronized and potentially more dedicated to their respective sensory or motor roles. In contrast, during *Rest*, no such temporal reorganization was observed, and correlation levels remained relatively stable or more diffuse across all regions. We note that the results from this region-based analysis offer a complementary perspective to our initial findings using 30 representative channels selected through an unsupervised method. In that analysis (see Figure 4), we observed an increase in pairwise correlations, a reduction in modularity, and an increase in average clustering coefficient over time. Rather than suggesting a discrepancy, these outcomes likely reflect distinct levels of network organization being captured by the two approaches. The unsupervised method focuses on a dynamic subset of the most functionally engaged channels, revealing micro-scale coordination and local circuit integration. In contrast, the region-based method aggregates activity over broader anatomical areas, highlighting macro-scale reconfiguration and global specialization during learning.

The unsupervised method selectively captures the most functionally active and tightly coupled microcircuits, which appear to undergo local integration and strengthening as learning progresses. These represent task-engaged "core" subnetworks, and their increasing connectivity aligns with our observations of emergent coordination and network consolidation. The structured region-based approach, on the other hand, includes all electrodes within each subregion, regardless of activity level. As such, it is sensitive to broader changes in inter-region coordination and may be more affected by heterogeneity or divergence in activity across the included channels. The observed decrease in correlation may reflect a reduction in global co-fluctuation as the network reorganizes toward more specialized and modular sub-populations, a hallmark of maturing task-relevant functional architecture.

Taken together, these complementary analyses reinforce a coherent narrative: during learning, our system exhibits both local functional integration within active subcircuits and global functional segregation across anatomical regions. This pattern is consistent with theories of network reconfiguration seen in biological learning systems, where core modules consolidate while global architecture becomes more specialized.

A.5 Deep Reinforcement Learning Algorithms

Deep Q Network (DQN): The utilized DQN algorithm begins by extracting spatiotemporal features from inputs, such as the movement of the ball in the game of 'Pong'. Multiple fully connected layers are used to process the final feature map, which implicitly encodes the effects of actions. As opposed to traditional controllers that use fixed preprocessing steps, this method can adapt the processing of the state based on changes in the learning signal. An epsilon-greedy algorithm was employed in this work to balance the exploration and exploitation capabilities of the DQN algorithm.

For the results represented in this manuscript, a comprehensive grid search was conducted within the parameter space of *learning rate* ($[0.0001, 0.004]$), *replay buffer size* ($[10, 100000]$), and the training *batch size* ($[5, 128]$) with starting point of $0.0001, 32, 10000$, respectively, aiming to identify the optimal parameter configuration. The results presented in this paper are derived from the superior set of hyper-parameters obtained through this search process. As the outcome of this search for the DQN algorithm, we selected *learning rate = 0.002*, *replay buffer size = 10000*, and *batch size = 16* for the results of Figure 5, *learning rate = 0.001*, *replay buffer size = 10000*, and *batch size = 16* for the results of Figure 6, and

learning rate = 0.001, *replay buffer size = 10000*, and *batch size = 32* for the results of Figure 7. Figure S6 illustrates the performance of the DQN algorithm with Image Input design in terms of average rally length in several sample points of the mentioned search space. While exploring each hyper-parameter in Figure S6, the remaining pair are set to the same values as the starting point of the search (i.e. *learning rate = 0.0001*, *batch size = 32*, and *replay buffer size = 10000*).

For additional details on the set of explored hyper-parameters and network architectures, see Table S1.

Algorithm 1 Deep Q Network (DQN) with Experience Replay

```
Require:
𝒟: Replay buffer with size N (Default: 10000)
θ: Initial network parameters
θ̃: Copy of θ
γ: Discount factor (Default: 0.95)
N_b: Training batch size (Default: 16)
Ñ: Target network update frequency (Default: 10)
x_t: Input matrix at time t
r_t: 1 if hit in t, 0 otherwise
S: Number of seeds (Default: 150)
e_max: Maximum number of episodes (Default: 70)
for seed ∈ {1, ··· , S} do
    for episode e ∈ {1, ··· , e_max} do
        Set state s_1 ← x_1 and preprocess φ_1 = φ(s_1)
        t = 1
        while φ_t is non-terminal do
            With probability ϵ select a random action a_t
            otherwise select a_t = max_a Q*(φ(s_t), a; θ)
            Execute action a_t and observe reward r_t and input x_{t+1}
            Set new state s_{t+1} and preprocess φ_{t+1} = φ(s_{t+1})
            Store transition (φ_t, a_t, r_t, φ_{t+1}) in 𝒟
            Sample random minibatch of N_b transitions(φ_j, a_j, r_j, φ_{j+1}) from 𝒟
            Set y_j = { r_j                                  terminal φ_{j+1}
                      { r_j + γ max_{a'} Q(φ_{j+1}, a'; θ)   non-terminal φ_{j+1}
            Perform a gradient descent step on (y_j − Q(φ_j, a_j; θ))^2
            Replace target parameters θ̃ ← θ every Ñ steps
            t = t + 1
        end while
    end for
end for
```

Advantage Actor-Critic (A2C): In an A2C model, the total reward itself could be represented as a *value* of the state plus the advantage of the action. The *value* of each policy is learned while following it. The policy gradient can be calculated by knowing the *value* for any state. The policy network is then updated such that the probability of actions with a higher advantage value is increased. Here, the policy network (which returns a probability distribution of actions) is called the *actor*, as it tells the agents what to do. *Critic* is another network that enables the evaluation of the actions to decide whether they were good or not. In this case, policy and value are implemented as separate heads of the network, which transform the output from the common body into either probability distributions or single numbers representing the state's value. Thus, low-level features can be shared between the two networks.

For the results represented in the main paper, a comprehensive grid search was conducted within the parameter space of actor learning rate ($[0.0001, 0.004]$), critic learning rate ($[0.0001, 0.004]$), and the training batch size ($[5, 128]$), to identify the optimal parameter configuration. As the outcome of this search for A2C, we selected *actor learning rate = 0.001, 0.0001, 0.003*, *critic learning rate = 0.001, 0.001 , 0.001*, and *batch size = 32, 32,5* for the results of Figure 5, 6, and 7, respectively. Figure S6 contains the results of this hyper-parameter search for the A2C algorithm with the Image Input design in terms of average rally length in several sample points of the mentioned search space. While exploring each hyper-parameter in Figure S6, the remaining pair are set to the same values as the starting point

of the search (i.e. *actor learning rate = 0.0001*, *batch size = 32*, and *critic learning rate = 0.001*).

Algorithm 2 Advantage Actor-Critic (A2C)

Require:
θ_v: Initial parameter vector for the value net (critic)
θ_π: Initial parameter vector for the policy net (actor)
γ: Discount factor (Default: 0.95)
N: Number of consecutive steps to play current policy in the environment (Default: 5)
r_t: 1 if hit in t, 0 otherwise
x_t: Input matrix at time t
S: Number of seeds (Default: 150)
e_{max}: Maximum number of episodes (Default: 70)
for seed $\in \{1, \cdots, S\}$ **do**
  $t = 1$
  $e = 1$
  repeat
    $\partial\theta_\pi \leftarrow 0$ and $\partial\theta_v \leftarrow 0$
    $t_{start} = t$
    Set state $s_t \leftarrow x_t$ and preprocess $\phi_t = \phi(s_t)$
    repeat
      Select a_t according to $\pi(a_t \mid \phi_t; \theta)$
      Execute action a_t and observe reward r_t and input x_{t+1}
      Set new state s_{t+1} and preprocess $\phi_{t+1} = \phi(s_{t+1})$
      $t \leftarrow t + 1$
    until ϕ_t is terminal **or** $t - t_{start} = N$
    $R = \begin{cases} 0 & \text{for terminal } \phi_t \\ V(\phi_t; \theta_v) & \text{for non-terminal } \phi_t \end{cases}$
    for $i \in \{t-1, \cdots, t_{start}\}$ **do**
      $R \leftarrow r_i + \gamma R$
      Accumulate the policy gradients: $\partial\theta_\pi \leftarrow \partial\theta_\pi + \nabla_\theta \log \pi(a_i \mid \phi_i; \theta)\big(R - V(\phi_i, \theta_v)\big)$
      Accumulate the value gradients: $\partial\theta_v \leftarrow \partial\theta_v + \frac{\partial\left(R - V(\phi_i, \theta_v)\right)^2}{\partial\theta_v}$
    end for
    Update θ_π and θ_v using $\partial\theta_\pi$ and $\partial\theta_v$, respectively.
    if ϕ_t is terminal **then**
      $e \leftarrow e + 1$
    end if
  until $e > e_{max}$
end for

Proximal Policy Optimization (PPO): PPO models are a family of policy gradient methods for reinforcement learning. The PPO method uses a slightly different training procedure: An extended set of samples is taken from the environment, and then the advantage is estimated for the whole set or sequence of samples before several epochs of training are performed To estimate policy gradients, instead of using the gradient of action probabilities, the PPO method uses a different objective: the ratio between the new and the old policy scaled by the advantages.

Once more, for the results represented in the main paper, we used the outcome of a grid search for the PPO algorithm in the same space as A2C above and utilized *actor learning rate = 0.003, 0.0001, 0.001*, *critic learning rate = 0.003, 0.001 , 0.001*, and *batch size = 16, 16, 32* to generate the results of Figure 5, 6, and 7, respectively.

Figure S6 represents the performance of the PPO algorithm with the Image Input design in terms of average rally length in several sample points of the mentioned search space. While exploring each hyper-parameter in Figure S6, the remaining pair are set to the same values as the starting point of the search (i.e. *actor learning rate = 0.0001*, *batch size = 32*, and *critic learning rate = 0.001*).

A.6 Additional Hyper-parameter Exploration

Effect of Batch Size on Deep RL Algorithm Performances:

From a technical standpoint, there exist no foolproof techniques for identifying the ideal hyper-parameter configuration for training deep RL algorithms. In addition, the batch size has an impact on the con-

Algorithm 3 Proximal Policy Optimization (PPO)

Require:

θ: Initial policy parameter vector
ϵ: Clipping threshold (Default: 0.2)
γ: Discount factor (Default: 0.95)
λ: GAE parameter (Default: 1)
N: Number of consecutive steps to play current policy in the environment (Default: 32)
x_t: Input matrix at time t
r_t: 1 if hit in t, 0 otherwise
S: Number of seeds (Default: 150)
e_{max}: Maximum number of episodes (Default: 70)
for seed $\in \{1, \cdots, S\}$ **do**
  $t = 1$
  $e = 1$
  repeat
    $t_{start} = t$
    Set state $s_t \leftarrow x_t$ and preprocess $\phi_t = \phi(s_t)$
    repeat
      Select a_t according to $\pi(a_t \mid \phi_t; \theta)$
      Execute action a_t and observe reward r_t and input x_{t+1}
      Set new state s_{t+1} and preprocess $\phi_{t+1} = \phi(s_{t+1})$
      $t \leftarrow t + 1$
    until ϕ_t is terminal **or** $t - t_{start} = N$
    Collect set of partial trajectories $\mathcal{D}$ on current policy π
    Estimate Advantages $\hat{A}_t^{\pi} = \sigma_t + (\gamma\lambda)\sigma_{t+1} + \cdots + (\gamma\lambda)^{N-t-1}\sigma_{N-1}$, where $\sigma_t = r_t + \gamma V(\phi_{t+1}) - V(\phi_t)$
    $\theta \leftarrow \text{argmax}_{\theta} \mathcal{L}_{\theta}^{CLIP}(\theta)$
    where $\mathcal{L}_{\theta}^{CLIP}(\theta) = \mathbb{E}_{\tau\sim\pi}\left[\sum_{t=0}^{T}\left[min(r_t(\theta)\hat{A}_t^{\pi}, clip(r_t(\theta), 1-\epsilon, 1+\epsilon)\hat{A}_t^{\pi})\right]\right]$
    if ϕ_t is terminal **then**
      $e \leftarrow e + 1$
    end if
  until $e > e_{max}$
end for

Table S1: Experimented Hyper-parameter and network architecture details

Hyper-parameter	Algorithm	Tested Values
Conv_1 size	DQN, A2C, PPO	**(16 × 16)**, (64 × 64)
Conv_2 size	DQN, A2C, PPO	**(32 × 32)**, (64 × 64)
Conv_3 size	DQN, A2C, PPO	**(32 × 32)**, (64 × 64)
Last hidden layer size	DQN, A2C, PPO	{100, 256, **512**}
Number of seeds	DQN, A2C, PPO	**150**
Kernel size	DQN, A2C, PPO	{**5**, 4}
Stride	DQN, A2C, PPO	**2**
Batch size	DQN, A2C, PPO	[5, 128]
Discount factor	DQN, A2C, PPO	{0.85, 0.95, 0.99, 0.999}
Learning rate	DQN	[0.0001, 0.004]
Replay buffer size	DQN	[10, 100000]
Actor-learning rate	A2C, PPO	[0.0001, 0.004]
Critic-learning rate	A2C, PPO	[0.0001, 0.004]
Clipping threshold	PPO	{0.1, **0.2**, 0.3}
Num of epochs	PPO	{**5**, 8, 10}

* The parameter values jointly chosen for all algorithms are highlighted in bold.

vergence rate of the prediction network, with smaller batch sizes resulting in faster convergence and well-known degradation in model quality and generalization abilities that can occur with increased batch sizes [95]. As such, originally we aimed to select batch sizes that would converge within sample numbers comparable to the training period of biological cultures while attempting to prioritize computational efficiency, which is a significant area of interest in this study. Hence, opting for large batch sizes may significantly slow down the model convergence and would not confer any benefit to the RL algorithms under investigation.

Figures S7, S8, and S9 investigate the impact of changing batch sizes utilizing the Image Input design by incorporating batch sizes of 8, 16, 32, and 64 while keeping the rest of the hyper-parameters in each algorithm fixed at default levels similar to Figure S6 (i.e. *learning rate = 0.0001*, *batch size = 32*, and *replay buffer size = 10000* for DQN and *actor learning rate = 0.0001*, *batch size = 32*, and *critic*

learning rate = 0.001 for A2C and PPO).

In some cases, these results illustrate an unwanted trend in the main metrics of interest when increasing the batch size above certain levels. For instance, an increasing % of aces in the DQN and PPO algorithms, decreasing average rally length in PPO, and decreasing % of long rallies in both A2C and PPO algorithms are observed which may eventually prevent the model from converging to the optima. This suggests that if the comparison were to be extended to a larger number of episodes for all groups, the increase in batch size would not necessarily yield improved performances, as evidenced by the undesirable trend observed in the aforementioned metrics (Extended Data Figures S7, S8, and S9). Notably, this may occur due to the fact that larger batch sizes make larger gradient steps than smaller batch sizes for the same number of samples seen and the update is heavily dependent on the specific samples drawn from the dataset. Conversely, a small batch size leads to updates that are more consistent in size, with the size of the update being only weakly dependent on which particular samples are selected from the dataset. In conclusion, it is possible that in deep neural networks, optimal weight configurations are located far from the initial weights. Hence, averaging the loss function over large batch sizes may not allow the model to explore a large enough space to reach the optimal weight configurations within the same number of training epochs.

Effects of Adding Hidden Layers:

To evaluate the effect of adding extra hidden layers on the performance of Ball Position Input and Paddle&Ball Position Input designs to the DQN algorithm, we implemented them by adding 2 additional hidden layers before the output layer and incorporating a batch size = 32. Extended Data Figure S10 shows the outcomes of these adjustments.

This further analysis revealed that although certain metrics exhibited qualitative and quantitative changes in their trends, the overall sample efficiency performance remained unaffected and even worsened with the inclusion of additional hidden layers. For example, we noted a degradation and an unwanted decreasing trend in the DQN's performance in the % of long rallies for the Paddle&Ball Position Input design. This resulted in the MCC group significantly outperforming DQN Paddle&Ball Position Input design in terms of % of long Rallies during the last 15 minutes. The performance of DQN in terms of average rally length was also deteriorated by the addition of these layers. On the other hand, MCC no longer demonstrated a significantly superior performance in terms of % of aces by the addition of hidden layers to the Paddle&Ball Position Input design. While some level of improvement was detected in the DQN group with the Ball Position Input design (specifically in the % of aces achieved) by the addition of the extra layers, overall performance in all 3 metrics was still inferior to those of the biological cultures in all the metrics. Specifically as illustrated in Extended Data Figure S11, the HCC group still demonstrated significant outperformance compared to the DQN Paddle&Ball Position Input and Ball Position Input designs in terms of relative improvement. The relative improvement in both of the Paddle&Ball Position Input and Ball Position Input designs showed a decay compared to the results reported in the main text, where this level of outperformance of MCC over DQN was not observed in the absence of hidden layers.

The observed deteriorated performance in terms of relative improvement in the Paddle&Ball Position Input design may be attributed to decreased generalization capabilities and higher variance resulting from the introduction of additional hidden layers. Because, for simpler tasks, a smaller network with fewer hidden layers might be sufficient to achieve good performance, and adding more layers could lead to overfitting. Thereby, this declined performance in the relative improvement as well as the low dimensionality of the input information in these designs combined with the faster computational performance of the algorithm with fewer hidden layers can justify the use of the shallower design for comparison reasons.

A.7 Active Inference Agent

While RL algorithms use back-propagation, it has been argued that this method is likely too inefficient to function within biological systems. Therefore, we attempted to evaluate the sample efficiency of more biologically inspired algorithms, by implementing a counterfactual learning active inference agent [96, 97]. Our preliminary findings show that one can use a generic active inference agent which can then mimic the performance of the *DishBrain* system depending on additional parameters such as memory.

The active inference framework is a formal way of modelling the behaviour of self-organising systems that interface with the external world and maintain a consistent form over time [98, 99, 100]. The framework assumes that agents embody generative models of the environment they interact with, on which they base their behaviour [101, 102]. A recent active inference scheme is shown to be mathematically equivalent to a particular class of neural networks accompanied by some neuromodulations of synaptic plasticity [96, 97]. It uses counterfactual learning (CL) to accumulate a measure of risk over time based on feedback from the environment. Subsequent work that validates this scheme experimentally using *in vitro* neural networks has also appeared recently [103]. Of particular note, the training schematic for the *DishBrain* system was inspired by implications from theory on active inference via the Free Energy Principle, making it the most suitable algorithm to compare here [48]. Here, we focus on generative models in the form of Partially Observable Markov Decision Processes (POMDPs) for their simplicity and ubiquitous use in the optimal control literature [104, 105, 106].

Gameplay performance of these agents with two different memory horizons of 3 (CL(3)) and 7 (CL(7)) is summarised in Figure S12. We see that the CL(7) agents perform at par and in some cases better than the HCC group and are the only group where the HCC has no significant outperformance over them in terms of the relative improvement in time (see Figure S12.h). However, this is not the case for CL(3) agents which have a smaller memory horizon. While further exploring this active inference framework is out of scope for this paper, it does highlight the value of using biologically inspired algorithms in terms of sample efficiency.

Generative model of the pong game environment:
Assuming agents have a discrete representation of their surrounding environment, we turn to the POMDP framework [106]. POMDPs offer a fairly expressive structure to model discrete state-space environments where parameters can be expressed as tractable categorical distributions. The POMDP-based generative model can be formally defined as a tuple of finite sets $(S, O, U, \mathbb{B}, \mathbb{A})$:

- ◦ $s \in S : S$ is a set of hidden states (s) causing observations o.
- ◦ $o \in O : O$ is a set of observations, where $o = s$, in the fully observable setting. In a partially observable setting, $o = f(s)$.
- ◦ $u \in U : U$ is a set of actions (u). E.g., $U = \{Up, Stay, Down\}$.
- ◦ $\mathbb{B}$: encodes the one-step transition dynamics, $P(s_t|s_{t-1}, u_{t-1})$ i.e., the probability that when action u_{t-1} is taken while being in state s_{t-1} (at time $t-1$) results in s_t at time t.
- ◦ $\mathbb{A}$: encodes the likelihood mapping, $P(o_\tau|s_\tau)$ for the partially observable setting.
- ◦ $\mathbb{D}$: Encodes the prior of the agent about the hidden state factor s.
- ◦ $\mathbb{E}$: Encodes the prior of the agent about actions u.

In a POMDP, the hidden states (s) generate observations (o) through the likelihood mapping $(\mathbb{A})$ in the form of a categorical distribution, $P(o_\tau|s_\tau) = \text{Cat}(\mathbb{A} \times s_\tau)$. $\mathbb{B}$ is a collection of square matrices $\mathbb{B}_u$, where $\mathbb{B}_u$ represents transition dynamics $P(s_t|s_{t-1}, u_{t-1} = u)$: The transition matrix $(\mathbb{B})$ determines the dynamics of s given the agent's action u as $P(s_t|s_{t-1}, u_{t-1}) = \text{Cat}(\mathbb{B}_{u_{t-1}} \times s_{t-1})$. In $[\mathbb{A} \times s_\tau]$ and $[\mathbb{B}_{u_\tau} \times s_\tau]$, s_τ is represented as a one-hot vector that is multiplied through regular matrix multiplication

[2]. The *Markovianity* of POMDPs means that state transitions are independent of history (i.e. state s_t only depends upon the state-action pair (s_{t-1}, u_{t-1}) and not $s_{t-2},\ u_{t-2}$ etc.).

The generative model can be summarised as follows,

$$
\begin{aligned}
P(o_{1:t}, s_{1:t}, u_{1:t}) &= P(\mathbb{A})P(\mathbb{B})P(\mathbb{D})P(\mathbb{E}) \\
&\times \prod_{\tau=1}^{t} P(o_\tau|s_\tau, \mathbb{A}) \prod_{\tau=2}^{t} P(s_\tau|s_{\tau-1}, u_{\tau-1}, \mathbb{B}).
\end{aligned} \tag{S1}
$$

So, from the agent's perspective, when encountering a stream of observations in time, such as $(o_1, o_2, o_3, ..., o_t)$, as a consequence of performing a stream of actions $(u_1, u_2, u_3, ..., u_{t-1})$, the generative model quantitatively couples and quantifies the causal relationship from action to observation through some assumed hidden states of the environment. These are called 'hidden' states because, in POMDPs, the agent cannot observe them directly. Based on this representation, an agent can now attempt to optimise its actions to keep receiving preferred observations.

The generative model structure used explicitly for the pong game environment is summarised below:

- $x-$**axis location of the ball:** Communicated to *DishBrain* using a stimulation between 4-40 HZ, i.e. 37 states.
- $y-$**axis location of the ball:** Communicated to *DishBrain* through 8 sensory electrodes, i.e. 8 states.
- $y-$**axis location of the paddle:** Assumed to be part of *DishBrain* 's generative model as control is exerted, i.e. 8 states.
- **Structure:** State Space = $37 * 8 * 8$ states, Action Space = {Up, Down, Stay}

Counterfactual learning algorithm:

In the counterfactual variant of active inference, the agent learns a state-action mapping $\mathbb{C}_{\mathbb{P}}$. For the exact form of the generative model and free energy, refer to [96]. This state-action mapping is learned using a 'Risk' parameter $\Gamma(t)$ using the update equation as given in [96] as:

$$\mathbb{C}_P \leftarrow \mathbb{C}_P + t\ \langle (1 - 2\ \Gamma(t)) \langle u_t \otimes s_{t-1} \rangle \rangle. \tag{S2}$$

Here, $\langle \cdot \rangle$ refers to the average over time, and $\otimes$ is the Kronecker-product operator. Given the state-action mapping $\mathbb{C}_P$, agent samples actions from the distribution,

$$P(u|s)_{CL} = \sigma \left(\ln\ \mathbb{C}_P \cdot s_{t-1} \right). \tag{S3}$$

For the complete model, refer to [96]. The free parameter in our model is the number of past instances (of state-action pairs) the agent stores in memory use in every time-step to learn $\mathbb{C}_P$ in Eq.S2. In the article, we use active inference agents with memory horizons of 3 and 7.

The functional form of $\Gamma(t)$ used in the simulations of this work is:

$$\Gamma(t)_{prior} = 0.55 \tag{S4}$$

The value of 0.55 corresponds to a bias of "higher risk" in the CL method. An initial value greater than 0.5 is necessary to enable learning.

For updating Γ, we use the equation,

$$\Gamma(t) \leftarrow \Gamma(t) - \frac{1}{T_{goal} - t}. \tag{S5}$$

[2]One-hot is a group of bits among which the legal combinations of values are only those with a single high (1) bit and all the others low (0). Here, the bit (1) is allocated to the state $s = s_\tau$

Here, T_{goal} is when the agent reaches the goal state (received a positive reward from the environment). So, the sooner the agent reaches the goal state, the quicker the $\Gamma(t)$, i.e., risk converges to zero. All the update rules defined in the paper can be derived from the postulate that the agent tries to minimise the (variational) free energy w.r.t the generative model [107, 96].

B Extended Data

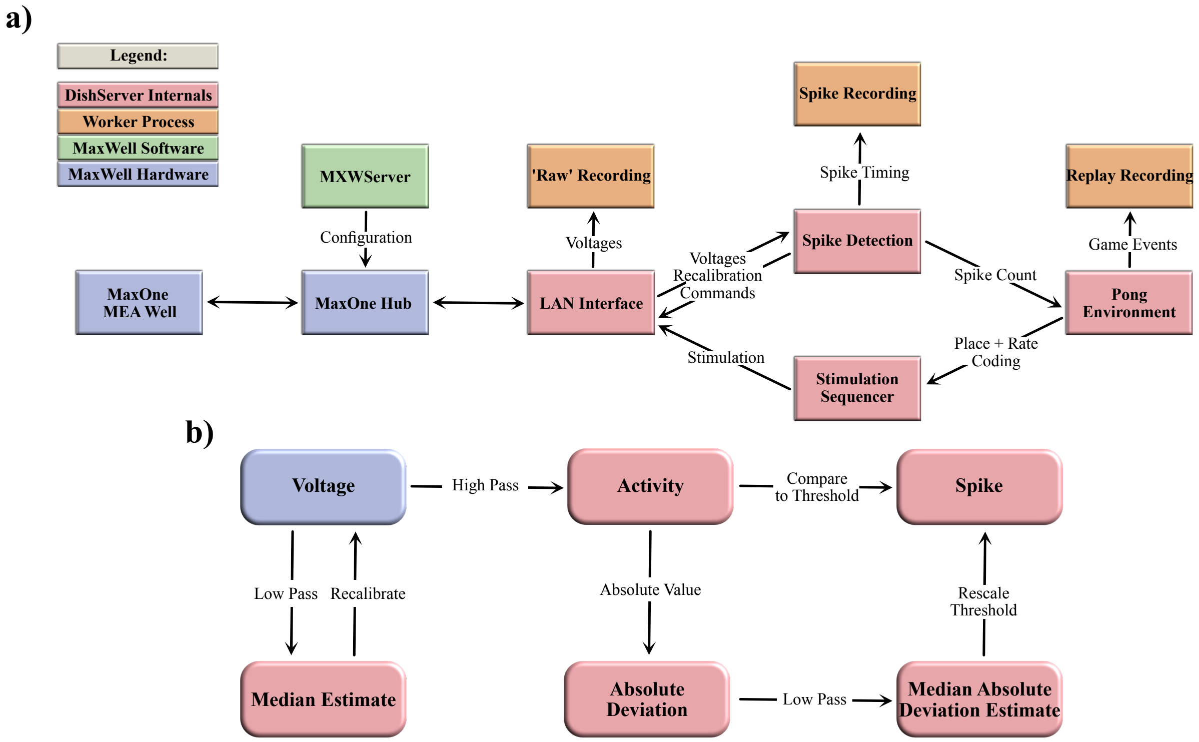

Figure S1: ***DishBrain* software schematics. a)** Software components and data flow in the *DishBrain* closed loop system. Voltage samples flow from the MEA to the 'Pong' environment, and sensory information flows from the 'Pong' environment back to the MEA, forming a closed loop. The blue rectangles mark proprietary pieces of hardware from MaxWell, including the MEA well which may contain a live culture of neurons. The green MXWServer is a piece of software provided by MaxWell which is used to configure the MEA and Hub, using a private API directly over the network. The red rectangles mark components of the 'DishServer' program, a high-performance program consisting of four components designed to run asynchronously, despite being run on a single CPU thread. The 'LAN Interface' component stores the network state, for talking to the Hub, and produces arrays of voltage values for processing. Voltage values are passed to the 'Spike Detection' component, which stores feedback values and spike counts, and passes recalibration commands back to the LAN Interface. When the pong environment is ready to run, it updates the state of the paddle based on the spike counts, updates the state of the ball based on its velocity and collision conditions, and re-configures the stimulation sequencer based on the relative position of the ball and current state of the game. The stimulation sequencer stores and updates indices and countdowns relating to the stimulations it must produce and converts these into commands each time the corresponding countdown reaches zero, which are finally passed back to the LAN Interface, to send to the MEA system, closing the loop. The procedures associated with each component are run one after the other in a simple loop control flow, but the 'Pong' environment only moves forward every 200th update, short-circuiting otherwise. Additionally, up to three worker processes are launched in parallel, depending on which parts of the system need to be recorded. They receive data from the main thread via shared memory and write it to file, allowing the main thread to continue processing data without having to hand control to the operating system and back again. **b)** Numeric operations in the real-time spike detection component of the *DishBrain* closed loop system, including multiple IIR filters. Running a virtual environment in a closed loop imposes strict performance requirements, and digital signal processing is the main bottleneck of this system, with close to 42 MB of data to process every second. Simple sequences of IIR digital filters are applied to incoming data, storing multiple arrays of 1024 feedback values in between each sample. First, spikes on the incoming data are detected by applying a high pass filter to determine the deviation of the activity and comparing that to the MAD, which is itself calculated with a subsequent low pass filter. Then, a low pass filter is applied to the original data to determine whether the MEA hardware needs to be re-calibrated, affecting future samples. This system was able to keep up with the incoming data on a single thread of an Intel Core i7-8809G. Figures adapted from [48].

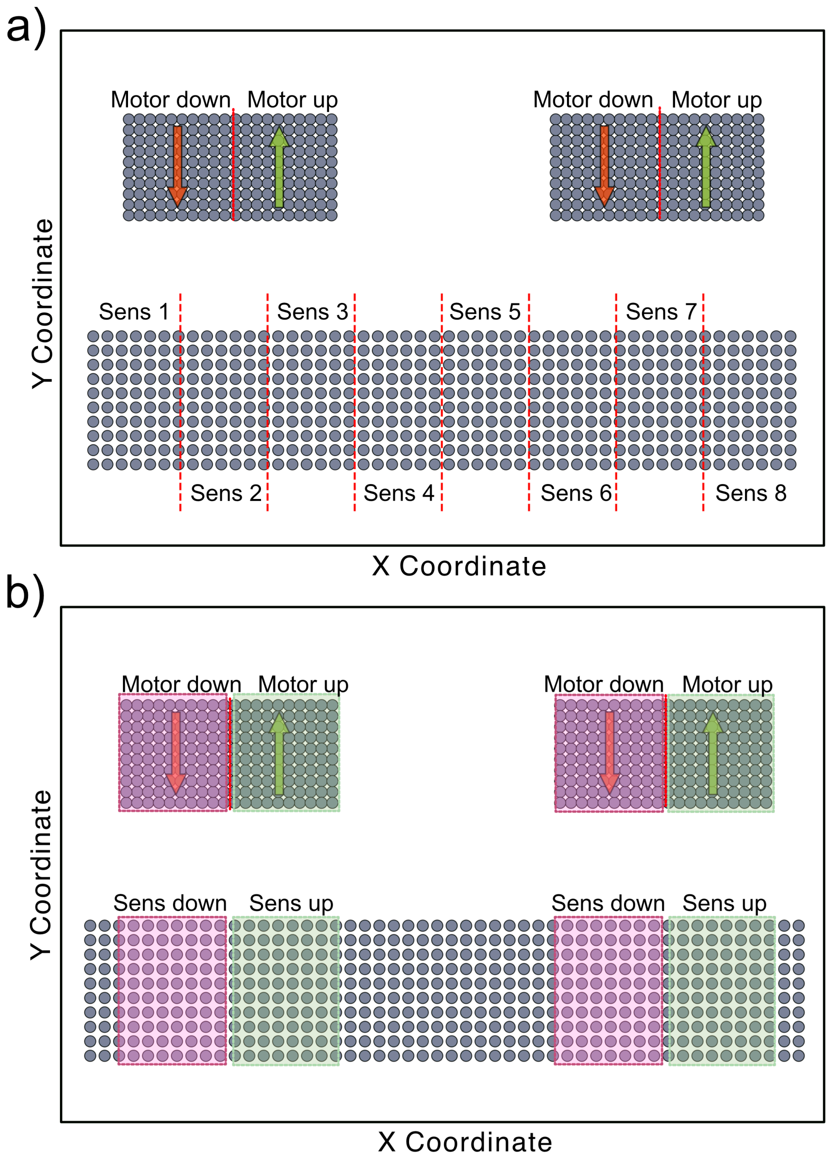

Figure S2: **Electrode layout divided into spatially defined sensorimotor regions. a)** 10-region scheme: electrodes are grouped into 8 sensory regions (Sens 1–8) along the x-axis and 2 motor regions (Motor Up, Motor Down). **b)** 4-region scheme: electrodes in the sensory region, which are aligned with the motor areas, are grouped into two sensory regions (Sensory Up and Sensory Down) facing Motor Up and Motor Down, respectively. These groupings were used to compute region-wise functional connectivity during *Gameplay* and *Rest*

.

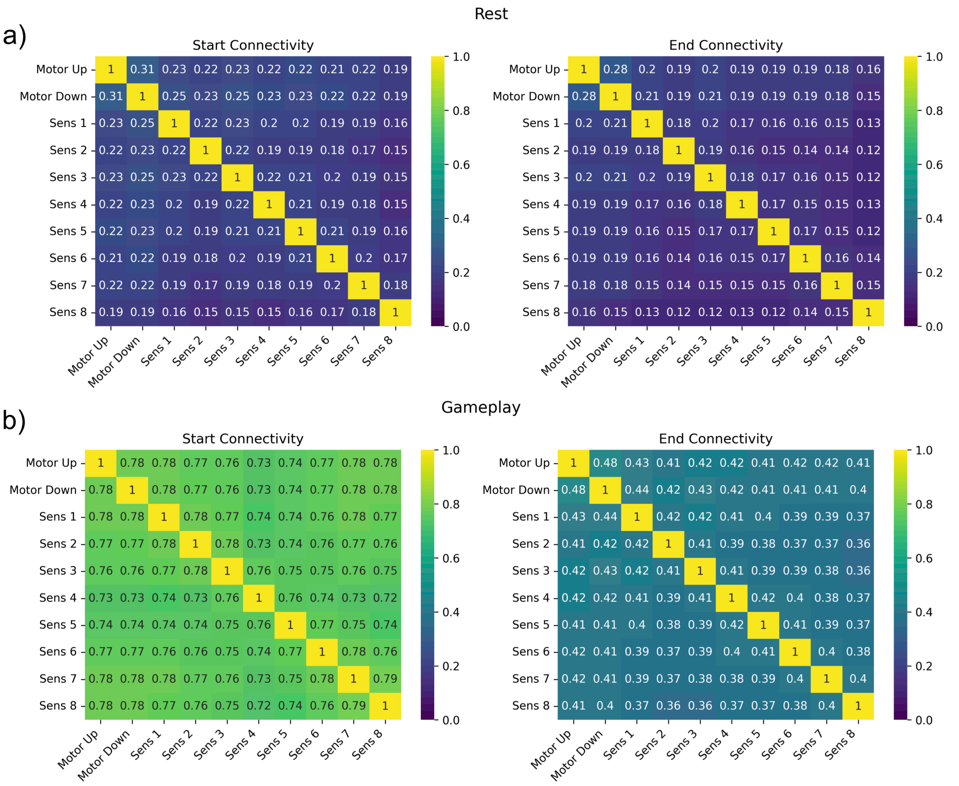

Figure S3: **Temporal Evolution of Region-Wise Functional Connectivity: 10 region scheme** Pairwise functional connectivity (Pearson correlation) between spatially grouped sensorimotor regions at the start and end of recordings. **a)** Connectivity matrices for the *Rest* condition an **b)** *Gameplay* conditions using the 10-region scheme (Motor Up, Motor Down, Sensory 1-8). Each subpanel compares the connectivity of aggregated electrode activities between pairs of regions during the first 2 minutes ("Start Connectivity") and the last 2 minutes ("End Connectivity") of the session.

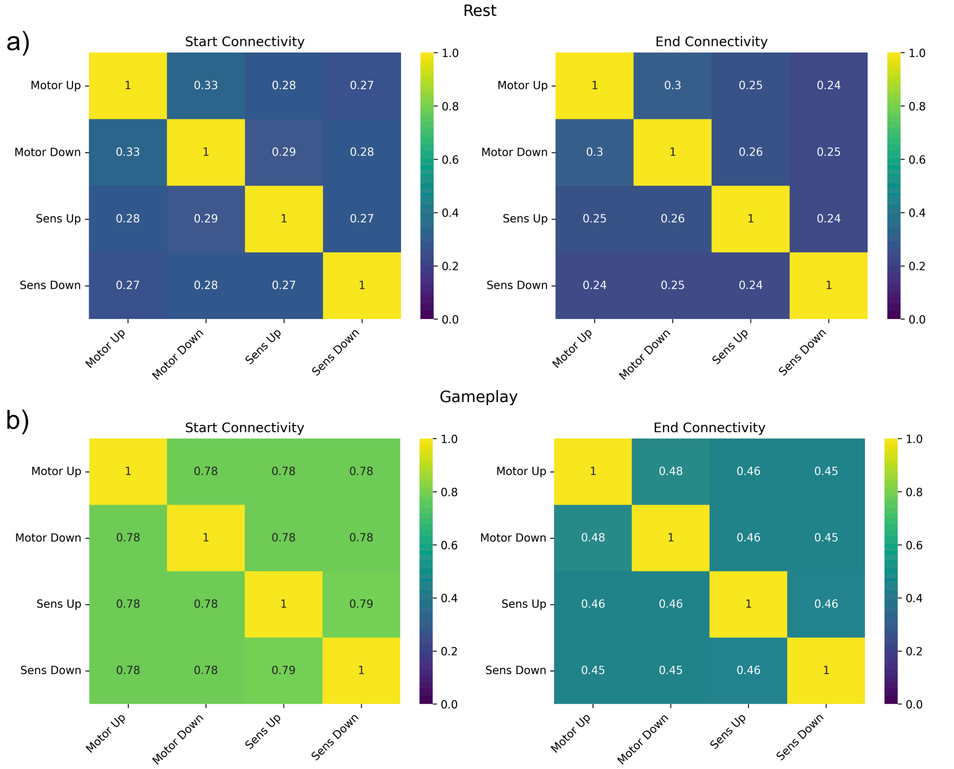

Figure S4: **Temporal Evolution of Region-Wise Functional Connectivity: 4 region scheme** Pairwise functional connectivity (Pearson correlation) between spatially grouped sensorimotor regions at the start and end of recordings. **a)** Connectivity matrices for the *Rest* condition an **b)** *Gameplay* conditions using the 4-region scheme (Motor Up, Motor Down, Sensory Up, Sensory Down). Each subpanel compares the connectivity of aggregated electrode activities between pairs of regions during the first 2 minutes ("Start Connectivity") and the last 2 minutes ("End Connectivity") of the session.

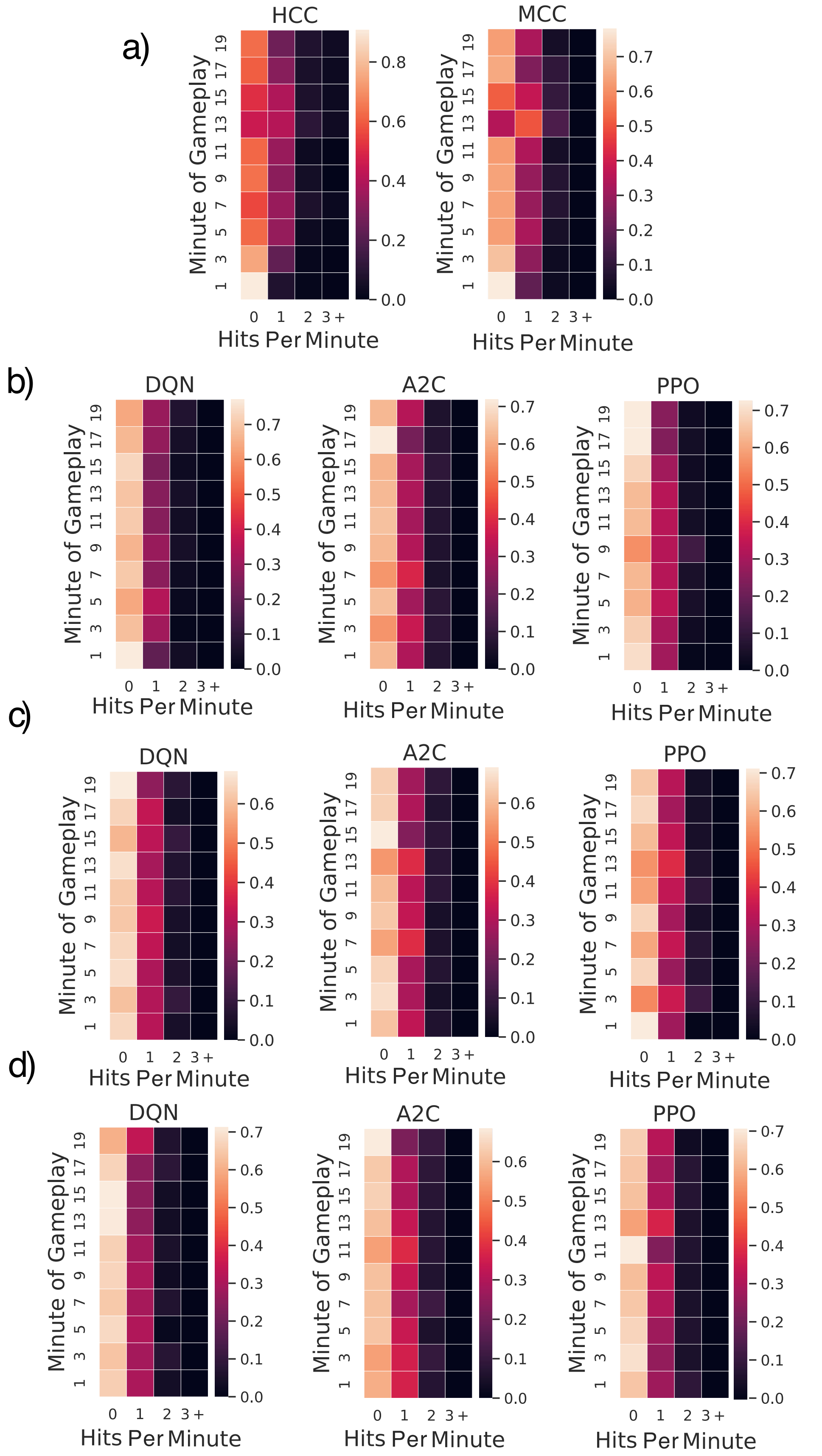

Figure S5: **Distribution of frequency of mean summed hits per minute** amongst groups for **a)** biological cultures and deep RL algorithms with **b)** Image Input, **c)** Paddle&Ball Position Input, and **d)** Ball Position Input.

.

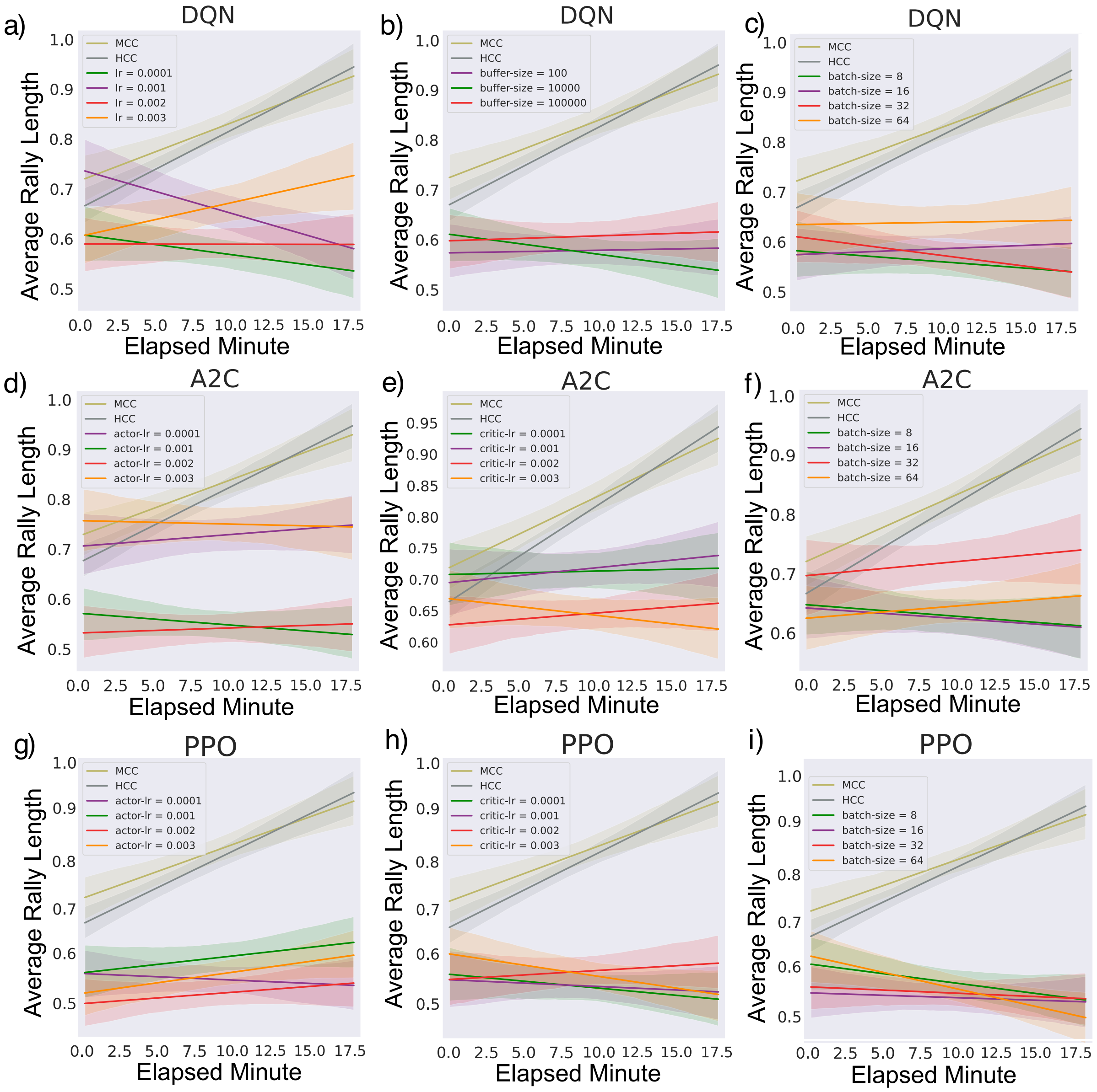

Figure S6: **Hyper-parameter exploration of RL algorithms.** The changes in average hits-per-rally for each RL algorithm in several sample points of the grid search space. **a)** Effects of changing the learning rate on DQN performance. replay buffer size = 10000 and batch size = 32; **b)** Effects of changing the replay buffer size on DQN performance. learning rate = 0.0001 and batch size = 32; **c)** Effects of changing the batch size on DQN performance. learning rate = 0.0001 and replay buffer size = 10000; **d)** Effects of changing the actor learning rate on A2C performance. critic learning rate = 0.001 and batch size = 32; **e)** Effects of changing the critic learning rate on A2C performance. actor learning rate = 0.0001 and batch size = 32; **f)** Effects of changing the batch size on A2C performance. actor learning rate = 0.0001 and critic learning rate = 0.001; **g)** Effects of changing the actor learning rate on PPO performance. critic learning rate = 0.001 and batch size = 32; **h)** Effects of changing the critic learning rate on PPO performance. actor learning rate = 0.0001 and batch size = 32; **i)** Effects of changing the batch size on PPO performance. actor learning rate = 0.0001 and critic learning rate = 0.001.

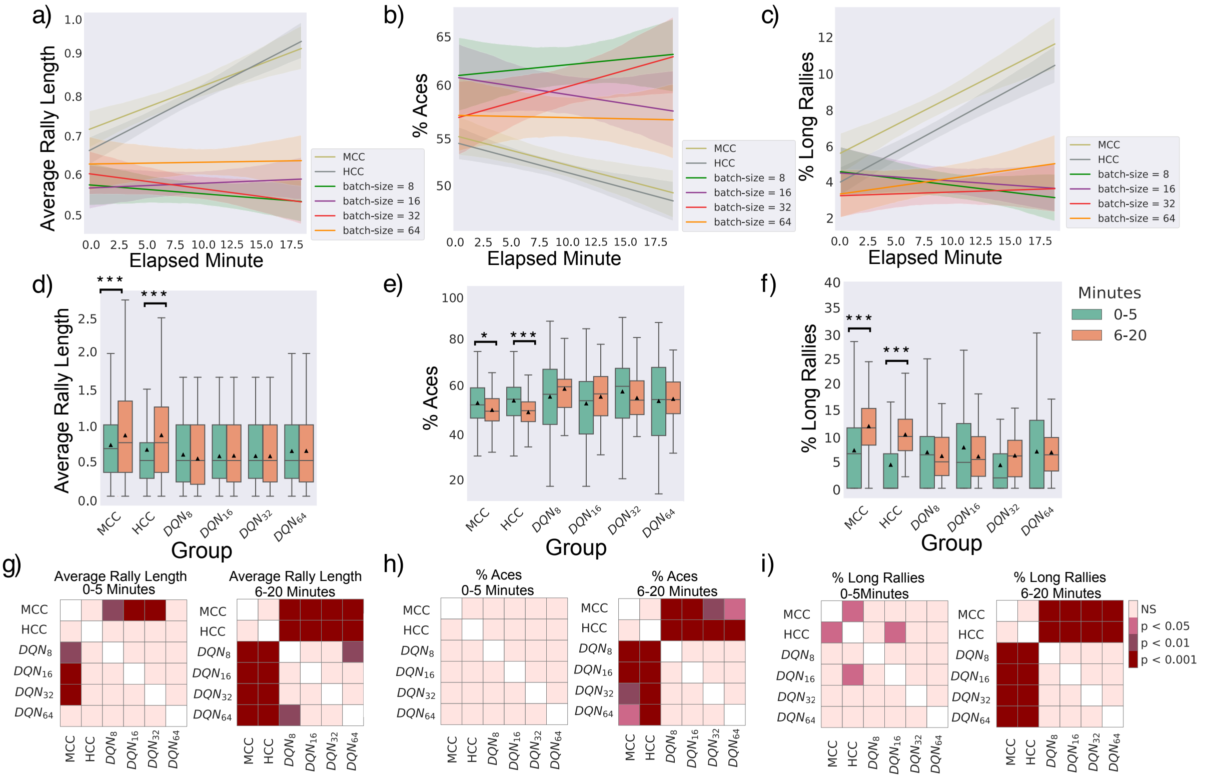

Figure S7: **Image Input to DQN - Effects of changing the batch size.** The Average number of **a)** hits-per-rally, **b)** % of aces, and **c)** % of long rallies over 20 minutes real-time equivalent of training DQN with batch sizes 8, 16, 32, 64, compared to the MCC and HCC cultures. **d)** average rally length over time, **e)** Average % of aces within groups and over time. **f)** Average % of long-rallies (≥ 3) performed in a session. **g,h and i)** Pairwise Tukey's post-hoc test. Box plots show interquartile range, with bars demonstrating 1.5X interquartile range, the line marks the median and the black triangle marks the mean. Error bands = 1 SE.

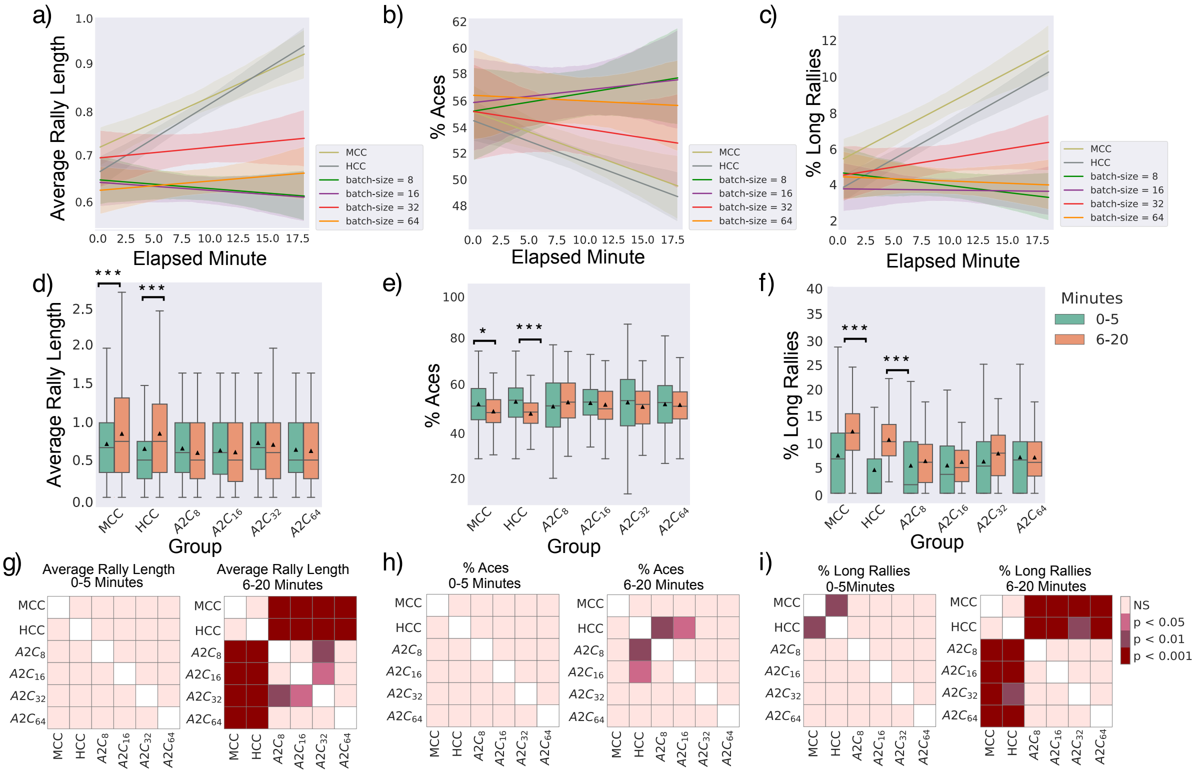

Figure S8: **Image Input to A2C - Effects of changing the batch size.** The Average number of **a)** hits-per-rally, **b)** % of aces, and **c)** % of long rallies over 20 minutes real-time equivalent of training A2C with batch sizes 8, 16, 32, 64, compared to the MCC and HCC cultures. **d)** average rally length over time, **e)** Average % of aces within groups and over time. **f)** Average % of long-rallies (≥ 3) performed in a session. **g,h and i)** Pairwise Tukey's post-hoc test. Box plots show interquartile range, with bars demonstrating 1.5X interquartile range, the line marks the median and the black triangle marks the mean. Error bands = 1 SE.

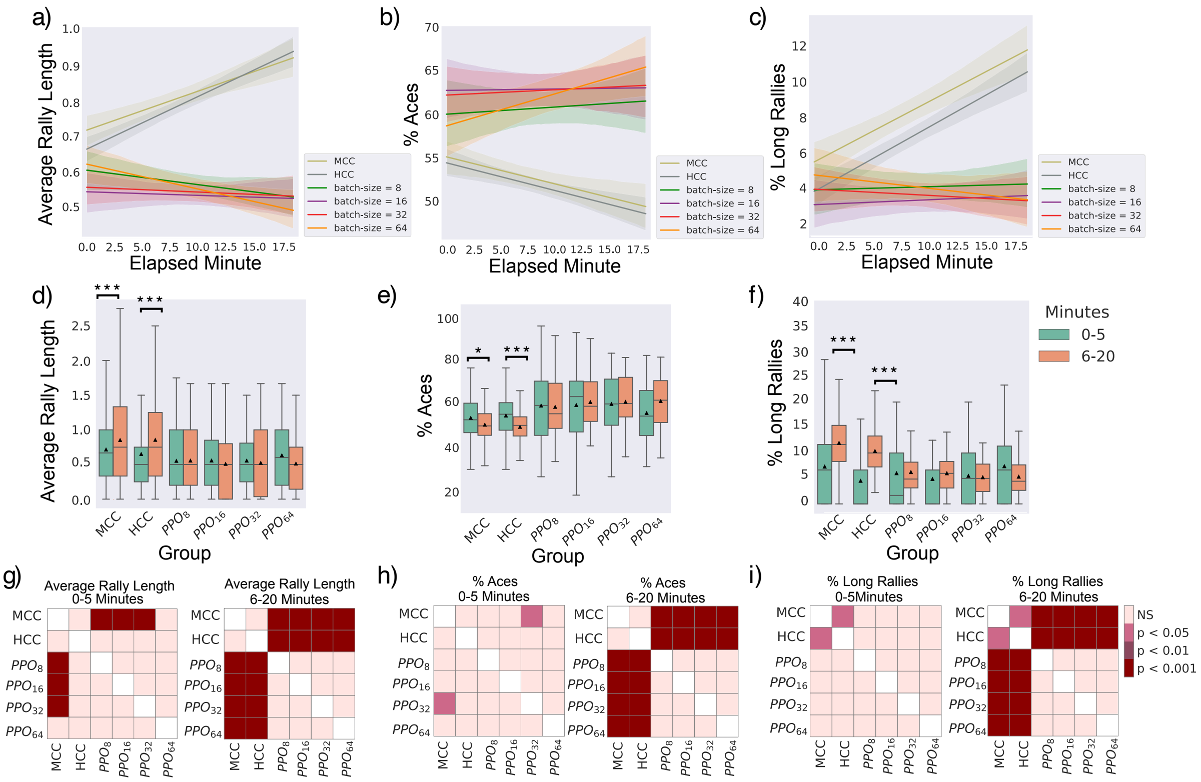

Figure S9: **Image Input to PPO - Effects of changing the batch size.** The Average number of **a)** hits-per-rally, **b)** % of aces, and **c)** % of long rallies over 20 minutes real-time equivalent of training PPO with batch sizes 8, 16, 32, 64, compared to the MCC and HCC cultures. **d)** average rally length over time, **e)** Average % of aces within groups and over time. **f)** Average % of long-rallies (≥ 3) performed in a session. **g,h and i)** Pairwise Tukey's post-hoc test. Box plots show interquartile range, with bars demonstrating 1.5X interquartile range, the line marks the median and the black triangle marks the mean. Error bands = 1 SE.

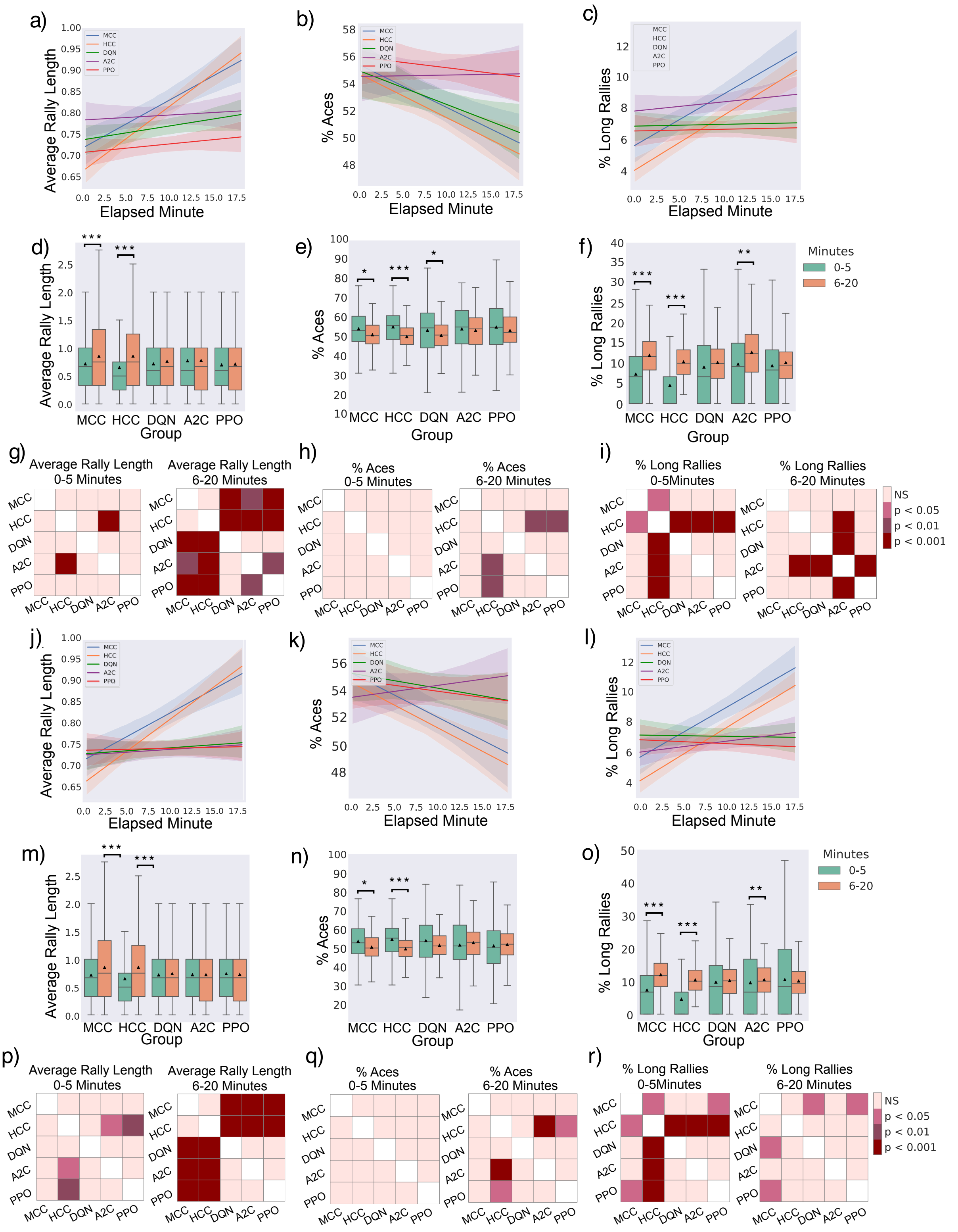

Figure S10: **Additional hidden layers in the DQN algorithm.** Ball Position Input to the RL Algorithms: The average number of **a)** hits-per-rally, **b)** % of aces, and **c)** % of long rallies over 20 minutes real-time equivalent of training DQN (2 additional hidden layers, batch size = 32), A2C, PPO, and MCC, HCC cultures. **d)** average rally length over time, **e)** Average % of aces within groups and over time. **f)** Average % of long-rallies (≥ 3) performed in a session. **g,h and i)** Pairwise Tukey's post-hoc test. Paddle&Ball Position Input to the RL Algorithms: The average number of **j)** hits-per-rally, **k)** % of aces, and **l)** % of long rallies over 20 minutes real-time equivalent of training DQN (2 additional hidden layers, batch size = 32), A2C, PPO, and MCC, HCC cultures. **m)** average rally length over time, **n)** Average % of aces within groups and over time. **o)** Average % of long-rallies (≥ 3) performed in a session. **p,q and r)** Pairwise Tukey's post hoc test. Box plots show interquartile range, with bars demonstrating 1.5X interquartile range, the line marks the median and the black triangle marks the mean. Error bands = 1 SE.

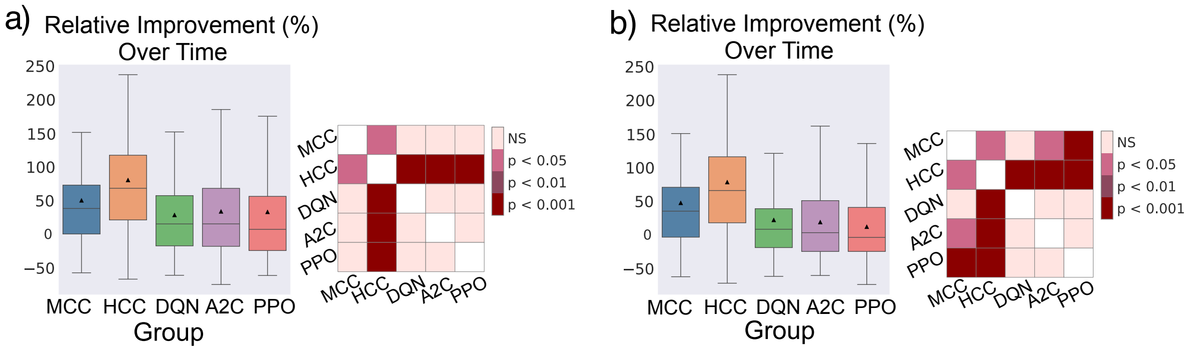

Figure S11: **Relative improvement over time with additional hidden layers in DQN algorithm.** Relative improvement (%) in the average hit counts between the first 5 minutes and the last 15 minutes of all sessions in each separate group for **a)** BALL POSITION INPUT design for DQN with 2 additional hidden layers, **b)** PADDLE&BALL POSITION INPUT design for DQN with 2 additional hidden layers.

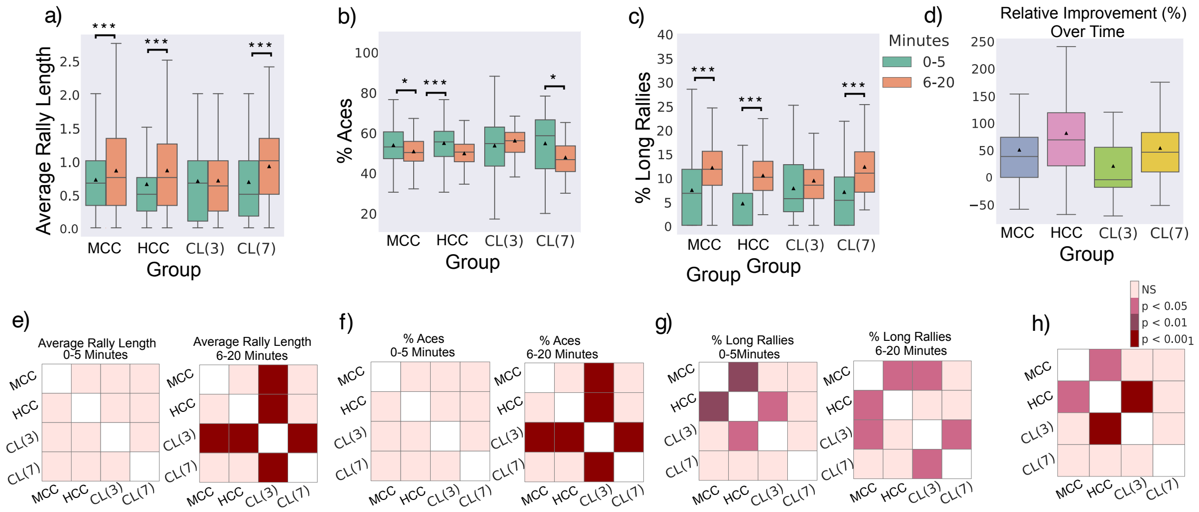

Figure S12: **Comparing Active Inference agent with biological neurons.** **a)** Average rally length over time where this within-group increase was significant for all groups except CL(3) (One-way ANOVA test, p = 5.854e-6, p = 7.936-17, p = 0.873, and p = 2.254e-6, for MCC, HCC, CL(3), and CL(7) respectively). **b)** Average % of aces within groups and over time where this within-group increase was significant only for HCC, MCC, and CL(7) groups (One-way ANOVA test, p = 0.014, p = 2.907e-08, p = 0.380, and p = 0.016, for MCC, HCC, CL(3), and CL(7) respectively). **c)** Average % of long-rallies (≥ 3) performed in a session where the increase over time was significant for all groups except CL(3) (One-way ANOVA test, p = 1.172e-7, p = 1.525e-24, p = 0.253, and p = 8.944e-4 for MCC, HCC, CL(3), and CL(7), respectively). **d** Relative improvement (%) in the average hit counts between the first 5 minutes and the last 15 minutes of all sessions in each separate group. **e,f,g and h)** Pairwise post hoc tests. Box plots show interquartile range, with bars demonstrating 1.5X interquartile range, the line marks the median and the black triangle marks the mean. Error bands = 1 SE.

Table S2. Follow up main and supplementary text post-hoc tests for multivariate tests, including means, standard error (SE), t-scores, degree of freedom and exact p-values with hedges.

Figure	Panel	Parameters	Source	A	B	Mean (A)	Mean (B)	diff	se	T	p-val	hedges	Method
**5**	h	Hit Counts	0-5 Minutes	A2C	DQN	0.705	0.709	-0.004	0.027	-0.147	0.900	-0.008	Tukey's
				A2C	HCC	0.705	0.651	0.055	0.025	2.151	0.199	0.110	
				A2C	MCC	0.705	0.716	-0.011	0.029	-0.373	0.900	-0.021	
				A2C	PPO	0.705	0.716	-0.011	0.029	-0.388	0.900	-0.022	
				DQN	HCC	0.709	0.651	0.059	0.025	2.310	0.142	0.117	
				DQN	MCC	0.709	0.716	-0.007	0.029	-0.237	0.900	-0.014	
				DQN	PPO	0.709	0.716	-0.007	0.029	-0.251	0.900	-0.014	
				HCC	MCC	0.651	0.716	-0.065	0.027	-2.386	0.120	-0.131	
				HCC	PPO	0.651	0.716	-0.066	0.027	-2.410	0.113	-0.132	
				MCC	PPO	0.716	0.716	-0.000	0.030	-0.013	0.900	-0.001	
			6-20 Minutes	A2C	DQN	0.738	0.738	0.000	0.018	0.004	0.900	0.000	
				A2C	HCC	0.738	0.854	-0.117	0.017	-6.726	0.001	-0.198	
				A2C	MCC	0.738	0.852	-0.115	0.020	-5.715	0.001	-0.194	
				A2C	PPO	0.738	0.709	0.029	0.019	1.506	0.551	0.049	
				DQN	HCC	0.738	0.854	-0.117	0.017	-6.737	0.001	-0.198	
				DQN	MCC	0.738	0.852	-0.115	0.020	-5.723	0.001	-0.194	
				DQN	PPO	0.738	0.709	0.029	0.019	1.503	0.552	0.049	
				HCC	MCC	0.854	0.852	0.002	0.020	0.101	0.900	0.003	
				HCC	PPO	0.854	0.709	0.146	0.019	7.818	0.001	0.246	
				MCC	PPO	0.852	0.709	0.144	0.021	6.778	0.001	0.243	
	i	% Aces	0-5 Minutes	A2C	DQN	51.842	52.190	-0.347	1.425	-0.244	0.900	-0.028	Tukey's
				A2C	HCC	51.842	54.382	-2.539	1.375	-1.847	0.348	-0.205	
				A2C	MCC	51.842	53.333	-1.490	1.549	-0.962	0.859	-0.120	
				A2C	PPO	51.842	54.731	-2.889	1.425	-2.028	0.254	-0.234	
				DQN	HCC	52.190	54.382	-2.192	1.375	-1.595	0.501	-0.177	
				DQN	MCC	52.190	53.333	-1.143	1.549	-0.738	0.900	-0.092	
				DQN	PPO	52.190	54.731	-2.542	1.425	-1.784	0.385	-0.205	
				HCC	MCC	54.382	53.333	1.049	1.503	0.698	0.900	0.085	
				HCC	PPO	54.382	54.731	-0.350	1.375	-0.254	0.900	-0.028	

				MCC	PPO	53.333	54.731	-1.399	1.549	-0.903	0.893	-0.113	
			6-20 Minutes	A2C	DQN	50.284	50.136	0.148	0.946	0.157	0.900	0.018	
				A2C	HCC	50.284	49.259	1.025	0.912	1.123	0.768	0.125	
				A2C	MCC	50.284	50.232	0.052	1.028	0.051	0.900	0.006	
				A2C	PPO	50.284	53.254	-2.970	0.946	-3.141	0.015	-0.362	
				DQN	HCC	50.136	49.259	0.877	0.912	0.961	0.860	0.107	
				DQN	MCC	50.136	50.232	-0.096	1.028	-0.093	0.900	-0.012	
				DQN	PPO	50.136	53.254	-3.118	0.946	-3.298	0.009	-0.380	
				HCC	MCC	49.259	50.232	-0.973	0.998	-0.975	0.852	-0.118	
				HCC	PPO	49.259	53.254	-3.995	0.912	-4.378	0.001	-0.487	
				MCC	PPO	50.232	53.254	-3.022	1.028	-2.940	0.028	-0.368	
	j	% Long Rally	0-5 Minutes	A2C	DQN	7.421	9.789	-2.368	0.913	-2.594	0.073	-0.299	Tukey's
				A2C	HCC	7.421	4.523	2.898	0.881	3.290	0.009	0.366	
				A2C	MCC	7.421	7.318	0.103	0.993	0.103	0.900	0.013	
				A2C	PPO	7.421	8.122	-0.701	0.913	-0.767	0.900	-0.088	
				DQN	HCC	9.789	4.523	5.267	0.881	5.978	0.001	0.665	
				DQN	MCC	9.789	7.318	2.471	0.993	2.489	0.094	0.312	
				DQN	PPO	9.789	8.122	1.667	0.913	1.826	0.360	0.210	
				HCC	MCC	4.523	7.318	-2.796	0.963	-2.903	0.031	-0.353	
				HCC	PPO	4.523	8.122	-3.599	0.881	-4.085	0.001	-0.454	
				MCC	PPO	7.318	8.122	-0.803	0.993	-0.809	0.900	-0.101	
			6-20 Minutes	A2C	DQN	10.034	10.248	-0.214	0.623	-0.344	0.900	-0.040	
				A2C	HCC	10.034	10.365	-0.331	0.601	-0.550	0.900	-0.061	
				A2C	MCC	10.034	11.972	-1.938	0.677	-2.863	0.035	-0.358	
				A2C	PPO	10.034	8.506	1.528	0.623	2.454	0.102	0.283	
				DQN	HCC	10.248	10.365	-0.116	0.601	-0.194	0.900	-0.022	
				DQN	MCC	10.248	11.972	-1.724	0.677	-2.547	0.082	-0.319	
				DQN	PPO	10.248	8.506	1.743	0.623	2.798	0.042	0.322	
				HCC	MCC	10.365	11.972	-1.608	0.657	-2.447	0.104	-0.297	
				HCC	PPO	10.365	8.506	1.859	0.601	3.094	0.017	0.344	
				MCC	PPO	11.972	8.506	3.467	0.677	5.121	0.001	0.641	
**6**	h	Hit Counts	0-5 Minutes	A2C	DQN	0.722	0.713	0.009	0.027	0.325	0.900	0.017	Tukey's
				A2C	HCC	0.722	0.651	0.072	0.026	2.761	0.046	0.141	
				A2C	MCC	0.722	0.716	0.006	0.029	0.216	0.900	0.012	

1313

				A2C	PPO	0.722	0.740	-0.018	0.027	-0.641	0.900	-0.035	
				DQN	HCC	0.713	0.651	0.063	0.026	2.428	0.108	0.123	
				DQN	MCC	0.713	0.716	-0.003	0.029	-0.087	0.900	-0.005	
				DQN	PPO	0.713	0.740	-0.026	0.027	-0.968	0.856	-0.052	
				HCC	MCC	0.651	0.716	-0.065	0.028	-2.335	0.134	-0.128	
				HCC	PPO	0.651	0.740	-0.089	0.026	-3.428	0.006	-0.175	
				MCC	PPO	0.716	0.740	-0.024	0.029	-0.815	0.900	-0.047	
			6-20 Minutes	A2C	DQN	0.724	0.716	0.008	0.018	0.415	0.900	0.013	
				A2C	HCC	0.724	0.854	-0.131	0.018	-7.461	0.001	-0.220	
				A2C	MCC	0.724	0.852	-0.129	0.020	-6.354	0.001	-0.216	
				A2C	PPO	0.724	0.727	-0.004	0.018	-0.217	0.900	-0.007	
				DQN	HCC	0.716	0.854	-0.138	0.017	-7.918	0.001	-0.232	
				DQN	MCC	0.716	0.852	-0.136	0.020	-6.743	0.001	-0.229	
				DQN	PPO	0.716	0.727	-0.011	0.018	-0.633	0.900	-0.019	
				HCC	MCC	0.854	0.852	0.002	0.020	0.100	0.900	0.003	
				HCC	PPO	0.854	0.727	0.127	0.018	7.233	0.001	0.213	
				MCC	PPO	0.852	0.727	0.125	0.020	6.158	0.001	0.210	
	i	% Aces	0-5 Minutes	A2C	DQN	51.318	54.016	-2.698	1.469	-1.837	0.354	-0.212	Tukey's
				A2C	HCC	51.318	54.382	-3.064	1.417	-2.162	0.196	-0.240	
				A2C	MCC	51.318	53.333	-2.014	1.597	-1.262	0.690	-0.158	
				A2C	PPO	51.318	50.866	0.453	1.469	0.308	0.900	0.035	
				DQN	HCC	54.016	54.382	-0.366	1.417	-0.258	0.900	-0.029	
				DQN	MCC	54.016	53.333	0.683	1.597	0.428	0.900	0.054	
				DQN	PPO	54.016	50.866	3.150	1.469	2.145	0.202	0.247	
				HCC	MCC	54.382	53.333	1.049	1.550	0.677	0.900	0.082	
				HCC	PPO	54.382	50.866	3.516	1.417	2.481	0.096	0.276	
				MCC	PPO	53.333	50.866	2.467	1.597	1.545	0.529	0.193	
			6-20 Minutes	A2C	DQN	52.596	53.001	-0.404	0.919	-0.440	0.900	-0.051	
				A2C	HCC	52.596	49.259	3.337	0.887	3.762	0.002	0.418	
				A2C	MCC	52.596	50.232	2.364	0.999	2.366	0.126	0.296	
				A2C	PPO	52.596	51.658	0.938	0.919	1.020	0.826	0.118	
				DQN	HCC	53.001	49.259	3.741	0.887	4.218	0.001	0.469	
				DQN	MCC	53.001	50.232	2.769	0.999	2.771	0.045	0.347	
				DQN	PPO	53.001	51.658	1.342	0.919	1.460	0.577	0.168	

				HCC	MCC	49.259	50.232	-0.973	0.970	-1.003	0.836	-0.122	
				HCC	PPO	49.259	51.658	-2.399	0.887	-2.705	0.054	-0.301	
				MCC	PPO	50.232	51.658	-1.427	0.999	-1.428	0.595	-0.179	
	j	% Long Rally	0-5 Minutes	A2C	DQN	9.519	10.105	-0.586	0.990	-0.591	0.900	-0.068	Tukey’s
				A2C	HCC	9.519	4.523	4.997	0.955	5.230	0.001	0.581	
				A2C	MCC	9.519	7.318	2.201	1.076	2.045	0.246	0.256	
				A2C	PPO	9.519	10.462	-0.942	0.990	-0.952	0.865	-0.110	
				DQN	HCC	10.105	4.523	5.582	0.955	5.843	0.001	0.650	
				DQN	MCC	10.105	7.318	2.787	1.076	2.589	0.074	0.324	
				DQN	PPO	10.105	10.462	-0.357	0.990	-0.360	0.900	-0.042	
				HCC	MCC	4.523	7.318	-2.796	1.044	-2.677	0.059	-0.325	
				HCC	PPO	4.523	10.462	-5.939	0.955	-6.217	0.001	-0.691	
				MCC	PPO	7.318	10.462	-3.144	1.076	-2.921	0.030	-0.366	
			6-20 Minutes	A2C	DQN	10.431	11.238	-0.807	0.616	-1.311	0.661	-0.151	
				A2C	HCC	10.431	10.365	0.066	0.594	0.111	0.900	0.012	
				A2C	MCC	10.431	11.972	-1.541	0.669	-2.303	0.145	-0.288	
				A2C	PPO	10.431	10.049	0.382	0.616	0.620	0.900	0.071	
				DQN	HCC	11.238	10.365	0.873	0.594	1.470	0.571	0.163	
				DQN	MCC	11.238	11.972	-0.734	0.669	-1.097	0.783	-0.137	
				DQN	PPO	11.238	10.049	1.189	0.616	1.931	0.302	0.222	
				HCC	MCC	10.365	11.972	-1.608	0.649	-2.475	0.097	-0.301	
				HCC	PPO	10.365	10.049	0.316	0.594	0.531	0.900	0.059	
				MCC	PPO	11.972	10.049	1.923	0.669	2.873	0.034	0.360	
**7**	h	Hit Counts	0-5 Minutes	A2C	DQN	0.771	0.687	0.084	0.028	2.980	0.024	0.159	Tukey’s
				A2C	HCC	0.771	0.651	0.121	0.027	4.507	0.001	0.229	
				A2C	MCC	0.771	0.716	0.055	0.030	1.826	0.359	0.105	
				A2C	PPO	0.771	0.698	0.073	0.028	2.593	0.072	0.139	
				DQN	HCC	0.687	0.651	0.037	0.027	1.371	0.628	0.070	
				DQN	MCC	0.687	0.716	-0.029	0.030	-0.951	0.866	-0.055	
				DQN	PPO	0.687	0.698	-0.011	0.028	-0.375	0.900	-0.020	
				HCC	MCC	0.651	0.716	-0.065	0.029	-2.262	0.158	-0.124	
				HCC	PPO	0.651	0.698	-0.047	0.027	-1.759	0.399	-0.090	
				MCC	PPO	0.716	0.698	0.018	0.030	0.598	0.900	0.034	
				A2C	DQN	0.777	0.687	0.090	0.018	4.982	0.001	0.150	

			6-20 Minutes	A2C	HCC	0.777	0.854	-0.077	0.018	-4.348	0.001	-0.128	
				A2C	MCC	0.777	0.852	-0.075	0.020	-3.662	0.002	-0.125	
				A2C	PPO	0.777	0.712	0.065	0.018	3.576	0.003	0.108	
				DQN	HCC	0.687	0.854	-0.167	0.018	-9.521	0.001	-0.278	
				DQN	MCC	0.687	0.852	-0.165	0.020	-8.119	0.001	-0.275	
				DQN	PPO	0.687	0.712	-0.025	0.018	-1.389	0.617	-0.042	
				HCC	MCC	0.854	0.852	0.002	0.020	0.099	0.900	0.003	
				HCC	PPO	0.854	0.712	0.142	0.018	8.044	0.001	0.236	
				MCC	PPO	0.852	0.712	0.140	0.020	6.854	0.001	0.233	
	i	% Aces	0-5 Minutes	A2C	DQN	53.293	55.443	-2.150	1.473	-1.459	0.577	-0.168	Tukey's
				A2C	HCC	53.293	54.382	-1.089	1.422	-0.766	0.900	-0.085	
				A2C	MCC	53.293	53.333	-0.040	1.602	-0.025	0.900	-0.003	
				A2C	PPO	53.293	54.248	-0.956	1.473	-0.649	0.900	-0.075	
				DQN	HCC	55.443	54.382	1.061	1.422	0.746	0.900	0.083	
				DQN	MCC	55.443	53.333	2.110	1.602	1.317	0.658	0.165	
				DQN	PPO	55.443	54.248	1.194	1.473	0.811	0.900	0.093	
				HCC	MCC	54.382	53.333	1.049	1.554	0.675	0.900	0.082	
				HCC	PPO	54.382	54.248	0.133	1.422	0.094	0.900	0.010	
				MCC	PPO	53.333	54.248	-0.916	1.602	-0.572	0.900	-0.072	
			6-20 Minutes	A2C	DQN	52.530	53.879	-1.349	0.966	-1.397	0.613	-0.161	
				A2C	HCC	52.530	49.259	3.270	0.932	3.508	0.004	0.390	
				A2C	MCC	52.530	50.232	2.298	1.050	2.188	0.185	0.274	
				A2C	PPO	52.530	52.511	0.018	0.966	0.019	0.900	0.002	
				DQN	HCC	53.879	49.259	4.620	0.932	4.955	0.001	0.551	
				DQN	MCC	53.879	50.232	3.647	1.050	3.472	0.005	0.435	
				DQN	PPO	53.879	52.511	1.368	0.966	1.415	0.602	0.163	
				HCC	MCC	49.259	50.232	-0.973	1.019	-0.954	0.864	-0.116	
				HCC	PPO	49.259	52.511	-3.252	0.932	-3.488	0.005	-0.388	
				MCC	PPO	50.232	52.511	-2.280	1.050	-2.170	0.192	-0.272	
	j	% Long Rally	0-5 Minutes	A2C	DQN	9.810	9.554	0.256	0.935	0.274	0.900	0.032	Tukey's
				A2C	HCC	9.810	4.523	5.288	0.902	5.861	0.001	0.652	
				A2C	MCC	9.810	7.318	2.492	1.016	2.452	0.103	0.307	
				A2C	PPO	9.810	9.403	0.408	0.935	0.436	0.900	0.050	
				DQN	HCC	9.554	4.523	5.032	0.902	5.577	0.001	0.620	

				DQN	MCC	9.554	7.318	2.236	1.016	2.200	0.181	0.275	
				DQN	PPO	9.554	9.403	0.151	0.935	0.162	0.900	0.019	
				HCC	MCC	4.523	7.318	-2.796	0.986	-2.834	0.038	-0.344	
				HCC	PPO	4.523	9.403	-4.880	0.902	-5.410	0.001	-0.601	
				MCC	PPO	7.318	9.403	-2.085	1.016	-2.051	0.243	-0.257	
			6-20 Minutes	A2C	DQN	12.722	9.511	3.211	0.632	5.083	0.001	0.585	
				A2C	HCC	12.722	10.365	2.357	0.610	3.868	0.001	0.430	
				A2C	MCC	12.722	11.972	0.750	0.687	1.092	0.786	0.137	
				A2C	PPO	12.722	10.183	2.540	0.632	4.020	0.001	0.463	
				DQN	HCC	9.511	10.365	-0.854	0.610	-1.401	0.611	-0.156	
				DQN	MCC	9.511	11.972	-2.461	0.687	-3.584	0.003	-0.449	
				DQN	PPO	9.511	10.183	-0.672	0.632	-1.063	0.802	-0.122	
				HCC	MCC	10.365	11.972	-1.608	0.666	-2.412	0.113	-0.293	
				HCC	PPO	10.365	10.183	0.182	0.610	0.299	0.900	0.033	
				MCC	PPO	11.972	10.183	1.790	0.687	2.606	0.070	0.326	
**8**	a	Average Paddle Movement-Image input		A2C	DQN	71606.154	75257.436	-3651.282	4997.725	-0.731	0.900	-0.164	Tukey's
				A2C	HCC	71606.154	52000.427	19605.727	3783.228	5.182	0.001	0.886	
				A2C	MCC	71606.154	50007.504	21598.650	4190.720	5.154	0.001	0.973	
				A2C	PPO	71606.154	72712.500	-1106.346	4966.391	-0.223	0.900	-0.050	
				DQN	HCC	75257.436	52000.427	23257.009	3783.228	6.147	0.001	1.051	
				DQN	MCC	75257.436	50007.504	25249.932	4190.720	6.025	0.001	1.138	
				DQN	PPO	75257.436	72712.500	2544.936	4966.391	0.512	0.900	0.114	
				HCC	MCC	52000.427	50007.504	1992.923	2626.345	0.759	0.900	0.090	
				HCC	PPO	52000.427	72712.500	-20712.073	3741.737	-5.535	0.001	-0.936	
				MCC	PPO	50007.504	72712.500	-22704.996	4153.302	-5.467	0.001	-1.023	
	d	Relative improvement (%) in the average hit counts – Image input		A2C	DQN	29.919	24.634	5.285	7.934	288.957	0.900	0.077	Games Howell
				A2C	HCC	29.919	82.147	-52.227	9.623	316.974	0.001	-0.603	
				A2C	MCC	29.919	50.755	-20.836	9.830	223.464	0.215	-0.265	

				A2C	PPO	29.919	21.602	8.318	7.665	279.006	0.789	0.125	
				DQN	HCC	24.634	82.147	-57.512	9.026	296.959	0.001	-0.708	
				DQN	MCC	24.634	50.755	-26.121	9.246	197.121	0.041	-0.354	
				DQN	PPO	24.634	21.602	3.033	6.900	295.706	0.900	0.051	
				HCC	MCC	82.147	50.755	31.391	10.731	262.994	0.030	0.355	
				HCC	PPO	82.147	21.602	60.545	8.791	284.214	0.001	0.766	
				MCC	PPO	50.755	21.602	29.154	9.016	184.940	0.012	0.405	
	b	Average Paddle Movement - Paddle&Ball Position Input		A2C	DQN	78719.250	83859.000	-5139.750	4264.838	-1.205	0.722	-0.267	Tukey's
				A2C	HCC	78719.250	52000.427	26718.823	3233.710	8.263	0.001	1.397	
				A2C	MCC	78719.250	50007.504	28711.746	3589.396	7.999	0.001	1.497	
				A2C	PPO	78719.250	75665.500	3053.750	4264.838	0.716	0.900	0.159	
				DQN	HCC	83859.000	52000.427	31858.573	3233.710	9.852	0.001	1.666	
				DQN	MCC	83859.000	50007.504	33851.496	3589.396	9.431	0.001	1.765	
				DQN	PPO	83859.000	75665.500	8193.500	4264.838	1.921	0.307	0.425	
				HCC	MCC	52000.427	50007.504	1992.923	2269.758	0.878	0.900	0.104	
				HCC	PPO	52000.427	75665.500	-23665.073	3233.710	-7.318	0.001	-1.238	
				MCC	PPO	50007.504	75665.500	-25657.996	3589.396	-7.148	0.001	-1.338	
	e	Relative improvement (%) in the average hit counts- Paddle&Ball Position Input		A2C	DQN	21.717	36.623	-14.906	10.286	245.447	0.584	-0.167	Games Howell
				A2C	HCC	21.717	82.147	-60.429	9.165	303.151	0.001	-0.733	
				A2C	MCC	21.717	50.755	-29.038	9.381	203.860	0.019	-0.387	
				A2C	PPO	21.717	14.690	7.027	7.082	292.773	0.842	0.114	
				DQN	HCC	36.623	82.147	-45.523	11.531	304.565	0.001	-0.439	
				DQN	MCC	36.623	50.755	-14.132	11.703	257.834	0.720	-0.151	
				DQN	PPO	36.623	14.690	21.933	9.955	226.546	0.182	0.254	
				HCC	MCC	82.147	50.755	31.391	10.731	262.994	0.030	0.355	
				HCC	PPO	82.147	14.690	67.456	8.792	284.259	0.001	0.853	

				MCC	PPO	50.755	14.690	36.065	9.017	184.981	0.001	0.501	
	c	Average Paddle Movement- Ball Poistion Input		A2C	DQN	67718.750	75019.250	-7300.500	4333.263	-1.685	0.446	-0.373	Tukey's
				A2C	HCC	67718.750	52000.427	15718.323	3285.592	4.784	0.001	0.809	
				A2C	MCC	67718.750	50007.504	17711.246	3646.984	4.856	0.001	0.909	
				A2C	PPO	67718.750	73952.250	-6233.500	4333.263	-1.439	0.589	-0.319	
				DQN	HCC	75019.250	52000.427	23018.823	3285.592	7.006	0.001	1.185	
				DQN	MCC	75019.250	50007.504	25011.746	3646.984	6.858	0.001	1.283	
				DQN	PPO	75019.250	73952.250	1067.000	4333.263	0.246	0.900	0.055	
				HCC	MCC	52000.427	50007.504	1992.923	2306.174	0.864	0.900	0.103	
				HCC	PPO	52000.427	73952.250	-21951.823	3285.592	-6.681	0.001	-1.130	
				MCC	PPO	50007.504	73952.250	-23944.746	3646.984	-6.566	0.001	-1.229	
	f	Relative improvement (%) in the average hit counts- Ball Poistion Input		A2C	DQN	33.724	29.397	4.327	9.789	297.513	0.900	0.051	Games Howell
				A2C	HCC	33.724	82.147	-48.423	10.077	321.871	0.001	-0.534	
				A2C	MCC	33.724	50.755	-17.031	10.274	238.311	0.464	-0.207	
				A2C	PPO	33.724	33.016	0.709	10.301	292.792	0.900	0.008	
				DQN	HCC	29.397	82.147	-52.749	10.268	321.866	0.001	-0.571	
				DQN	MCC	29.397	50.755	-21.358	10.461	243.172	0.249	-0.256	
				DQN	PPO	29.397	33.016	-3.618	10.487	295.423	0.900	-0.040	
				HCC	MCC	82.147	50.755	31.391	10.731	262.994	0.030	0.355	
				HCC	PPO	82.147	33.016	49.131	10.756	317.852	0.001	0.508	
				MCC	PPO	50.755	33.016	17.740	10.941	252.147	0.486	0.203	
**9**	a	Relative improvement (%) in the average hit counts – DQN – varying batch sizes		DQN_16	DQN_32	6.400	43.207	-36.806	17.554	75.733	0.300	-0.416	Games Howell
				DQN_16	DQN_64	6.400	22.119	-15.719	13.194	94.597	0.820	-0.236	
				DQN_16	DQN_8	6.400	12.525	-6.124	12.502	97.075	0.900	-0.097	
				DQN_16	HCC	6.400	82.147	-75.746	11.229	133.216	0.001	-1.079	

				DQN_16	MCC	6.400	50.755	-44.355	11.407	126.260	0.002	-0.660	
				DQN_32	DQN_64	43.207	22.119	21.088	18.470	84.890	0.848	0.227	
				DQN_32	DQN_8	43.207	12.525	30.682	17.983	80.300	0.527	0.339	
				DQN_32	HCC	43.207	82.147	-38.940	17.122	73.462	0.218	-0.364	
				DQN_32	MCC	43.207	50.755	-7.549	17.239	74.550	0.900	-0.074	
				DQN_64	DQN_8	22.119	12.525	9.594	13.759	97.145	0.900	0.138	
				DQN_64	HCC	22.119	82.147	-60.027	12.614	106.983	0.001	-0.761	
				DQN_64	MCC	22.119	50.755	-28.636	12.772	105.863	0.228	-0.381	
				DQN_8	HCC	12.525	82.147	-69.622	11.889	118.917	0.001	-0.936	
				DQN_8	MCC	12.525	50.755	-38.231	12.057	115.635	0.023	-0.538	
				HCC	MCC	82.147	50.755	31.391	10.731	262.994	0.043	0.355	
	b	Relative improvement (%) in the average hit counts – A2C – varying batch sizes		$A2C\_16$	$A2C\_32$	18.203	23.304	-5.101	11.700	97.985	0.900	-0.087	Games Howell
				$A2C\_16$	$A2C\_64$	18.203	23.700	-5.497	13.380	92.457	0.900	-0.082	
				$A2C\_16$	$A2C\_8$	18.203	13.710	4.493	10.929	96.325	0.900	0.082	
				$A2C\_16$	HCC	18.203	82.147	-63.944	11.098	136.551	0.001	-0.921	
				$A2C\_16$	MCC	18.203	50.755	-32.552	11.277	128.559	0.051	-0.490	
				$A2C\_32$	$A2C\_64$	23.304	23.700	-0.396	13.444	92.951	0.900	-0.006	
				$A2C\_32$	$A2C\_8$	23.304	13.710	9.594	11.007	96.006	0.900	0.173	
				$A2C\_32$	HCC	23.304	82.147	-58.843	11.175	134.580	0.001	-0.842	
				$A2C\_32$	MCC	23.304	50.755	-27.452	11.353	127.209	0.158	-0.410	
				$A2C\_64$	$A2C\_8$	23.700	13.710	9.990	12.778	86.482	0.900	0.155	
				$A2C\_64$	HCC	23.700	82.147	-58.446	12.923	102.820	0.001	-0.723	
				$A2C\_64$	MCC	23.700	50.755	-27.055	13.077	102.271	0.312	-0.351	
				$A2C\_8$	HCC	13.710	82.147	-68.436	10.364	158.702	0.001	-1.056	
				$A2C\_8$	MCC	13.710	50.755	-37.045	10.556	142.025	0.008	-0.596	
				HCC	MCC	82.147	50.755	31.391	10.731	262.994	0.043	0.355	

	c	Relative improvement (%) in the average hit counts – PPO – varying batch sizes		PPO_16	PPO_32	24.036	11.686	12.350	14.077	81.194	0.900	0.174	Games Howell
				PPO_16	PPO_64	24.036	-1.291	25.326	14.037	80.683	0.470	0.358	
				PPO_16	PPO_8	24.036	49.866	-25.830	25.190	75.516	0.900	-0.204	
				PPO_16	HCC	24.036	82.147	-58.111	14.132	90.262	0.001	-0.658	
				PPO_16	MCC	24.036	50.755	-26.720	14.274	90.898	0.428	-0.318	
				PPO_32	PPO_64	11.686	-1.291	12.977	10.338	97.989	0.783	0.249	
				PPO_32	PPO_64	11.686	49.866	-38.180	23.333	59.662	0.568	-0.325	
				PPO_32	HCC	11.686	82.147	-70.461	10.468	155.189	0.001	-1.076	
				PPO_32	MCC	11.686	50.755	-39.070	10.658	140.107	0.005	-0.622	
				PPO_64	PPO_8	-1.291	49.866	-51.157	23.308	59.445	0.256	-0.436	
				PPO_64	HCC	-1.291	82.147	-83.437	10.414	157.004	0.001	-1.281	
				PPO_64	MCC	-1.291	50.755	-52.046	10.605	141.109	0.001	-0.833	
				PPO_8	HCC	49.866	82.147	-32.280	23.366	60.514	0.712	-0.221	
				PPO_8	MCC	49.866	50.755	-0.889	23.452	61.224	0.900	-0.006	
				HCC	MCC	82.147	50.755	31.391	10.731	262.994	0.043	0.355	
	d	Relative improvement (%) in the average hit counts – DQN – varying learning rates		$DQN\_0.0001$	$DQN\_0.001$	22.389	7.554	14.834	8.226	1.275	0.770	0.254	Games Howell
				$DQN\_0.0001$	$DQN\_0.002$	22.389	28.761	-6.372	7.688	-0.586	0.900	-0.116	
				$DQN\_0.0001$	$DQN\_0.003$	22.389	37.224	-14.835	8.466	-1.239	0.790	-0.248	
				$DQN\_0.0001$	HCC	22.389	82.147	-46.428	6.473	-5.072	0.001	-0.814	
				$DQN\_0.0001$	MCC	22.389	50.755	-22.400	7.122	-2.224	0.230	-0.379	
				$DQN\_0.001$	$DQN\_0.002$	7.554	28.761	-21.206	8.248	-1.818	0.456	-0.363	
				$DQN\_0.001$	$DQN\_0.003$	7.554	37.224	-29.670	8.978	-2.337	0.183	-0.471	
				$DQN\_0.001$	HCC	7.554	82.147	-61.262	7.129	-6.076	0.001	-0.983	
				$DQN\_0.001$	MCC	7.554	50.755	-37.235	7.723	-3.409	0.010	-0.584	
				$DQN\_0.002$	$DQN\_0.003$	28.761	37.224	-8.463	8.488	-0.705	0.900	-0.141	

				DQN_0.002	HCC	28.761	82.147	-40.056	6.501	-4.357	0.001	-0.699	
				DQN_0.002	MCC	28.761	50.755	-16.028	7.148	-1.586	0.592	-0.270	
				DQN_0.003	HCC	37.224	82.147	-31.592	7.406	-3.017	0.034	-0.492	
				DQN_0.003	MCC	37.224	50.755	-7.565	7.979	-0.670	0.900	-0.116	
				HCC	MCC	82.147	50.755	31.391	7.588	2.925	0.041	0.355	
	e	Relative improvement (%) in the average hit counts – A2C – varying learning rates		$A2C$_0.0001	$A2C$_0.001	23.304	17.936	5.368	8.096	0.469	0.900	0.093	Games Howell
				$A2C$_0.0001	$A2C$_0.002	23.304	25.414	-2.110	7.899	-0.189	0.900	-0.037	
				$A2C$_0.0001	$A2C$_0.003	23.304	12.999	10.305	7.757	0.939	0.900	0.186	
				$A2C$_0.0001	HCC	23.304	82.147	-58.843	7.902	-5.266	0.001	-0.842	
				$A2C$_0.0001	MCC	23.304	50.755	-27.452	8.028	-2.418	0.153	-0.410	
				$A2C$_0.001	$A2C$_0.002	17.936	25.414	-7.478	7.657	-0.691	0.900	-0.137	
				$A2C$_0.001	$A2C$_0.003	17.936	12.999	4.936	7.510	0.465	0.900	0.092	
				$A2C$_0.001	HCC	17.936	82.147	-64.211	7.660	-5.927	0.001	-0.948	
				$A2C$_0.001	MCC	17.936	50.755	-32.820	7.790	-2.979	0.036	-0.506	
				$A2C$_0.002	$A2C$_0.003	25.414	12.999	12.415	7.297	1.203	0.811	0.239	
				$A2C$_0.002	HCC	25.414	82.147	-56.732	7.451	-5.384	0.001	-0.861	
				$A2C$_0.002	MCC	25.414	50.755	-25.341	7.585	-2.362	0.173	-0.401	
				$A2C$_0.003	HCC	12.999	82.147	-69.147	7.301	-6.697	0.001	-1.071	
				$A2C$_0.003	MCC	12.999	50.755	-37.756	7.437	-3.590	0.005	-0.609	
				HCC	MCC	82.147	50.755	31.391	7.588	2.925	0.041	0.355	
	f	Relative improvement (%) in the average hit counts – PPO – varying learning rates		PPO_0.0001	PPO_0.001	11.686	20.406	-8.720	8.975	-0.687	0.900	-0.136	Games Howell
				PPO_0.0001	PPO_0.002	11.686	26.046	-14.360	8.737	-1.162	0.834	-0.231	
				PPO_0.0001	PPO_0.003	11.686	5.590	6.096	8.041	0.536	0.900	0.106	
				PPO_0.0001	HCC	11.686	82.147	70.461	7.402	6.731	0.001	1.076	
				PPO_0.0001	MCC	11.686	50.755	39.070	7.536	3.666	0.004	0.622	
				PPO_0.001	PPO_0.002	20.406	26.046	-5.640	10.143	-0.393	0.900	-0.078	
				PPO_0.001	PPO_0.003	20.406	5.590	14.817	9.550	1.097	0.872	0.218	
				PPO_0.001	HCC	20.406	82.147	61.741	9.018	4.841	0.001	0.774	

1322

				$PPO\_0.001$	MCC	20.406	30.349	9.129	2.351	0.178	0.399	30.349	
				$PPO\_0.002$	$PPO\_0.003$	26.046	5.590	20.456	9.327	1.551	0.612	0.308	
				$PPO\_0.002$	HCC	26.046	82.147	56.101	8.782	4.517	0.001	0.722	
				$PPO\_0.002$	MCC	26.046	50.755	24.710	8.896	1.964	0.366	0.333	
				$PPO\_0.003$	HCC	5.590	82.147	76.557	8.089	6.692	0.001	1.070	
				$PPO\_0.003$	MCC	5.590	50.755	45.166	8.212	3.889	0.002	0.660	
				HCC	MCC	82.147	50.755	31.391	7.588	2.925	0.041	0.355	
**S4**	g	Hit Counts	0-5 Minutes	DQN_16	DQN_32	0.560	0.562	-0.002	0.045	-0.054	0.900	-0.005	Tukey's
				DQN_16	DQN_64	0.560	0.632	-0.072	0.046	-1.567	0.604	-0.150	
				DQN_16	DQN_8	0.560	0.582	-0.022	0.046	-0.472	0.900	-0.045	
				DQN_16	HCC	0.560	0.651	-0.091	0.037	-2.457	0.137	-0.190	
				DQN_16	MCC	0.560	0.716	-0.156	0.039	-3.998	0.001	-0.326	
				DQN_32	DQN_64	0.562	0.632	-0.069	0.044	-1.566	0.605	-0.145	
				DQN_32	DQN_8	0.562	0.582	-0.019	0.045	-0.433	0.900	-0.040	
				DQN_32	HCC	0.562	0.651	-0.088	0.035	-2.522	0.118	-0.184	
				DQN_32	MCC	0.562	0.716	-0.154	0.037	-4.127	0.001	-0.321	
				DQN_64	DQN_8	0.632	0.582	0.050	0.045	1.111	0.867	0.105	
				DQN_64	HCC	0.632	0.651	-0.019	0.036	-0.525	0.900	-0.039	
				DQN_64	MCC	0.632	0.716	-0.084	0.038	-2.220	0.229	-0.176	
				DQN_8	HCC	0.582	0.651	-0.069	0.036	-1.916	0.394	-0.144	
				DQN_8	MCC	0.582	0.716	-0.134	0.038	-3.521	0.006	-0.281	
				HCC	MCC	0.651	0.716	-0.065	0.026	-2.490	0.127	-0.137	
			6-20 Minutes	DQN_16	DQN_32	0.567	0.560	0.007	0.031	0.232	0.900	0.012	
				DQN_16	DQN_64	0.567	0.634	-0.067	0.030	-2.207	0.235	-0.116	
				DQN_16	DQN_8	0.567	0.526	0.041	0.031	1.326	0.743	0.070	
				DQN_16	HCC	0.567	0.854	-0.288	0.025	-11.616	0.001	-0.495	
				DQN_16	MCC	0.567	0.852	-0.286	0.027	-10.708	0.001	-0.492	
				DQN_32	DQN_64	0.560	0.634	-0.074	0.030	-2.442	0.142	-0.128	
				DQN_32	DQN_8	0.560	0.526	0.034	0.031	1.093	0.878	0.058	
				DQN_32	HCC	0.560	0.854	-0.295	0.025	-11.906	0.001	-0.508	
				DQN_32	MCC	0.560	0.852	-0.293	0.027	-10.977	0.001	-0.504	

				DQN_64	DQN_8	0.634	0.526	0.108	0.030	3.555	0.005	0.186	
				DQN_64	HCC	0.634	0.854	-0.220	0.024	-9.087	0.001	-0.380	
				DQN_64	MCC	0.634	0.852	-0.218	0.026	-8.335	0.001	-0.376	
				DQN_8	HCC	0.526	0.854	-0.328	0.025	-13.309	0.001	-0.566	
				DQN_8	MCC	0.526	0.852	-0.326	0.027	-12.274	0.001	-0.562	
				HCC	MCC	0.854	0.852	0.002	0.019	0.103	0.900	0.003	
	h	% Aces	0-5 Minutes	DQN_16	DQN_32	53.103	58.358	-5.255	2.638	-1.992	0.349	-0.395	Tukey's
				DQN_16	DQN_64	53.103	54.163	-1.061	2.638	-0.402	0.900	-0.080	
				DQN_16	DQN_8	53.103	56.084	-2.981	2.638	-1.130	0.856	-0.224	
				DQN_16	HCC	53.103	54.382	-1.279	2.117	-0.604	0.900	-0.097	
				DQN_16	MCC	53.103	53.333	-0.230	2.250	-0.102	0.900	-0.017	
				DQN_32	DQN_64	58.358	54.163	4.194	2.638	1.590	0.591	0.316	
				DQN_32	DQN_8	58.358	56.084	2.274	2.638	0.862	0.900	0.171	
				DQN_32	HCC	58.358	54.382	3.976	2.117	1.879	0.419	0.300	
				DQN_32	MCC	58.358	53.333	5.025	2.250	2.234	0.224	0.379	
				DQN_64	DQN_8	54.163	56.084	-1.921	2.638	-0.728	0.900	-0.145	
				DQN_64	HCC	54.163	54.382	-0.219	2.117	-0.103	0.900	-0.017	
				DQN_64	MCC	54.163	53.333	0.831	2.250	0.369	0.900	0.063	
				DQN_8	HCC	56.084	54.382	1.702	2.117	0.804	0.900	0.129	
				DQN_8	MCC	56.084	53.333	2.751	2.250	1.223	0.802	0.208	
				HCC	MCC	54.382	53.333	1.049	1.607	0.653	0.900	0.079	
			6-20 Minutes	DQN_16	DQN_32	56.069	55.545	0.524	1.744	0.300	0.900	0.060	
				DQN_16	DQN_64	56.069	55.105	0.964	1.744	0.553	0.900	0.110	
				DQN_16	DQN_8	56.069	59.565	-3.496	1.744	-2.004	0.341	-0.398	
				DQN_16	HCC	56.069	49.259	6.810	1.399	4.866	0.001	0.778	
				DQN_16	MCC	56.069	50.232	5.837	1.488	3.924	0.001	0.666	
				DQN_32	DQN_64	55.545	55.105	0.440	1.744	0.252	0.900	0.050	
				DQN_32	DQN_8	55.545	59.565	-4.020	1.744	-2.305	0.194	-0.457	
				DQN_32	HCC	55.545	49.259	6.286	1.399	4.491	0.001	0.718	
				DQN_32	MCC	55.545	50.232	5.313	1.488	3.572	0.005	0.606	
				DQN_64	DQN_8	55.105	59.565	-4.460	1.744	-2.557	0.110	-0.507	

				DQN_64	HCC	55.105	49.259	5.846	1.399	4.177	0.001	0.668	
				DQN_64	MCC	55.105	50.232	4.873	1.488	3.276	0.014	0.556	
				DQN_8	HCC	59.565	49.259	10.306	1.399	7.364	0.001	1.178	
				DQN_8	MCC	59.565	50.232	9.334	1.488	6.274	0.001	1.065	
				HCC	MCC	49.259	50.232	-0.973	1.062	-0.915	0.900	-0.111	
	i	% Long Rally	0-5 Minutes	DQN_16	DQN_32	7.882	4.460	3.422	1.409	2.428	0.148	0.482	Tukey's
				DQN_16	DQN_64	7.882	7.083	0.798	1.409	0.567	0.900	0.112	
				DQN_16	DQN_8	7.882	6.948	0.933	1.409	0.662	0.900	0.131	
				DQN_16	HCC	7.882	4.523	3.359	1.130	2.972	0.037	0.475	
				DQN_16	MCC	7.882	7.318	0.563	1.202	0.469	0.900	0.080	
				DQN_32	DQN_64	4.460	7.083	-2.623	1.409	-1.862	0.429	-0.370	
				DQN_32	DQN_8	4.460	6.948	-2.488	1.409	-1.766	0.489	-0.350	
				DQN_32	HCC	4.460	4.523	-0.063	1.130	-0.055	0.900	-0.009	
				DQN_32	MCC	4.460	7.318	-2.858	1.202	-2.379	0.166	-0.404	
				DQN_64	DQN_8	7.083	6.948	0.135	1.409	0.096	0.900	0.019	
				DQN_64	HCC	7.083	4.523	2.561	1.130	2.265	0.210	0.362	
				DQN_64	MCC	7.083	7.318	-0.235	1.202	-0.195	0.900	-0.033	
				DQN_8	HCC	6.948	4.523	2.426	1.130	2.146	0.266	0.343	
				DQN_8	MCC	6.948	7.318	-0.370	1.202	-0.308	0.900	-0.052	
				HCC	MCC	4.523	7.318	-2.796	0.858	-3.258	0.015	-0.396	
			6-20 Minutes	DQN_16	DQN_32	6.138	6.310	-0.172	0.929	-0.185	0.900	-0.037	
				DQN_16	DQN_64	6.138	6.916	-0.777	0.929	-0.837	0.900	-0.166	
				DQN_16	DQN_8	6.138	6.221	-0.083	0.929	-0.089	0.900	-0.018	
				DQN_16	HCC	6.138	10.365	-4.226	0.746	-5.668	0.001	-0.906	
				DQN_16	MCC	6.138	11.972	-5.834	0.793	-7.361	0.001	-1.250	
				DQN_32	DQN_64	6.310	6.916	-0.605	0.929	-0.651	0.900	-0.129	
				DQN_32	DQN_8	6.310	6.221	0.090	0.929	0.096	0.900	0.019	
				DQN_32	HCC	6.310	10.365	-4.054	0.746	-5.438	0.001	-0.870	
				DQN_32	MCC	6.310	11.972	-5.662	0.793	-7.144	0.001	-1.213	
				DQN_64	DQN_8	6.916	6.221	0.695	0.929	0.748	0.900	0.148	
				DQN_64	HCC	6.916	10.365	-3.449	0.746	-4.626	0.001	-0.740	

				DQN_64	MCC	6.916	11.972	-5.057	0.793	-6.380	0.001	-1.083	
				DQN_8	HCC	6.221	10.365	-4.144	0.746	-5.558	0.001	-0.889	
				DQN_8	MCC	6.221	11.972	-5.751	0.793	-7.257	0.001	-1.232	
**S5**	g	Hit Counts	0-5 Minutes	A2C_16	A2C_32	0.629	0.730	-0.101	0.044	-2.308	0.191	-0.214	Tukey's
				A2C_16	A2C_64	0.629	0.638	-0.009	0.044	-0.208	0.900	-0.019	
				A2C_16	A2C_8	0.629	0.655	-0.026	0.044	-0.594	0.900	-0.056	
				A2C_16	HCC	0.629	0.651	-0.021	0.035	-0.611	0.900	-0.045	
				A2C_16	MCC	0.629	0.716	-0.087	0.037	-2.326	0.184	-0.184	
				A2C_32	A2C_64	0.730	0.638	0.092	0.043	2.115	0.280	0.195	
				A2C_32	A2C_8	0.730	0.655	0.075	0.044	1.710	0.521	0.158	
				A2C_32	HCC	0.730	0.651	0.080	0.035	2.302	0.193	0.169	
				A2C_32	MCC	0.730	0.716	0.014	0.037	0.386	0.900	0.030	
				A2C_64	A2C_8	0.638	0.655	-0.017	0.044	-0.390	0.900	-0.036	
				A2C_64	HCC	0.638	0.651	-0.012	0.035	-0.355	0.900	-0.026	
				A2C_64	MCC	0.638	0.716	-0.078	0.037	-2.104	0.286	-0.165	
				A2C_8	HCC	0.655	0.651	0.005	0.035	0.136	0.900	0.010	
				A2C_8	MCC	0.655	0.716	-0.061	0.037	-1.626	0.570	-0.128	
				HCC	MCC	0.651	0.716	-0.065	0.026	-2.523	0.118	-0.139	
			6-20 Minutes	A2C_16	A2C_32	0.605	0.705	-0.100	0.031	-3.254	0.015	-0.173	
				A2C_16	A2C_64	0.605	0.622	-0.017	0.031	-0.541	0.900	-0.029	
				A2C_16	A2C_8	0.605	0.597	0.008	0.031	0.256	0.900	0.014	
				A2C_16	HCC	0.605	0.854	-0.250	0.025	-9.962	0.001	-0.430	
				A2C_16	MCC	0.605	0.852	-0.248	0.027	-9.188	0.001	-0.426	
				A2C_32	A2C_64	0.705	0.622	0.083	0.031	2.735	0.069	0.144	
				A2C_32	A2C_8	0.705	0.597	0.108	0.031	3.526	0.006	0.186	
				A2C_32	HCC	0.705	0.854	-0.149	0.024	-6.122	0.001	-0.257	
				A2C_32	MCC	0.705	0.852	-0.147	0.026	-5.595	0.001	-0.254	
				A2C_64	A2C_8	0.622	0.597	0.025	0.031	0.801	0.900	0.043	
				A2C_64	HCC	0.622	0.854	-0.233	0.025	-9.427	0.001	-0.401	
				A2C_64	MCC	0.622	0.852	-0.231	0.027	-8.673	0.001	-0.397	

				A2C_8	HCC	0.597	0.854	-0.258	0.025	-10.339	0.001	-0.444	
				A2C_8	MCC	0.597	0.852	-0.256	0.027	-9.531	0.001	-0.440	
				HCC	MCC	0.854	0.852	0.002	0.019	0.103	0.900	0.003	
	h	% Aces	0-5 Minutes	A2C_16	A2C_32	53.810	54.083	-0.272	2.370	-0.115	0.900	-0.023	Tukey's
				A2C_16	A2C_64	53.810	53.299	0.511	2.370	0.216	0.900	0.043	
				A2C_16	A2C_8	53.810	52.332	1.478	2.370	0.624	0.900	0.124	
				A2C_16	HCC	53.810	54.382	-0.571	1.901	-0.301	0.900	-0.048	
				A2C_16	MCC	53.810	53.333	0.478	2.021	0.236	0.900	0.040	
				A2C_32	A2C_64	54.083	53.299	0.783	2.370	0.331	0.900	0.066	
				A2C_32	A2C_8	54.083	52.332	1.750	2.370	0.738	0.900	0.147	
				A2C_32	HCC	54.083	54.382	-0.299	1.901	-0.157	0.900	-0.025	
				A2C_32	MCC	54.083	53.333	0.750	2.021	0.371	0.900	0.063	
				A2C_64	A2C_8	53.299	52.332	0.967	2.370	0.408	0.900	0.081	
				A2C_64	HCC	53.299	54.382	-1.083	1.901	-0.569	0.900	-0.091	
				A2C_64	MCC	53.299	53.333	-0.034	2.021	-0.017	0.900	-0.003	
				A2C_8	HCC	52.332	54.382	-2.049	1.901	-1.078	0.886	-0.172	
				A2C_8	MCC	52.332	53.333	-1.000	2.021	-0.495	0.900	-0.084	
				HCC	MCC	54.382	53.333	1.049	1.443	0.727	0.900	0.088	
			6-20 Minutes	A2C_16	A2C_32	53.076	52.170	0.906	1.627	0.557	0.900	0.110	
				A2C_16	A2C_64	53.076	52.981	0.094	1.627	0.058	0.900	0.012	
				A2C_16	A2C_8	53.076	54.117	-1.042	1.627	-0.640	0.900	-0.127	
				A2C_16	HCC	53.076	49.259	3.816	1.305	2.924	0.042	0.468	
				A2C_16	MCC	53.076	50.232	2.844	1.388	2.049	0.316	0.348	
				A2C_32	A2C_64	52.170	52.981	-0.811	1.627	-0.499	0.900	-0.099	
				A2C_32	A2C_8	52.170	54.117	-1.947	1.627	-1.197	0.817	-0.238	
				A2C_32	HCC	52.170	49.259	2.911	1.305	2.230	0.226	0.357	
				A2C_32	MCC	52.170	50.232	1.938	1.388	1.397	0.702	0.237	
				A2C_64	A2C_8	52.981	54.117	-1.136	1.627	-0.698	0.900	-0.139	
				A2C_64	HCC	52.981	49.259	3.722	1.305	2.851	0.051	0.456	
				A2C_64	MCC	52.981	50.232	2.749	1.388	1.981	0.355	0.336	
				A2C_8	HCC	54.117	49.259	4.858	1.305	3.721	0.003	0.595	
				A2C_8	MCC	54.117	50.232	3.885	1.388	2.800	0.059	0.475	
				HCC	MCC	49.259	50.232	-0.973	0.991	-0.981	0.900	-0.119	
	i	% Long Rally		A2C_16	A2C_32	5.395	6.147	-0.752	1.314	-0.572	0.900	-0.114	Tukey's

			0-5 Minutes	A2C_16	A2C_64	5.395	6.973	-1.578	1.314	-1.201	0.815	-0.238	
				A2C_16	A2C_8	5.395	5.357	0.038	1.314	0.029	0.900	0.006	
				A2C_16	HCC	5.395	4.523	0.872	1.054	0.828	0.900	0.132	
				A2C_16	MCC	5.395	7.318	-1.923	1.120	-1.717	0.518	-0.291	
				A2C_32	A2C_64	6.147	6.973	-0.826	1.314	-0.629	0.900	-0.125	
				A2C_32	A2C_8	6.147	5.357	0.790	1.314	0.602	0.900	0.119	
				A2C_32	HCC	6.147	4.523	1.624	1.054	1.541	0.619	0.246	
				A2C_32	MCC	6.147	7.318	-1.171	1.120	-1.045	0.900	-0.177	
				A2C_64	A2C_8	6.973	5.357	1.617	1.314	1.231	0.798	0.244	
				A2C_64	HCC	6.973	4.523	2.451	1.054	2.325	0.186	0.372	
				A2C_64	MCC	6.973	7.318	-0.345	1.120	-0.308	0.900	-0.052	
				A2C_8	HCC	5.357	4.523	0.834	1.054	0.791	0.900	0.127	
				A2C_8	MCC	5.357	7.318	-1.962	1.120	-1.751	0.498	-0.297	
				HCC	MCC	4.523	7.318	-2.796	0.800	-3.494	0.007	-0.424	
			6-20 Minutes	A2C_16	A2C_32	6.077	7.705	-1.628	0.942	-1.727	0.512	-0.343	
				A2C_16	A2C_64	6.077	6.942	-0.865	0.942	-0.917	0.900	-0.182	
				A2C_16	A2C_8	6.077	6.231	-0.154	0.942	-0.163	0.900	-0.032	
				A2C_16	HCC	6.077	10.365	-4.287	0.756	-5.671	0.001	-0.907	
				A2C_16	MCC	6.077	11.972	-5.895	0.804	-7.336	0.001	-1.245	
				A2C_32	A2C_64	7.705	6.942	0.763	0.942	0.810	0.900	0.161	
				A2C_32	A2C_8	7.705	6.231	1.474	0.942	1.564	0.606	0.310	
				A2C_32	HCC	7.705	10.365	-2.660	0.756	-3.518	0.006	-0.563	
				A2C_32	MCC	7.705	11.972	-4.267	0.804	-5.310	0.001	-0.901	
				A2C_64	A2C_8	6.942	6.231	0.711	0.942	0.754	0.900	0.150	
				A2C_64	HCC	6.942	10.365	-3.423	0.756	-4.528	0.001	-0.724	
				A2C_64	MCC	6.942	11.972	-5.030	0.804	-6.260	0.001	-1.063	
				A2C_8	HCC	6.231	10.365	-4.134	0.756	-5.468	0.001	-0.874	
				A2C_8	MCC	6.231	11.972	-5.741	0.804	-7.144	0.001	-1.213	
				HCC	MCC	10.365	11.972	-1.608	0.574	-2.801	0.059	-0.340	
**S6**	g	Hit Counts	0-5 Minutes	PPO_16	PPO_32	0.557	0.555	0.002	0.044	0.035	0.900	0.003	Tukey's
				PPO_16	PPO_64	0.557	0.632	-0.075	0.044	-1.684	0.537	-0.159	

				PPO_16	PPO_8	0.557	0.552	0.005	0.045	0.121	0.900	0.011	
				PPO_16	HCC	0.557	0.651	-0.094	0.035	-2.639	0.088	-0.199	
				PPO_16	MCC	0.557	0.716	-0.159	0.038	-4.227	0.001	-0.338	
				PPO_32	PPO_64	0.555	0.632	-0.076	0.044	-1.734	0.508	-0.162	
				PPO_32	PPO_64	0.555	0.552	0.004	0.044	0.088	0.900	0.008	
				PPO_32	HCC	0.555	0.651	-0.095	0.035	-2.720	0.072	-0.202	
				PPO_32	MCC	0.555	0.716	-0.161	0.037	-4.322	0.001	-0.341	
				PPO_64	PPO_8	0.632	0.552	0.080	0.044	1.816	0.457	0.171	
				PPO_64	HCC	0.632	0.651	-0.019	0.035	-0.536	0.900	-0.040	
				PPO_64	MCC	0.632	0.716	-0.084	0.037	-2.262	0.210	-0.179	
				PPO_8	HCC	0.552	0.651	-0.099	0.035	-2.816	0.055	-0.211	
				PPO_8	MCC	0.552	0.716	-0.164	0.037	-4.405	0.001	-0.350	
				HCC	MCC	0.651	0.716	-0.065	0.026	-2.532	0.115	-0.139	
			6-20 Minutes	PPO_16	PPO_32	0.508	0.523	-0.015	0.030	-0.486	0.900	-0.026	
				PPO_16	PPO_64	0.508	0.513	-0.005	0.030	-0.160	0.900	-0.008	
				PPO_16	PPO_8	0.508	0.556	-0.048	0.030	-1.579	0.597	-0.083	
				PPO_16	HCC	0.508	0.854	-0.347	0.024	-14.206	0.001	-0.607	
				PPO_16	MCC	0.508	0.852	-0.345	0.026	-13.114	0.001	-0.603	
				PPO_32	PPO_64	0.523	0.513	0.010	0.030	0.328	0.900	0.017	
				PPO_32	PPO_64	0.523	0.556	-0.033	0.030	-1.090	0.880	-0.058	
				PPO_32	HCC	0.523	0.854	-0.332	0.024	-13.615	0.001	-0.581	
				PPO_32	MCC	0.523	0.852	-0.330	0.026	-12.563	0.001	-0.577	
				PPO_64	PPO_8	0.513	0.556	-0.043	0.030	-1.424	0.687	-0.075	
				PPO_64	HCC	0.513	0.854	-0.342	0.024	-14.092	0.001	-0.598	
				PPO_64	MCC	0.513	0.852	-0.340	0.026	-12.997	0.001	-0.595	
				PPO_8	HCC	0.556	0.854	-0.299	0.024	-12.406	0.001	-0.523	
				PPO_8	MCC	0.556	0.852	-0.297	0.026	-11.422	0.001	-0.520	

1329

				HCC	MCC	0.854	0.852	0.002	0.019	0.104	0.900	0.003	
	h	% Aces	0-5 Minutes	PPO_16	PPO_32	59.143	59.654	-0.511	2.553	-0.200	0.900	-0.040	Tukey's
				PPO_16	PPO_64	59.143	55.534	3.610	2.553	1.414	0.692	0.281	
				PPO_16	PPO_8	59.143	58.904	0.239	2.553	0.094	0.900	0.019	
				PPO_16	HCC	59.143	54.382	4.762	2.048	2.325	0.186	0.372	
				PPO_16	MCC	59.143	53.333	5.811	2.177	2.669	0.084	0.453	
				PPO_32	PPO_64	59.654	55.534	4.120	2.553	1.614	0.577	0.320	
				PPO_32	PPO_64	59.654	58.904	0.750	2.553	0.294	0.900	0.058	
				PPO_32	HCC	59.654	54.382	5.273	2.048	2.575	0.106	0.412	
				PPO_32	MCC	59.654	53.333	6.322	2.177	2.904	0.044	0.493	
				PPO_64	PPO_8	55.534	58.904	-3.370	2.553	-1.320	0.746	-0.262	
				PPO_64	HCC	55.534	54.382	1.152	2.048	0.563	0.900	0.090	
				PPO_64	MCC	55.534	53.333	2.201	2.177	1.011	0.900	0.172	
				PPO_8	HCC	58.904	54.382	4.522	2.048	2.208	0.236	0.353	
				PPO_8	MCC	58.904	53.333	5.571	2.177	2.559	0.110	0.434	
				HCC	MCC	54.382	53.333	1.049	1.555	0.675	0.900	0.082	
			6-20 Minutes	PPO_16	PPO_32	60.504	60.595	-0.091	1.900	-0.048	0.900	-0.010	
				PPO_16	PPO_64	60.504	60.941	-0.438	1.900	-0.230	0.900	-0.046	
				PPO_16	PPO_8	60.504	58.316	2.187	1.900	1.151	0.844	0.228	
				PPO_16	HCC	60.504	49.259	11.244	1.524	7.376	0.001	1.180	
				PPO_16	MCC	60.504	50.232	10.272	1.620	6.339	0.001	1.076	
				PPO_32	PPO_64	60.595	60.941	-0.347	1.900	-0.182	0.900	-0.036	
				PPO_32	PPO_64	60.595	58.316	2.279	1.900	1.199	0.816	0.238	
				PPO_32	HCC	60.595	49.259	11.335	1.524	7.436	0.001	1.189	
				PPO_32	MCC	60.595	50.232	10.363	1.620	6.395	0.001	1.086	
				PPO_64	PPO_8	60.941	58.316	2.625	1.900	1.382	0.711	0.274	
				PPO_64	HCC	60.941	49.259	11.682	1.524	7.663	0.001	1.225	
				PPO_64	MCC	60.941	50.232	10.709	1.620	6.609	0.001	1.122	
				PPO_8	HCC	58.316	49.259	9.057	1.524	5.941	0.001	0.950	
				PPO_8	MCC	58.316	50.232	8.084	1.620	4.989	0.001	0.847	
				HCC	MCC	49.259	50.232	-0.973	1.157	-0.840	0.900	-0.102	

	i	% Long Rally	0-5 Minutes	PPO_16	PPO_32	4.885	5.501	-0.616	1.359	-0.453	0.900	-0.090	Tukey's
				PPO_16	PPO_64	4.885	7.393	-2.508	1.359	-1.845	0.439	-0.366	
				PPO_16	PPO_8	4.885	6.020	-1.135	1.359	-0.835	0.900	-0.166	
				PPO_16	HCC	4.885	4.523	0.362	1.090	0.332	0.900	0.053	
				PPO_16	MCC	4.885	7.318	-2.433	1.159	-2.099	0.290	-0.356	
				PPO_32	PPO_64	5.501	7.393	-1.892	1.359	-1.392	0.705	-0.276	
				PPO_32	PPO_64	5.501	6.020	-0.519	1.359	-0.382	0.900	-0.076	
				PPO_32	HCC	5.501	4.523	0.979	1.090	0.897	0.900	0.144	
				PPO_32	MCC	5.501	7.318	-1.817	1.159	-1.568	0.604	-0.266	
				PPO_64	PPO_8	7.393	6.020	1.373	1.359	1.010	0.900	0.201	
				PPO_64	HCC	7.393	4.523	2.871	1.090	2.633	0.091	0.421	
				PPO_64	MCC	7.393	7.318	0.075	1.159	0.065	0.900	0.011	
				PPO_8	HCC	6.020	4.523	1.497	1.090	1.373	0.716	0.220	
				PPO_8	MCC	6.020	7.318	-1.298	1.159	-1.120	0.862	-0.190	
				HCC	MCC	4.523	7.318	-2.796	0.828	-3.377	0.010	-0.410	
			6-20 Minutes	PPO_16	PPO_32	6.008	5.224	0.784	0.871	0.900	0.900	0.179	
				PPO_16	PPO_64	6.008	5.339	0.669	0.871	0.769	0.900	0.153	
				PPO_16	PPO_8	6.008	6.234	-0.226	0.871	-0.260	0.900	-0.052	
				PPO_16	HCC	6.008	10.365	-4.357	0.699	-6.237	0.001	-0.997	
				PPO_16	MCC	6.008	11.972	-5.964	0.743	-8.032	0.001	-1.363	
				PPO_32	PPO_64	5.224	5.339	-0.114	0.871	-0.131	0.900	-0.026	
				PPO_32	PPO_64	5.224	6.234	-1.010	0.871	-1.160	0.839	-0.230	
				PPO_32	HCC	5.224	10.365	-5.140	0.699	-7.358	0.001	-1.177	
				PPO_32	MCC	5.224	11.972	-6.748	0.743	-9.088	0.001	-1.543	
				PPO_64	PPO_8	5.339	6.234	-0.896	0.871	-1.029	0.900	-0.204	
				PPO_64	HCC	5.339	10.365	-5.026	0.699	-7.195	0.001	-1.151	
				PPO_64	MCC	5.339	11.972	-6.634	0.743	-8.934	0.001	-1.517	
				PPO_8	HCC	6.234	10.365	-4.130	0.699	-5.913	0.001	-0.946	
				PPO_8	MCC	6.234	11.972	-5.738	0.743	-7.728	0.001	-1.312	
				HCC	MCC	10.365	11.972	-1.608	0.530	-3.031	0.031	-0.368	
**S7**	g	Hit Counts	0-5 Minutes	A2C	DQN	0.771	0.717	0.054	0.028	1.937	0.298	0.103	Tukey's
				A2C	HCC	0.771	0.651	0.121	0.027	4.508	0.001	0.229	
				A2C	MCC	0.771	0.716	0.055	0.030	1.827	0.359	0.105	
				A2C	PPO	0.771	0.698	0.073	0.028	2.594	0.072	0.139	

				DQN	HCC	0.717	0.651	0.066	0.027	2.476	0.096	0.126	
				DQN	MCC	0.717	0.716	0.001	0.030	0.025	0.900	0.001	
				DQN	PPO	0.717	0.698	0.019	0.028	0.668	0.900	0.036	
				HCC	MCC	0.651	0.716	-0.065	0.029	-2.263	0.157	-0.124	
				HCC	PPO	0.651	0.698	-0.047	0.027	-1.760	0.399	-0.090	
				MCC	PPO	0.716	0.698	0.018	0.030	0.598	0.900	0.034	
			6-20 Minutes	A2C	DQN	0.777	0.762	0.016	0.018	0.866	0.900	0.026	
				A2C	HCC	0.777	0.854	-0.077	0.018	-4.329	0.001	-0.127	
				A2C	MCC	0.777	0.852	-0.075	0.021	-3.647	0.002	-0.124	
				A2C	PPO	0.777	0.712	0.065	0.018	3.561	0.003	0.108	
				DQN	HCC	0.762	0.854	-0.093	0.018	-5.262	0.001	-0.153	
				DQN	MCC	0.762	0.852	-0.091	0.020	-4.442	0.001	-0.150	
				DQN	PPO	0.762	0.712	0.049	0.018	2.723	0.051	0.082	
				HCC	MCC	0.854	0.852	0.002	0.020	0.099	0.900	0.003	
				HCC	PPO	0.854	0.712	0.142	0.018	8.009	0.001	0.235	
				MCC	PPO	0.852	0.712	0.140	0.021	6.825	0.001	0.232	
	h	% Aces	0-5 Minutes	A2C	DQN	53.293	52.579	0.714	1.455	0.491	0.900	0.057	Tukey's
				A2C	HCC	53.293	54.382	-1.089	1.404	-0.776	0.900	-0.086	
				A2C	MCC	53.293	53.333	-0.040	1.582	-0.025	0.900	-0.003	
				A2C	PPO	53.293	54.248	-0.956	1.455	-0.657	0.900	-0.076	
				DQN	HCC	52.579	54.382	-1.803	1.404	-1.284	0.677	-0.143	
				DQN	MCC	52.579	53.333	-0.754	1.582	-0.477	0.900	-0.060	
				DQN	PPO	52.579	54.248	-1.670	1.455	-1.147	0.754	-0.132	
				HCC	MCC	54.382	53.333	1.049	1.535	0.683	0.900	0.083	
				HCC	PPO	54.382	54.248	0.133	1.404	0.095	0.900	0.011	
				MCC	PPO	53.333	54.248	-0.916	1.582	-0.579	0.900	-0.072	
			6-20 Minutes	A2C	DQN	52.530	49.935	2.595	0.959	2.706	0.054	0.312	
				A2C	HCC	52.530	49.259	3.270	0.925	3.535	0.004	0.393	
				A2C	MCC	52.530	50.232	2.298	1.042	2.205	0.179	0.276	
				A2C	PPO	52.530	52.511	0.018	0.959	0.019	0.900	0.002	
				DQN	HCC	49.935	49.259	0.676	0.925	0.730	0.900	0.081	
				DQN	MCC	49.935	50.232	-0.297	1.042	-0.285	0.900	-0.036	
				DQN	PPO	49.935	52.511	-2.576	0.959	-2.687	0.057	-0.310	

				HCC	MCC	49.259	50.232	-0.973	1.011	-0.962	0.860	-0.117	
				HCC	PPO	49.259	52.511	-3.252	0.925	-3.515	0.004	-0.391	
				MCC	PPO	50.232	52.511	-2.280	1.042	-2.187	0.186	-0.274	
	i	% Long Rally	0-5 Minutes	A2C	DQN	12.722	10.195	2.527	0.631	4.004	0.001	0.461	Tukey's
				A2C	HCC	12.722	10.365	2.357	0.609	3.871	0.001	0.430	
				A2C	MCC	12.722	11.972	0.750	0.686	1.093	0.785	0.137	
				A2C	PPO	12.722	10.183	2.540	0.631	4.024	0.001	0.463	
				DQN	HCC	10.195	10.365	-0.169	0.609	-0.278	0.900	-0.031	
				DQN	MCC	10.195	11.972	-1.777	0.686	-2.590	0.073	-0.324	
				DQN	PPO	10.195	10.183	0.013	0.631	0.020	0.900	0.002	
				HCC	MCC	10.365	11.972	-1.608	0.666	-2.415	0.113	-0.293	
				HCC	PPO	10.365	10.183	0.182	0.609	0.299	0.900	0.033	
				MCC	PPO	11.972	10.183	1.790	0.686	2.609	0.070	0.327	
			6-20 Minutes	A2C	DQN	11.266	9.629	1.637	0.589	2.777	0.044	0.226	
				A2C	HCC	11.266	7.444	3.823	0.569	6.721	0.001	0.529	
				A2C	MCC	11.266	9.645	1.621	0.641	2.530	0.085	0.224	
				A2C	PPO	11.266	9.793	1.474	0.589	2.500	0.091	0.204	
				DQN	HCC	9.629	7.444	2.186	0.569	3.843	0.001	0.302	
				DQN	MCC	9.629	9.645	-0.016	0.641	-0.025	0.900	-0.002	
				DQN	PPO	9.629	9.793	-0.163	0.589	-0.277	0.900	-0.023	
				HCC	MCC	7.444	9.645	-2.202	0.622	-3.540	0.004	-0.305	
				HCC	PPO	7.444	9.793	-2.349	0.569	-4.130	0.001	-0.325	
				MCC	PPO	9.645	9.793	-0.147	0.641	-0.230	0.900	-0.020	
	p	Hit Counts	0-5 Minutes	A2C	DQN	0.722	0.719	0.003	0.027	0.124	0.900	0.007	Tukey's
				A2C	HCC	0.722	0.651	0.072	0.026	2.773	0.044	0.141	
				A2C	MCC	0.722	0.716	0.006	0.029	0.217	0.900	0.012	
				A2C	PPO	0.722	0.740	-0.018	0.027	-0.644	0.900	-0.035	
				DQN	HCC	0.719	0.651	0.068	0.026	2.648	0.062	0.135	
				DQN	MCC	0.719	0.716	0.003	0.029	0.101	0.900	0.006	
				DQN	PPO	0.719	0.740	-0.021	0.027	-0.769	0.900	-0.041	
				HCC	MCC	0.651	0.716	-0.065	0.028	-2.346	0.131	-0.129	
				HCC	PPO	0.651	0.740	-0.089	0.026	-3.444	0.005	-0.176	
				MCC	PPO	0.716	0.740	-0.024	0.029	-0.819	0.900	-0.047	

			6-20 Minutes	A2C	DQN	0.724	0.741	-0.017	0.018	-0.947	0.868	-0.029	
				A2C	HCC	0.724	0.854	-0.131	0.017	-7.488	0.001	-0.220	
				A2C	MCC	0.724	0.852	-0.129	0.020	-6.378	0.001	-0.217	
				A2C	PPO	0.724	0.727	-0.004	0.018	-0.218	0.900	-0.007	
				DQN	HCC	0.741	0.854	-0.114	0.017	-6.567	0.001	-0.192	
				DQN	MCC	0.741	0.852	-0.112	0.020	-5.570	0.001	-0.189	
				DQN	PPO	0.741	0.727	0.013	0.018	0.727	0.900	0.022	
				HCC	MCC	0.854	0.852	0.002	0.020	0.100	0.900	0.003	
				HCC	PPO	0.854	0.727	0.127	0.017	7.259	0.001	0.214	
				MCC	PPO	0.852	0.727	0.125	0.020	6.181	0.001	0.211	
	q	% Aces	0-5 Minutes	A2C	DQN	51.318	53.675	-2.356	1.437	-1.640	0.473	-0.189	Tukey's
				A2C	HCC	51.318	54.382	-3.064	1.387	-2.209	0.177	-0.246	
				A2C	MCC	51.318	53.333	-2.014	1.562	-1.289	0.674	-0.161	
				A2C	PPO	51.318	50.866	0.453	1.437	0.315	0.900	0.036	
				DQN	HCC	53.675	54.382	-0.707	1.387	-0.510	0.900	-0.057	
				DQN	MCC	53.675	53.333	0.342	1.562	0.219	0.900	0.027	
				DQN	PPO	53.675	50.866	2.809	1.437	1.955	0.290	0.225	
				HCC	MCC	54.382	53.333	1.049	1.516	0.692	0.900	0.084	
				HCC	PPO	54.382	50.866	3.516	1.387	2.536	0.084	0.282	
				MCC	PPO	53.333	50.866	2.467	1.562	1.579	0.510	0.198	
			6-20 Minutes	A2C	DQN	52.596	51.199	1.397	0.907	1.540	0.532	0.177	
				A2C	HCC	52.596	49.259	3.337	0.875	3.813	0.001	0.424	
				A2C	MCC	52.596	50.232	2.364	0.986	2.398	0.117	0.300	
				A2C	PPO	52.596	51.658	0.938	0.907	1.034	0.818	0.119	
				DQN	HCC	51.199	49.259	1.940	0.875	2.217	0.175	0.246	
				DQN	MCC	51.199	50.232	0.968	0.986	0.981	0.848	0.123	
				DQN	PPO	51.199	51.658	-0.459	0.907	-0.506	0.900	-0.058	
				HCC	MCC	49.259	50.232	-0.973	0.957	-1.017	0.828	-0.124	
				HCC	PPO	49.259	51.658	-2.399	0.875	-2.742	0.049	-0.305	
				MCC	PPO	50.232	51.658	-1.427	0.986	-1.447	0.584	-0.181	
	r	% Long Rally	0-5 Minutes	A2C	DQN	9.519	9.710	-0.191	0.965	-0.198	0.900	-0.023	Tukey's
				A2C	HCC	9.519	4.523	4.997	0.931	5.366	0.001	0.596	
				A2C	MCC	9.519	7.318	2.201	1.049	2.098	0.222	0.263	
				A2C	PPO	9.519	10.462	-0.942	0.965	-0.976	0.851	-0.112	

				DQN	HCC	9.710	4.523	5.188	0.931	5.571	0.001	0.619	
				DQN	MCC	9.710	7.318	2.392	1.049	2.280	0.153	0.285	
				DQN	PPO	9.710	10.462	-0.752	0.965	-0.779	0.900	-0.090	
				HCC	MCC	4.523	7.318	-2.796	1.018	-2.746	0.048	-0.334	
				HCC	PPO	4.523	10.462	-5.939	0.931	-6.378	0.001	-0.709	
				MCC	PPO	7.318	10.462	-3.144	1.049	-2.996	0.024	-0.375	
			6-20 Minutes	A2C	DQN	10.431	10.187	0.244	0.591	0.413	0.900	0.048	
				A2C	HCC	10.431	10.365	0.066	0.570	0.116	0.900	0.013	
				A2C	MCC	10.431	11.972	-1.541	0.642	-2.400	0.117	-0.300	
				A2C	PPO	10.431	10.049	0.382	0.591	0.646	0.900	0.074	
				DQN	HCC	10.187	10.365	-0.178	0.570	-0.312	0.900	-0.035	
				DQN	MCC	10.187	11.972	-1.785	0.642	-2.780	0.044	-0.348	
				DQN	PPO	10.187	10.049	0.138	0.591	0.233	0.900	0.027	
				HCC	MCC	10.365	11.972	-1.608	0.623	-2.579	0.075	-0.313	
				HCC	PPO	10.365	10.049	0.316	0.570	0.554	0.900	0.062	
				MCC	PPO	11.972	10.049	1.923	0.642	2.994	0.024	0.375	
**S8**	a	Relative improvement (%) in the average hit counts – Ball Position Input		A2C	DQN	33.724	28.251	5.473	8.669	283.806	0.900	0.073	Games Howell
				A2C	HCC	33.724	82.147	-48.423	10.077	321.871	0.001	-0.534	
				A2C	MCC	33.724	50.755	-17.031	10.274	238.311	0.464	-0.207	
				A2C	PPO	33.724	33.016	0.709	10.301	292.792	0.900	0.008	
				DQN	HCC	28.251	82.147	-53.896	9.206	304.784	0.001	-0.651	
				DQN	MCC	28.251	50.755	-22.505	9.421	205.771	0.123	-0.299	
				DQN	PPO	28.251	33.016	-4.765	9.450	266.029	0.900	-0.058	
				HCC	MCC	82.147	50.755	31.391	10.731	262.994	0.030	0.355	
				HCC	PPO	82.147	33.016	49.131	10.756	317.852	0.001	0.508	
				MCC	PPO	50.755	33.016	17.740	10.941	252.147	0.486	0.203	
	b	Relative improvement (%) in the average hit counts – Paddle&Ball Position Input		A2C	DQN	21.717	24.949	-3.232	8.194	291.151	0.900	-0.045	Games Howell
				A2C	HCC	21.717	82.147	-60.429	9.165	303.151	0.001	-0.733	
				A2C	MCC	21.717	50.755	-29.038	9.381	203.860	0.019	-0.387	
				A2C	PPO	21.717	14.690	7.027	7.082	292.773	0.842	0.114	

				DQN	HCC	24.949	82.147	-57.197	9.711	318.526	0.001	-0.655	
				DQN	MCC	24.949	50.755	-25.806	9.915	226.675	0.073	-0.326	
				DQN	PPO	24.949	14.690	10.259	7.775	276.159	0.657	0.152	
				HCC	MCC	82.147	50.755	31.391	10.731	262.994	0.030	0.355	
				HCC	PPO	82.147	14.690	67.456	8.792	284.259	0.001	0.853	
				MCC	PPO	50.755	14.690	36.065	9.017	184.981	0.001	0.501	
**S9**	e	Hit Counts	0-5 Minutes	CL(3)	CL(7)	0.696	0.682	0.014	0.050	0.281	0.900	0.027	Tuckey's
				CL(3)	HCC	0.696	0.651	0.045	0.039	1.147	0.641	0.088	
				CL(3)	MCC	0.696	0.716	-0.020	0.042	-0.484	0.900	-0.039	
				CL(7)	HCC	0.682	0.651	0.031	0.039	0.804	0.834	0.061	
				CL(7)	MCC	0.682	0.716	-0.034	0.041	-0.827	0.821	-0.066	
				HCC	MCC	0.651	0.716	-0.065	0.028	-2.318	0.094	-0.127	
			6-20 Minutes	CL(3)	CL(7)	0.703	0.916	-0.213	0.039	-5.439	0.001	-0.336	
				CL(3)	HCC	0.703	0.854	-0.151	0.030	-4.972	0.001	-0.239	
				CL(3)	MCC	0.703	0.852	-0.149	0.032	-4.624	0.001	-0.236	
				CL(7)	HCC	0.916	0.854	0.061	0.030	2.017	0.182	0.097	
				CL(7)	MCC	0.916	0.852	0.063	0.032	1.962	0.203	0.100	
				HCC	MCC	0.854	0.852	0.002	0.021	0.094	0.900	0.003	
	f	%Aces	0-5 Minutes	CL(3)	CL(7)	53.140	54.239	-1.099	2.560	-0.429	0.900	-0.095	
				CL(3)	HCC	53.140	54.382	-1.242	2.008	-0.618	0.900	-0.108	
				CL(3)	MCC	53.140	53.333	-0.192	2.114	-0.091	0.900	-0.017	
				CL(7)	HCC	54.239	54.382	-0.143	2.008	-0.071	0.900	-0.012	
				CL(7)	MCC	54.239	53.333	0.906	2.114	0.429	0.900	0.079	
				HCC	MCC	54.382	53.333	1.049	1.395	0.752	0.863	0.091	
			6-20 Minutes	CL(3)	CL(7)	55.605	47.256	8.349	1.712	4.876	0.001	1.080	
				CL(3)	HCC	55.605	49.259	6.346	1.343	4.726	0.001	0.826	
				CL(3)	MCC	55.605	50.232	5.373	1.414	3.800	0.001	0.698	
				CL(7)	HCC	47.256	49.259	-2.003	1.343	-1.492	0.445	-0.261	
				CL(7)	MCC	47.256	50.232	-2.976	1.414	-2.105	0.154	-0.387	
				HCC	MCC	49.259	50.232	-0.973	0.933	-1.043	0.700	-0.127	
	g	%Long Rally	0-5 Minutes	CL(3)	CL(7)	7.692	6.923	0.769	1.432	0.537	0.900	0.119	
				CL(3)	HCC	7.692	4.523	3.170	1.123	2.822	0.026	0.493	
				CL(3)	MCC	7.692	7.318	0.374	1.183	0.316	0.900	0.058	

				CL(7)	HCC	6.923	4.523	2.401	1.123	2.138	0.143	0.373	
				CL(7)	MCC	6.923	7.318	-0.395	1.183	-0.334	0.900	-0.061	
				HCC	MCC	4.523	7.318	-2.796	0.780	-3.583	0.002	-0.435	
			6-20 Minutes	CL(3)	CL(7)	9.292	12.160	-2.868	1.111	-2.582	0.050	-0.572	
				CL(3)	HCC	9.292	10.365	-1.073	0.871	-1.232	0.594	-0.215	
				CL(3)	MCC	9.292	11.972	-2.680	0.917	-2.923	0.019	-0.537	
				CL(7)	HCC	12.160	10.365	1.795	0.871	2.061	0.168	0.360	
				CL(7)	MCC	12.160	11.972	0.187	0.917	0.204	0.900	0.038	
				HCC	MCC	10.365	11.972	-1.608	0.605	-2.657	0.041	-0.323	
	h	Relative improvement (%) in the average hit counts – Active Inference		CL(3)	CL(7)	20.341	54.109	-33.768	15.953	-2.117	0.157	-0.469	Games Howell
				CL(3)	HCC	20.341	82.147	-61.806	14.023	-4.407	0.001	-0.770	
				CL(3)	MCC	20.341	50.755	-30.414	14.165	-2.147	0.148	-0.394	
				CL(7)	HCC	54.109	82.147	-28.038	13.000	-2.157	0.144	-0.377	
				CL(7)	MCC	54.109	50.755	3.353	13.154	0.255	0.900	0.047	
				HCC	MCC	82.147	50.755	31.391	10.731	2.925	0.019	0.355	

Table S3. Multivariate statistical tests and all results for tests done.

Figure	Panel	Parameters	Source	DF1	DF2	MS	F	p-value	np2	Method
**5**	e	Average Rally Length	Group - all	4	729	0.185	1.021	0.395	0.006	Mixed ANOVA
			Time Interval - all	1	729	2.134	-21.944	1.000	-0.031	
			Interaction - all	4	729	0.575	-5.909	1.000	-0.034	
	f	% Aces	Group - all	4	729	0.044	1.014	0.399	0.006	Mixed ANOVA
			Time Interval - all	1	729	0.124	-5.589	1.000	-0.008	
			Interaction - all	4	729	0.015	-0.685	1.000	-0.004	
	g	% Long Rally	Group - all	4	729	0.019	1.749	0.137	0.010	Mixed ANOVA
			Time Interval - all	1	729	0.063	-11.125	1.000	-0.015	
			Interaction - all	4	729	0.039	-6.931	1.000	-0.040	
**6**	e	Average Rally Length	Group - all	4	729	0.170	0.926	0.448	0.005	Mixed ANOVA
			Time Intervals - all	1	729	1.488	-15.161	1.000	-0.021	
			Interaction - all	4	729	0.704	-7.170	1.000	-0.041	
	f	% Aces	Group - all	4	729	0.061	1.332	0.256	0.007	Mixed ANOVA
			Time Intervals - all	1	729	0.022	-0.957	1.000	-0.001	
			Interaction - all	4	729	0.041	-1.745	1.000	-0.010	
	g	% Long Rally	Group - all	4	729	0.011	0.886	0.472	0.005	

			Time Intervals - all	1	729	0.073	-11.249	1.000	-0.016	Mixed ANOVA
			Interaction - all	4	729	0.033	-5.038	1.000	-0.028	
**7**	e	Average Rally Length	Group - all	4	729	0.499	2.589	0.036	0.014	Mixed ANOVA
			Time Intervals- all	1	729	1.934	-18.645	1.000	-0.026	
			Interaction - all	4	729	0.599	-5.774	1.000	-0.033	
	f	% Aces	Group - all	4	729	0.111	2.331	0.055	0.013	Mixed ANOVA
			Time Intervals- all	1	729	0.111	-4.583	1.000	-0.006	
			Interaction - all	4	729	0.021	-0.871	1.000	-0.005	
	g	% Long Rally	Group - all	4	729	0.018	1.523	0.194	0.008	Mixed ANOVA
			Time Intervals - all	1	729	0.081	-12.847	1.000	-0.018	
			Interaction - all	4	729	0.032	-5.057	1.000	-0.029	
**S4**	d	Average Rally Length	Group - all	5	478	1.645	8.293	0.0	0.080	Mixed ANOVA
			Time Intervals - all	1	478	2.153	-18.414	1.0	-0.040	
			Interaction - all	5	478	0.443	-3.787	1.0	-0.041	
	e	% Aces	Group - all	5	478	0.259	5.194	0.0	0.052	Mixed ANOVA
			Time Intervals - all	1	478	0.059	-2.212	1.0	-0.005	
			Interaction - all	5	478	0.027	-1.008	1.0	-0.011	
	f	% Long Rally	Group - all	5	478	0.049	4.611	0.0	0.046	Mixed ANOVA
			Time Intervals - all	1	478	0.113	-18.808	1.0	-0.041	
			Interaction - all	5	478	0.019	-3.197	1.0	-0.035	
**S5**	d	Average Rally Length	Group - all	5	478	0.765	4.206	0.001	0.042	Mixed ANOVA
			Time Intervals - all	1	478	1.873	-17.980	1.000	-0.039	
			Interaction - all	5	478	0.502	-4.819	1.000	-0.053	
	e	% Aces	Group - all	5	478	0.060	1.410	0.219	0.015	Mixed ANOVA
			Time Intervals - all	1	478	0.050	-2.277	1.000	-0.005	
			Interaction - all	5	478	0.029	-1.306	1.000	-0.014	
	f	% Long Rally	Group - all	5	478	0.032	2.926	0.013	0.030	Mixed ANOVA
			Time Intervals - all	1	478	0.081	-13.550	1.000	-0.029	
			Interaction - all	5	478	0.026	-4.281	1.000	-0.047	
**S6**	d	Average Rally Length	Group - all	5	478	2.177	10.721	0.0	0.101	Mixed ANOVA
			Time Intervals - all	1	478	1.503	-12.236	1.0	-0.026	
			Interaction - all	5	478	0.645	-5.254	1.0	-0.058	
	e	% Aces	Group - all	5	478	0.421	7.738	0.0	0.075	Mixed ANOVA
			Time Intervals - all	1	478	0.029	-0.970	1.0	-0.002	
			Interaction - all	5	478	0.046	-1.526	1.0	-0.016	
	f	% Long Rally	Group - all	5	478	0.046	4.651	0.0	0.046	Mixed ANOVA
			Time Intervals - all	1	478	0.095	-16.734	1.0	-0.036	
			Interaction - all	5	478	0.025	-4.406	1.0	-0.048	
**S7**	d		Group - all	4	729	0.260	1.372	0.242	0.007	

		Average Rally Length	Time Intervals - all	1	729	2.355	-23.160	1.000	-0.033	Mixed ANOVA
			Interaction - all	4	729	0.525	-5.161	1.000	-0.029	
	e	% Aces	Group - all	4	729	0.066	1.445	0.217	0.008	Mixed ANOVA
			Time Intervals - all	1	729	0.141	-5.986	1.000	-0.008	
			Interaction - all	4	729	0.021	-0.897	1.000	-0.005	
	f	% Long Rally	Group - all	4	729	0.017	1.370	0.243	0.007	Mixed ANOVA
			Time Intervals - all	1	729	0.113	-17.552	1.000	-0.025	
			Interaction - all	4	729	0.024	-3.684	1.000	-0.021	
	p	Average Rally Length	Group - all	4	729	0.136	0.756	0.554	0.004	Mixed ANOVA
			Time Intervals - all	1	729	1.690	-17.577	1.000	-0.025	
			Interaction - all	4	729	0.663	-6.889	1.000	-0.039	
	q	% Aces	Group - all	4	729	0.032	0.712	0.584	0.004	Mixed ANOVA
			Time Intervals - all	1	729	0.054	-2.376	1.000	-0.003	
			Interaction - all	4	729	0.042	-1.838	1.000	-0.010	
	r	% Long Rally	Group - all	4	729	0.009	0.763	0.55	0.004	Mixed ANOVA
			Time Intervals - all	1	729	0.073	-11.682	1.00	-0.016	
			Interaction - all	4	729	0.032	-5.152	1.00	-0.029	
**S9**	a	Average Rally Length	Group - all	3	360	0.160	0.792	0.499	0.007	Mixed ANOVA
			Time Intervals - all	1	360	4.486	-38.506	1.000	-0.120	
			Interaction - all	3	360	0.286	-2.454	1.000	-0.021	
	b	% Aces	Group - all	3	360	0.033	0.844	0.471	0.007	Mixed ANOVA
			Time Intervals - all	1	360	0.162	-7.936	1.000	-0.023	
			Interaction - all	3	360	0.031	-1.503	1.000	-0.013	
	c	% Long Rally	Group - all	3	360	0.012	1.004	0.391	0.008	Mixed ANOVA
			Time Intervals - all	1	360	0.234	-36.162	1.000	-0.112	
			Interaction - all	3	360	0.003	-0.517	1.000	-0.004	

Table S4. Multivariate statistical tests and all results for tests done.

Figure	Panel	Parameters	Source	DF	MS	F	p-value	np2	Method
**8**	a	Average Paddle Movement	Group - all	4	1.064e+10	21.837	0.0	0.155	ANOVA
	b	Relative improvement (%) in the average hit counts	Group - all	4	104528.369	17.807	0.0	0.089	ANOVA
	c	Average Paddle Movement	Group - all	4	1.801e+10	49.523	0.0	0.293	ANOVA
	d	Relative improvement (%) in the average hit counts	Group - all	4	116698.296	16.243	0.0	0.082	ANOVA

	e	Average Paddle Movement	Group - all	4	1.009e+10	26.881	0.0	0.184	ANOVA
	f	Relative improvement (%) in the average hit counts	Group - all	4	79671.720	9.889	0.0	0.051	ANOVA
**9**	a	Relative improvement (%) in the average hit counts - DQN	Group - all	5	77257.903	10.241	0.0	0.097	ANOVA
	b	Relative improvement (%) in the average hit counts – A2C	Group - all	5	73239.513	11.211	0.0	0.105	ANOVA
	c	Relative improvement (%) in the average hit counts - PPO	Group - all	5	83698.926	9.517	0.0	0.091	ANOVA
**S8**	a	Relative improvement (%) in the average hit counts - Ball Position Input	Group - all	4	81200.989	10.941	0.0	0.057	ANOVA
	b	Relative improvement (%) in the average hit counts - Paddle&Ball Position Input	Group - all	4	125476.158	20.915	0.0	0.103	ANOVA
**S9**	d	Relative improvement (%) in the average hit counts – Active Inference	Group - all	3	52072.238	6.733	0.0	0.053	ANOVA
